# Thionitrosyl Complexes of Technetium and Rhenium with Sterically Encumbered *m*‑Terphenyl Isocyanides—Steric Bulk Matters

**DOI:** 10.1021/acs.organomet.5c00250

**Published:** 2025-08-19

**Authors:** Domenik Nowak, Guilhem Claude, Anna-Maria Tsirigoni, Adelheid Hagenbach, Joshua S. Figueroa, Ulrich Abram

**Affiliations:** † 9166 Freie Universität Berlin, Institute of Chemistry and Biochemistry, Fabeckstr. 34/36, Berlin D-14195, Germany; ‡ Department of Chemistry and Biochemistry, 8784 University of California San Diego, 9500 Gilman Drive, MC 0358, La Jolla, California 92093, United States

## Abstract

Low-valent thionitrosyl complexes of technetium and rhenium with the bulky isocyanides CNAr^Tripp2^, CNAr^Dipp2^, and CNAr^Mes2^ (Tripp = 2,4,6-triisopropylphenyl, Dipp = 2,6-diisopropylphenyl, Mes = 2,4,6-trimethylphenyl) have been synthesized by reactions of the corresponding nitridotechnetium­(V) or rhenium­(V) compounds with disulfur dichloride. The structures of the products and the oxidation states of the resulting metal ions are mainly controlled by the steric bulk of the terphenyl isocyanide ligands. Bis­(isocyanide) Tc­(II) and Re­(II) were obtained exclusively with CNAr^Tripp2^ or CNAr^Dipp2^, which is in contrast with the more diverse reaction outcomes with the less bulky CNAr^Mes2^, where the products also include metal­(I) and dimeric compounds. The obtained complexes [Re^V^NCl_2_(CNAr^Tripp2^)_2_(MeOH)], [Re^V^NCl_2_(CNAr^Dipp2^)_2_], [Re^V^NCl_2_(CNAr^Mes2^)_3_], [Tc^V^NCl_2_(CNAr^Tripp2^)_2_], [Re^II^(NS)­Cl_3_(CNAr^Tripp2^)_2_], [Re^II^(NS)­Cl_3_(CNAr^Dipp2^)_2_], [Re^I^(NS)­Cl_2_(CNAr^Mes2^)_3_], [Tc^II^(NS)­Cl_3_(CNAr^Tripp2^)_2_], [Tc^II^(NS)­Cl_3_(CNAr^Dipp2^)_2_], [Tc^II^(NS)­Cl_2_(CNAr^Mes2^)_3_]­Cl, and [{Tc­(NS)­Cl­(CNAr^Mes2^)_2_}_2_Cl_2_] were studied spectroscopically and partially by X-ray diffraction.

## Graphical abstract

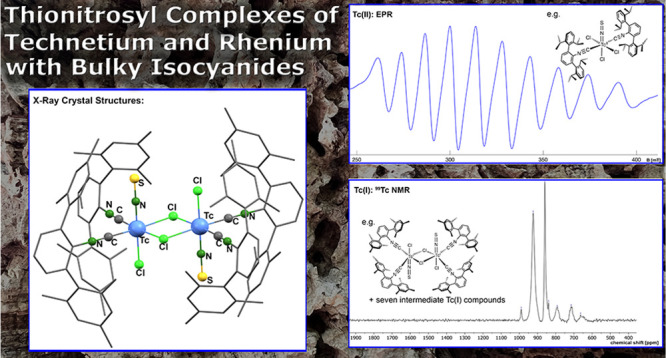

## Introduction

Thionitrosyl complexes are relatively rare compared with the vast number of transition metal nitrosyls.^1–5^ One of the main reasons for this lack of knowledge might be the absence of a ready synthetic approach to a monomeric nitrogen sulfide. Although some spectroscopic data of the NS**·** radical could be recorded and some salts containing NS^+^ cations have been isolated,^6–9^ such compounds found only occasionally application as precursors for the syntheses of thionitrosyl complexes.^1,8,9^ Thermal or photochemical decompositions of cyclic compounds such as tetrasulfur tetranitride, S_4_N_4_, or trithiazyltrichloride, (NSCl)_3_ (Chart 1) release NS^+^ or NSCl^+^ building blocks.^10–14^ But the handling at least of N_4_S_4_, which tends to explode on heating or upon shock, is potentially dangerous and consequently not favored for reactions with the radioactive element technetium.

**Chart 1:**
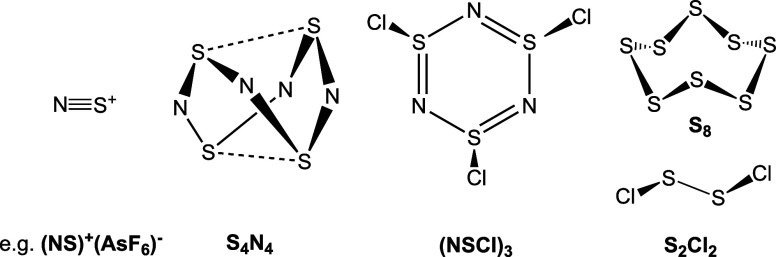
Common Precursor Molecules for the Synthesis of Thionitrosyl Complexes

Thus, another synthetic approach, the addition of a ‘sulfur atom’ to a nitrido ligand, seems to be more favorable. ‘Sulfur sources’ such as elemental sulfur,^15–21^ SOCl_2_,^22,23^ dithionite or NCS^–^ ligands have been used.^24,25^ Most successful, however, are reactions of nitrido complexes with S_2_Cl_2_. They give low-valent thionitrosyl species in good yields. The use of nitrido starting materials is also suitable for the synthesis of the relatively rare rhenium and technetium thionitrosyls, which frequently contain halide, phosphine, and/or pyridine-type coligands.^8–14,24–36^

Thionitrosyl complexes with isocyanide ligands have hitherto not been reported, which encouraged us to attempt some reaction with *m*-terphenyl isocyanides. Such encumbering ligands have been demonstrated to provide a high degree of steric protection. The modulation of their specific electronic properties over a wide range allow for the isolation of transition metal complexes featuring unusual ligands such as boron monofluoride or nitrous oxide.^37–50^ Importantly, isocyanides can function as strong σ-donors and π-acids, which enable isocyanides to form stable products with transition metal ions over a wide range of formal oxidation states. The latter fact has also been demonstrated for a series of rhenium and technetium complexes with the metals in the formal oxidation states from “+5” to “–1”.^51–58^ The isolated Tc­(V) nitrido complexes may act as potential starting materials for the synthesis of thionitrosyls.^59^ Consequently, we also prepared nitrido rhenium complexes with the isocyanides shown in Chart 2 and conducted reactions with S_2_Cl_2_ for the nitrido compounds of both metals.

**Chart 2:**
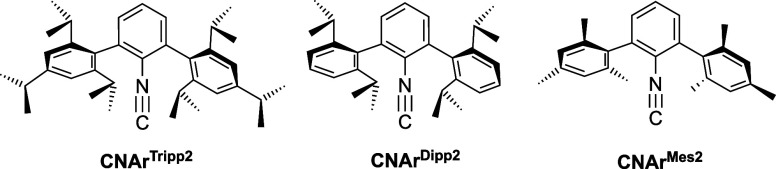
*m*-Terphenyl Isocyanides Used Throughout This Study

## Results and Discussion

Although there are generally different routes available for the synthesis of thionitrosyl complexes, only a few of the starting materials shown in Chart 1 are applicable for the elements technetium and rhenium. Some more restrictions are given by the reactivity of the isocyanides. At the first glance, ligand exchange procedures starting from [M­(NS)­Cl_4_]^−^ species or [M­(NS)­Cl_2/3_(PR_3_)_3/2_] complexes (M = Tc, Re) seem to be feasible. Such compounds are readily available by well-established procedures.^10,13,27,28,31,35^ But previous attempts to use them as starting materials for ongoing ligand exchange were only partially successful and gave clear results and good yields only with strong chelators.^35,36^ Thus, we decided to follow the other established synthetic route for the synthesis of thionitrosyl complexes: reactions of nitrido complexes with S_2_Cl_2_. This requires the synthesis of suitable isocyanide complexes with the transition metals in their high oxidations states. Previous studies have shown that the coordination chemistry particularly of the sterically encumbered isocyanides of Chart 2 is not exclusively dominated by their π-acceptor properties and a role as carbonyl mimics. The balanced electronic properties of such compounds together with steric protection also allow for the isolation of stable of rhenium­(V) and technetium­(V) complexes. Thus, series of oxorhenium­(V) complexes,^52^ and corresponding phenylimido species of both metals have been isolated and studied spectroscopically and by X-ray diffraction.^55,58^ There are also three of such nitridotechnetium­(V) complexes known, *trans*-[TcNCl_2_(CNAr^Tripp2^)_2_], *cis*-[TcNCl_2_(CNAr^Mes2^)_2_(H_2_O)] and *cis*-[TcNCl_2_(CNAr^Mes2^)_2_(MeOH)] (Chart 3).^59^ The arrangement of the isocyanides in the products seems to be dependent on their steric bulk. Corresponding rhenium compounds are hitherto unknown and have now been prepared as precursors for the corresponding thionitrosyls, as the respective technetium complex with CNAr^Tripp2^ (Scheme 1).

**Scheme 1:**
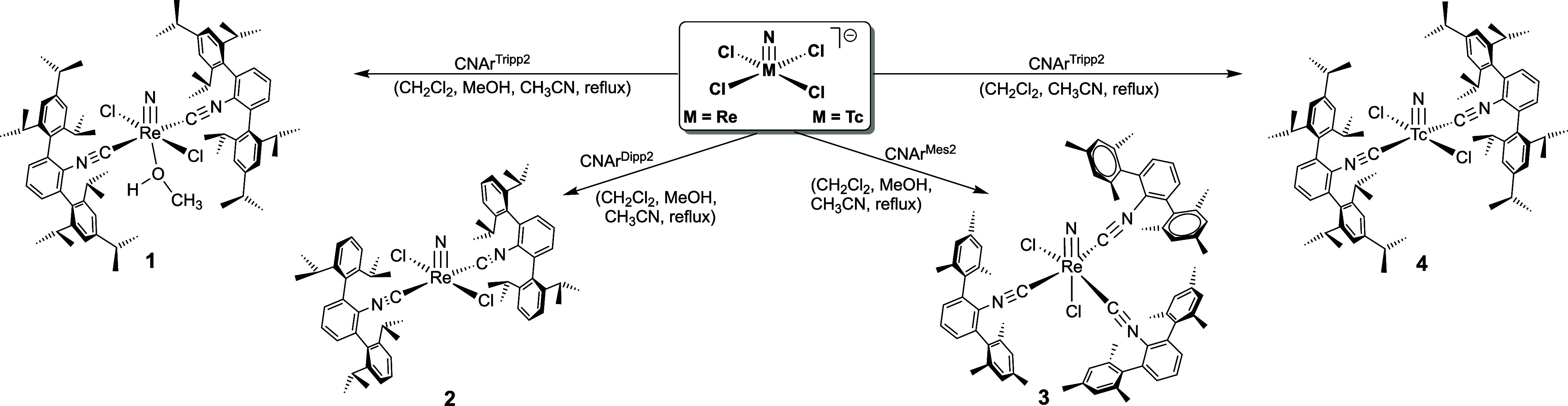
Syntheses of Re^V^ and Tc^V^ Nitrido Complexes with *m*-Terphenyl Isocyanides

**Chart 3:**
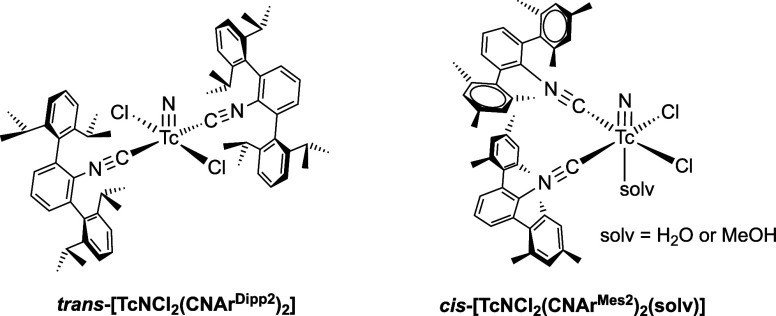
Previously Studied Nitridotechnetium­(V) Complexes with CNAr^Dipp2^ and CNAr^Mes2^ ^59^

Reactions starting from (NBu_4_)­[MNCl_4_] in boiling acetonitrile give orange-red, crystalline products in moderate to good yields. They are stable as solids and in solution. No problems appeared during the synthesis of [ReNCl_2_(CNAr^Tripp2^)_2_(MeOH)] (**1**), [ReNCl_2_(CNAr^Dipp2^)_2_] (**2**) and [TcNCl_2_(CNAr^Tripp2^)_2_] (**4**), while significantly lower yields were obtained for the product with the less bulky CNAr^Mes2^ ligand, [ReNCl_2_(CNAr^Mes2^)_3_] (**3**). Obviously, the steric encumbrance provided by CNAr^Tripp2^ and CNAr^Dipp2^ directs two of such ligands into the electronically less preferred *trans* positions to each other. Thus, the formation of coordination isomers is avoided and the exclusive formation of such products is ensured. The coordination position *trans* to the nitrido ligand is labile due to the triply bonded ligand and its occupation can vary. Frequently, halide ligands for charge compensation or weakly bonded solvent molecules coordinate there. It is also a common feature that this bonding site remains empty and five-coordinate nitrido complexes are formed.^59–75^ Thus, the coordination of a methanol ligand in the rhenium CNAr^Tripp2^ complex **1** is not unusual, even when the corresponding technetium complex **4** has a five-coordinate, square pyramidal structure. More serious is the influence of the steric bulk of the *m*-terphenyl isocyanides. This can be seen by the products formed with the less restricting ligand CNAr^Mes2^: *cis*-[TcNCl_2_(CNAr^Mes2^)_2_(solv)] (Chart 3) and *mer*-[ReNCl_2_(CNAr^Mes2^)_3_] (**3**). The yields obtained for complexes with this ligand are commonly low and an analysis of the spectroscopic data recorded for the corresponding reaction solutions indicates the formation of mixtures containing several products. It should be mentioned that similar observations also apply to the corresponding thionitrosyl complexes discussed vide infra. Thus, the isolated and structurally characterized complexes containing CNAr^Mes2^ may only represent a fraction of the formed products.

Another experimental proof for the crucial role of the steric bulk of the isocyanides is given by the reaction depicted in Scheme 2. A dimeric, nitrido-bridged [{TcCl­(CN^*t*^Bu)_4_}_2_(μ-N)]^+^ cation has been isolated as its [TcCl_6_]^2–^ salt (**5**) as one of the products of the interaction of [TcNCl_2_(PPh_3_)_2_] with *tert*-butyl isocyanide (CN^*t*^Bu) in boiling acetonitrile. The yield is low and compound **5** is most probably not the only product of the reaction. It is remarkable that technetium–nitrogen triple bonds are (at least partially) cleaved for the formation of the dimeric cation and the [TcCl_6_]^2–^ counteranion. The cleavage of Tc≡N bonds is a is quite uncommon feature and has previously only been observed in some rare cases.^74,76^

**Scheme 2:**
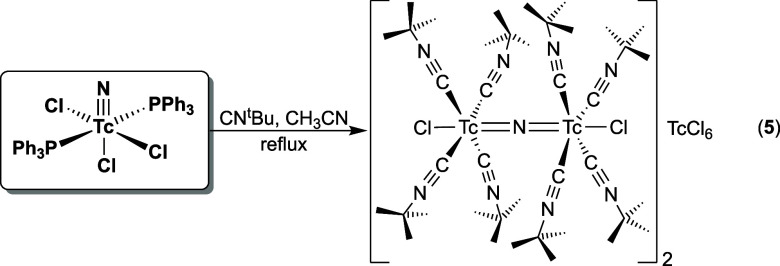
Dimeric Technetium Complex Isolated from a Reaction of [TcNCl_2_(PPh_3_)_2_] with CN^*t*^Bu

Figure 1 depicts the results of single-crystal X-ray studies on some of the newly isolated nitrido complexes introduced in Scheme 1. Together with the previously published structures of *trans*-[TcNCl_2_(CNAr^Mes2^)_2_] and *cis*-[TcNCl_2_(CNAr^Mes2^)_2_],^59^ they confirm that the steric bulk of the isocyanides is important for the composition of the formed complexes. Complexes with CNAr^Dipp2^ and CNAr^Tripp2^ contain two of such ligands in *trans*-position to each other, while the products with the less bulky ligand CNAr^Mes2^ are structurally more diverse. *cis*-Coordination of two isocyanides is established in the corresponding technetium complexes (see Chart 3), while three of such ligands are accommodated in *mer*-positions in the equatorial coordination sphere of the rhenium complex **3**. Less encumbering *m*-terphenyl isocyanide ligands such as CNAr^DArF2^ (DArF = 3,5-(CF_3_)_2_C_6_H_3_) even allow the coordination of four of such ligands in one plane as has been shown for corresponding manganese, technetium and rhenium complexes.^53,56,57^

**Figure 1:**
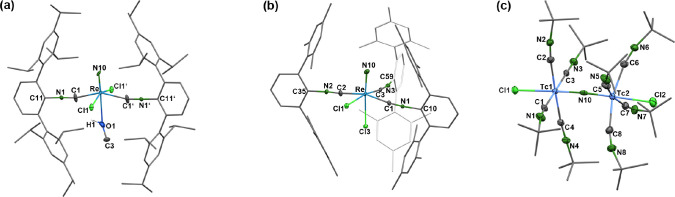
Molecular structures of (a) *trans*-[ReNCl_2_(CNAr^Tripp2^)_2_(MeOH)] (**1**), (b) *mer,cis*-[ReNCl_2_(CNAr^Mes2^)_3_] (**3**), and (c) [{TcCl­(CN^*t*^Bu)_4_}_2_(μ-N)]^+^ cation of compound **5**. Thermal ellipsoids represent 50% probability.

Some structural parameters of the nitrido compounds are contained in Table 1. The isocyanide ligands are linearly bonded with Tc–C≡N and C≡N–C angles between 163 and 177°. Larger deviations, which are occasionally observed for complexes with (very) low-valent metal ions,^53,56,57^ do not apply for the high-valent nitrido complexes under study. The bonds to the terminal nitrido ligands in complexes **1** and **3** are in the common range between 1.54 and 1.68 Å.

**Table 1:** Selected Bond Lengths (Å) and Angles (°) in the Nitrido Complexes **1**, **3**, and **5**

	1	3^a^	5
M–N10	1.54(1)	1.67(1), 1.677(9)	1.784(6), 1.770(6)
M–C	2.098(2)	2.071(3)–2.113(3)	2.062(7)–2.084(6)
M–Cl	2.363(1)	2.436(2)–2.530(4)	2.529(2), 2.502(2)
C≡N	1.141(1)	1.136(4)–1.142(4)	1.133(9)–1.17(1)
M–C≡N	168.5(2)	169.6(3)–171.1(2)	167.3(7)–174.2(7)
C–N–C	173.7(2)	167.8(3)–168.9(3)	163(1)–176.7(6)
Tc–N–Tc			175.7(3)

a Values for two independent species.

The nitrido bridge in the [{TcCl­(CN^*t*^Bu)_4_}_2_(μ-N)]^+^ complex cation is symmetrical with Tc–N distances of approximately 1.78 Å. Main structural features of the dimer are consistent with related binuclear nitrido complexes such as [{WCl_5_}_2_(μ-N)]^−^ or [(ReCl_5_)_2_(μ-N)]^3–^.^77,78^

Reactions of the rhenium­(V) and technetium­(V) nitrido complexes shown in Scheme 1 and Chart 2 with an excess of disulfur dichloride in dichloromethane result in the reduction of the metal ions and the formation of low-valent thionitrosyl complexes. The experimental procedure follows the standard approach, which has successfully been applied for a number of nitrido complexes with other ligand systems. The reactions can be performed at room temperature and give M­(II) or M­(I) complexes depending on the structures of the starting materials and the ligands applied. The individual products obtained are shown in Scheme 3.

**Scheme 3:**
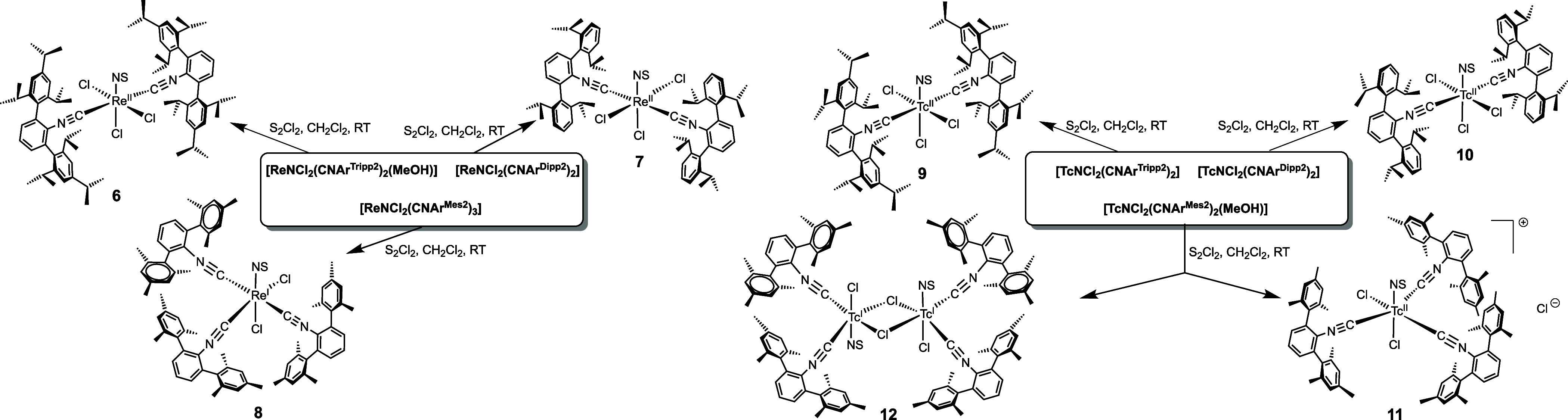
Syntheses of Thionitrosylrhenium and -Technetium Complexes with Sterically Encumbered *m*-Terphenyl Isocyanides

The paramagnetic Re­(II) and Tc­(II) products are dark orange-red to dark red, crystalline solids, while the metal­(I) complexes **8** and **12** are yellow or yellow-orange. All thionitrosyl complexes described in the present study are air-stable as solids and in solution. A spontaneous cleavage of the N–S bonds and reformation of the nitrido species, as was found for [Tc­(NS)­Cl_4_]^−^ and related compounds,^13^ was not observed. The ν_NS_ bands appear in the IR spectra of the products in the range between 1217 and 1247 cm^–1^. This is the typical range observed for thionitrosyl complexes,^2–4,2–4,16–19^ but also for the uncoordinated NS molecule.^79,80^ It is noteworthy, that the lowest values at 1217 cm^–1^ are found for the Re­(I) and Tc­(I) complexes and suggest an increased back-donation from the d^6^ metal ions (compared with the d^5^ ions of the metal­(II) compounds) into antibonding orbitals of the thionitrosyl ligands. Such findings are not completely unexpected with regard to similar results obtained for Tc­(I) and Tc­(II) nitrosyl complexes.^36,81–85^ On the basis of the spectroscopic data and MO considerations, it is also evident that the extend of the expected differences in the σ-donor and π-acceptor properties of nitrosyl and thionitrosyl ligands can vary. Oxidation state of the metal ions and coligands contained in the regarded species seem to have a considerable influence.^86–92^ Such conclusions have been confirmed by more recent DFT evaluations, where some better σ-donor properties have been assigned to NS^+^, while the corresponding π-acceptor behavior seems to be markedly influenced by coligands.^17,18,93–95^ The isocyanide ligands of the complexes of the present study are known likewise to act as π-acceptors. Thus, the inspection of the corresponding IR stretches might give an interesting insight into the electronic situation inside the high-valent nitrido and low valent thionitrosyl complexes compared with the values in the uncoordinated isocyanides. A summary of the experimental data is given in Table 2.

**Table 2:** Experimental ν_CN_ IR Stretches Observed for the *m*-Terphenyl Isocyanides and Their Nitrido and Thionitrosyl Complexes

	CNAr^Tripp2^	CNAr^Dipp2^	CNAr^Mes2^
ligand	2114	2124	2120
Re^V^N complexes	2149	2155	2160
Tc^V^N complexes	2172	2174^c^	2179^c^
Re^II^NS complexes	2190	2192	2145
Tc^II^NS complexes^a^	2202	2203	2126, 2037
Tc^I^NS complex^b^			2120

a Monomeric complexes **9**, **10**, and **11**.

b Dimeric complex **12**.

c Values taken from ref 59.

Although particularly aromatic isocyanides are known to act as efficient π-acceptors in many of their metal complexes,^96,97^ the situation in the compounds of the present study seems to be more elaborate. The infrared frequencies observed for the complexes do not display bathochromic shifts as a direct indicator for back-donation into antibonding ligand orbitals. This is easy to understand for the electron-deficient d^2^ systems of the nitrido compounds, but also applies for the d^5^ and d^6^ thionitrosyl complexes. Obviously, the ability of isocyanide ligands to accept back-donation vary with the nature of coligands competing for the electron density supplied by electron-rich metal ions. Similar results have been observed before for several carbonyl complexes of rhenium, technetium, molybdenum and nickel.^37,41,53,54,59,98–101^ Otherwise, the expected bathochromic shifts of the ν_CN_ bands relative to the values in uncoordinated ligands is a common feature in hexakis­(isocyanide) complexes of rhenium and technetium.^54,102–104^

Most of the thionitrosyl complexes of the present study are paramagnetic with a d^5^ “low spin” configuration, which corresponds to *S* = 1/2 spin systems. Thus, resolved EPR spectra in liquid and frozen solutions could be obtained. Interactions of the unpaired electron with the nuclear spins of the ^185,187^Re and ^99^Tc nuclei result in hyperfine couplings with the metal ions. Potential couplings with ^14^N of the thionitrosyl unit or ^35,37^Cl nuclei of the chlorido ligands are not resolved due their small numerical values and the considerable line-widths observed for the experimental EPR spectra. Such a finding is in a line with previously recorded solution-EPR spectra of Re­(II) or Tc­(II) nitrosyl or thionitrosyl complexes.^26–28,31,35,36,82,85^ Figure 2 shows exemplarily the spectra obtained for [Tc­(NS)­Cl_3_(CNAr^Tripp2^)_2_] (**9**). The experimental spectra of the other compounds are given as Supporting Information.

**Figure 2:**
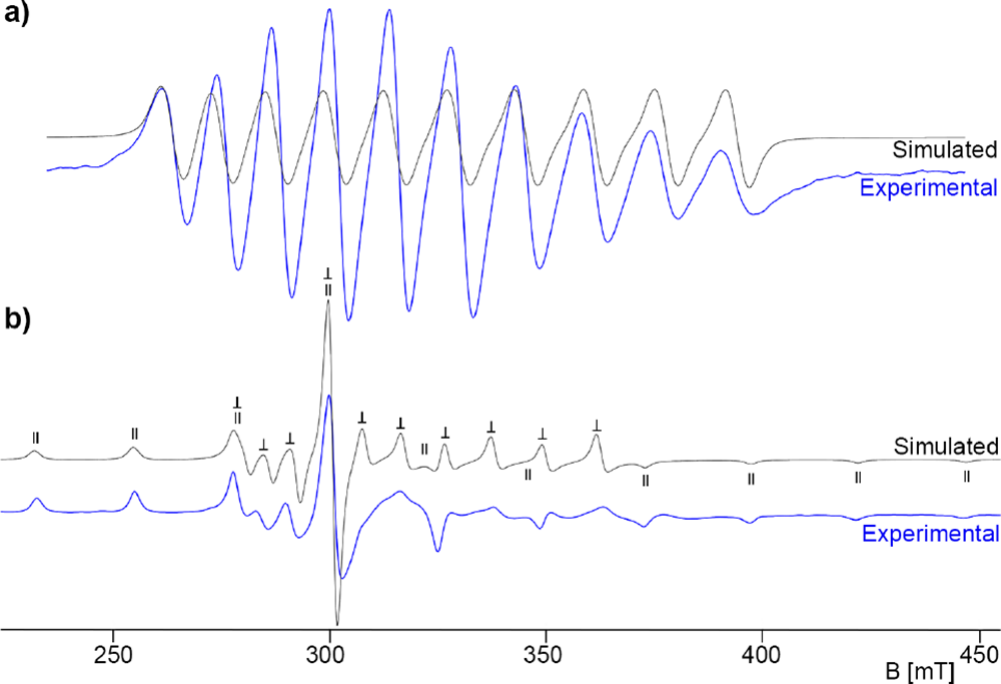
Liquid (a) and frozen (b) solution X-band EPR spectra of [Tc­(NS)­Cl_3_(CNAr^Tripp2^)_2_] (**9**) in CH_2_Cl_2_.

^99^Tc has a nuclear spin of *I* = 9/2, which gives a ten-line pattern in the spectrum in liquid solution. Two sets of ten ^99^Tc hyperfine lines are characteristic for the anisotropic frozen-solution spectrum, the general feature of which follows axial symmetry. The assignments of the individual lines to the parallel and perpendicular parts are indicated in the Figure 2. The spectra of the technetium complexes can be described by an axial-symmetric Spin Hamiltonian ([Disp-formula eq1]), where *g*_∥_, *g*_⊥_, *A*_∥_^Tc^, and *A*_⊥_^Tc^ are the principal values of the *g* tensor and the ^99^Tc hyperfine tensor *A* ^Tc^. The spectral parameters are summarized in Table 3.

$${Ĥ}_{\mathrm{sp}}=\beta_{e}[g_{∥}B_{z}{Ŝ}_{z}+g_{⊥}(B_{x}{Ŝ}_{x}+B_{y}{Ŝ}_{y})]+A_{∥}^{\mathrm{Tc}}{Ŝ}_{z}{Í}_{z}+A_{⊥}^{\mathrm{Tc}}({Ŝ}_{x}{Í}_{x}+{Ŝ}_{y}{Í}_{y})$$ 1

**Table 3:** EPR Parameters of the Paramagnetic Re­(II) and Tc­(II) Complexes^a^

	*g*_0_	*a*_0_^M^	*g*_z(∥)_	*g*_*x*,*y*(⊥)_	*A*_z(∥)_^M^	*A*_*x*,*y*(⊥)_^M^
**6**	2.009	355	1.880	2.300	530	190
				2.360^b^		210^b^
**7**	2.013	340	1.880	2.300	530	190
				2.350^b^		200^b^
**9**	2.034	135	2.019	2.120	225	89
**10**	2.035	137	2.024	2.118	225	91
**11**	2.011	159	1.970	2.020	260	115

a Coupling constants are given in 10^–4^ cm^–1^.

b Rhombic distortion.

The frozen-solution EPR spectra of the structurally similar bis­(isocyanide) complexes **9** and **10** are almost identical with slightly anisotropic *g* tensors. Their ^99^Tc hyperfine couplings are smaller than those observed in the spectrum of compound **11**, having three isocyanide ligands in the coordination sphere of technetium. This indicates that the unpaired electron shows weaker interactions with the ^99^Tc nucleus in the bis complexes accompanied by a larger degree of delocalization into ligand orbitals. This may be due to a smaller degree of electron acceptance caused by the nature of the ligands or by strong distortions in the coordination sphere. The latter reason results in a lower degree of orbital overlaps for compound **11,** which may be attributed to three of the bulky CNAr^Mes2^ ligands binding to the metal ion. Unfortunately, a direct proof by experimental angular data of an X-ray diffraction study is currently not possible by the lack of single crystals of appropriate quality.

The EPR spectra of the rhenium complexes [Re­(NS)­Cl_3_(CNAr^Tripp2^)_2_] (**6**) and [Re­(NS)­Cl_3_(CNAr^Dipp2^)_2_] (**7**) are very similar (see Supporting Material), but clearly differ from the structurally analogous technetium compounds **9** and **10** by showing a marked anisotropy of the *g* tensor and ^185,187^Re hyperfine tensors *A* ^Re^. As in the spectra of the technetium complexes, couplings of the unpaired electron with the metal ion (^185,187^Re, *I* = 5/2) are well resolved giving each six-line patters in the spectral components. The experimental spectra show some deviations from the axial symmetry and clearly indicate a rhombic distortion. The experimental data are summarized in Table 3 together with the those of the technetium compounds.

ESI+ mass spectra of the rhenium complexes support their compositions by the detection of ions belonging to the corresponding molecular ions. This also applies for the diamagnetic rhenium­(I) compound [Re­(NS)­Cl_2_(CNAr^Mes2^)_3_] (**8**), which is the only product, which could be isolated in pure from the reaction of [ReNCl_2_(CNAr^Mes2^)_3_] and S_2_Cl_2_. Mass spectra of technetium compounds could not be recorded for radiation protection reasons.

Figure 3 depicts structural plots of the novel thionitrosyl complexes **6**, **7**, **9**, and **10** studied by single-crystal X-ray diffraction. Selected bond lengths and angles are compared in Table 4. The observed M–N–S bonds are essentially linear, which is in agreement with the situation in all thionitrosyl complexes hitherto studied crystallographically.^105^ The M–N and N–S bond lengths are best interpreted as having some double bond character. Together with the spectroscopic data, this finding is in agreement with a treatment of the thionitrosyl units as NS^+^.

**Figure 3:**
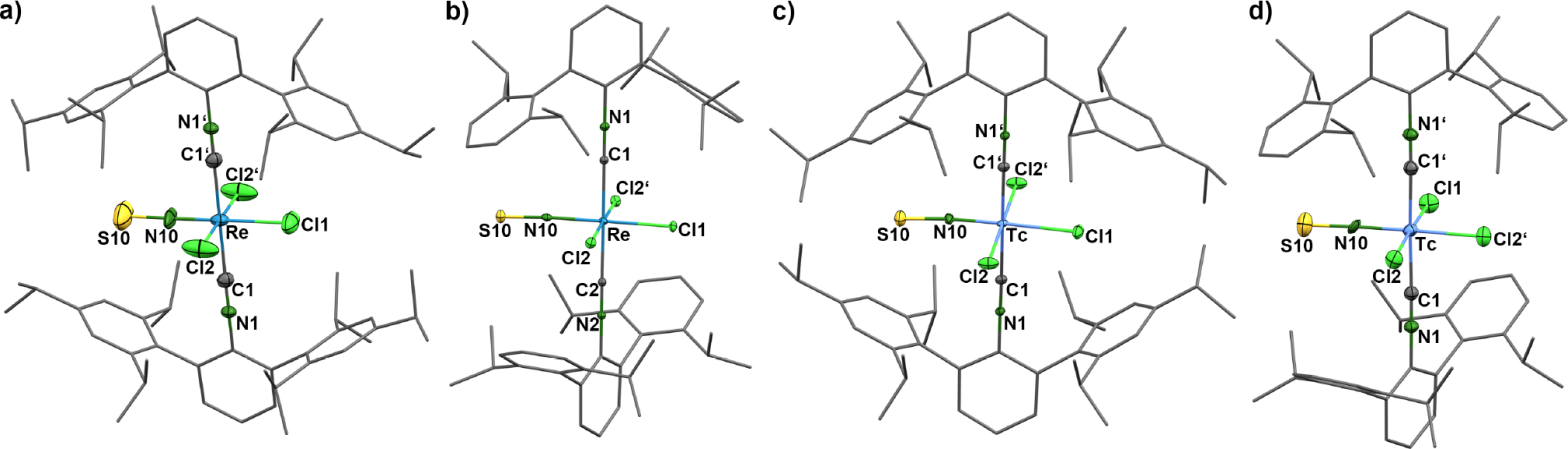
Molecular structures of (a) *trans*-[Re­(NS)­Cl_3_(CNAr^Tripp2^)_2_] (**6**), (b) *trans*-[Re­(NS)­Cl_3_(CNAr^Dipp2^)_2_] (**7**), (c) *trans*-[Tc­(NS)­Cl_3_(CNAr^Tripp2^)_2_] (**9**), and (d) *trans*-[Tc­(NS)­Cl_3_(CNAr^Dipp2^)_2_] (**10**). Thermal ellipsoids represent 30% probability.

**Table 4:** Selected Bond Lengths (Å) and Angles (°) in the Thionitrosyl Complexes **6**, **7**, **9**, and **10**

	6	7^a^	9	10^a^
M–N10	1.67(1)	1.811(5), 1.871(8)	1.707(4)	1.775(9), 1.814(9)
N10–S10	1.574(9)	1.521(8), 1.532(5)	1.540(4)	1.552(9), 1.553(9)
M–Cl	2.326(1), 2.386(5)	2.319(1)–2.3769(6)	2.3394(4), 2.422(2)	2.292(3)–2.3682(9)
M–C	2.110(4)	2.096(3)–2.104(3)	2.109(2)	2.111(4)–2.131(4)
C≡N	1.136(4)	1.144(4)–1.154(4)	1.145(2)	1.133(3)–1.151(5)
M–C≡N	178.8(3)	178.6(2), 180	177.3(1)	178.6(3), 180
C–N–C	176.0(4)	173.1(9), 180	174.6(1)	179.1(3), 180
M–N–S	178.6(8)	168.2(4), 169.5(7)	174.7(2)	166.9(9), 170.2(8)

a Values for two independent species.

An almost ideal linear coordination of the isocyanide ligands is found for all thionitrosyl complexes of the present study. M–C≡N angles between 177 and 180°, and C≡N–C angles between 173 and 180° are found. Significant bending, particularly of the C–N–C bonds, are typical for rhenium and technetium complexes with the metals in very low oxidation states. Small angles in the range of 150–160° have been found for such electron-rich M(0) and M(−1) compounds (M = Mn, Tc, Re).^51,53,56,57,106^ Even smaller C–N–C angles with values down to 120° have been detected for related molybdenum(0) complexes. But it should be mentioned that the situation in some of the molybdenum compounds is supported by the formation of chelate rings. They contain additional bonds to carbon atoms of multidentate isocyanides or interactions established between the π-systems the *m*-terphenyl ligands.^102,107,108^

The ligand exchange of the high-valent rhenium and technetium nitrido complexes with the extremely bulky isocyanide ligands CNAr^Tripp2^ or CNAr^Dipp2^ as well as reactions of the formed products with S_2_Cl_2_ proceed via clear pathways and deliver well-defined products in satisfactory yields. In contrast, analogous reactions with the related, but less bulky CNAr^Mes2^ are more complex and commonly yield a variety of products with different structures and oxidation states of the metal ions. One example has already been discussed before for the reactions of nitridotechnetium complexes with the less bulky *tert*-butyl isocyanide. It gave an unexpected, binuclear product with a nitrido bridge instead of well-defined monomers.

Similar uncertainties are observed for the reactions of [MNCl_2_(CNAr^Mes2^)_3_] complexes with S_2_Cl_2_. Only one rhenium­(I) product, [Re­(NS)­Cl_2_(CNAr^Mes2^)_3_] (**8**), could be isolated in moderate yields. Contrary, a variety of Tc­(II) and Tc­(I) products is formed during analogous reactions starting from the technetium complex [TcNCl_2_(CNAr^Mes2^)_2_(MeOH)]. This could be seen by monitoring the course of such reactions by EPR or NMR. This variety obviously hinders the isolation of larger amounts of pure products. Minor hints of impurities are still visible in the EPR spectrum of the isolated [Tc­(NS)­Cl_2_(CNAr^Mes2^)_3_]Cl and the formation of at least seven Tc­(I) species in the further course of the reaction has been detected by respective ^99^Tc NMR spectra. Both spectra are shown as Supporting Material.

Small amounts of one of the Tc­(I) species, the dimeric complex [{Tc­(NS)­Cl­(CNAr^Mes2^)_2_}_2_Cl_2_] (**12**), could be isolated in crystalline form and a single crystal X-ray analysis was conducted. The molecular structure of the compound is shown in Figure 4. It consist of two {Tc­(NS)­Cl­(CNAr^Mes2^)_2_}^+^ units with *cis*-coordinated isocyanides. They are connected by two chlorido ligands forming a planar, almost square central unit.

**Figure 4:**
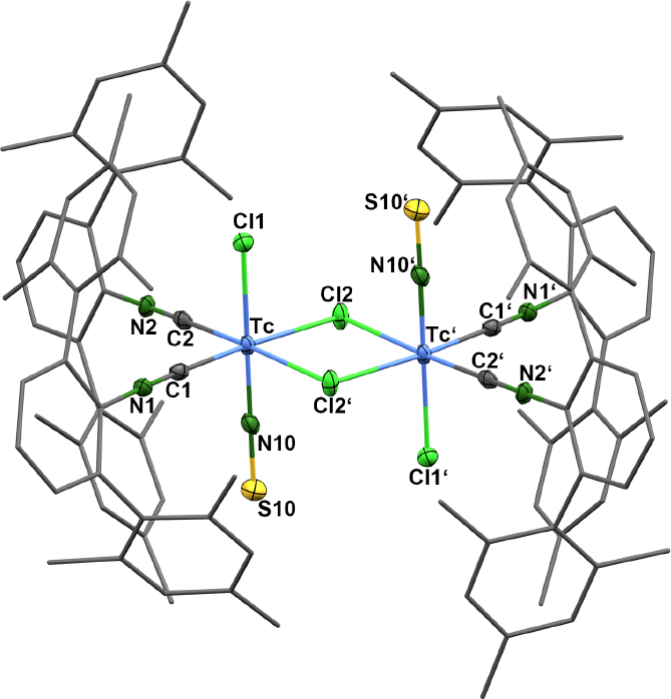
Ellipsoid plot of the molecular structure of [{Tc­(NS)­Cl­(CNAr^Mes2^)_2_}_2_Cl_2_] (**12**). Thermal ellipsoids represent 50% probability. Selected bond lengths (Å) and angles (°): Tc–C1:1.996(6), Tc–C2:1.993(5), C1–N1:1.159(7), C2–N2:1.149(7), Tc–N10:1.77(1), N10–S10:1.54(1), Tc–N10–S10:175.2(16), Tc–C1–N1:177.9(5), Tc–C2–N2:175.5(4), Tc–Cl2–Tc′: 95.43(4), Cl2–Tc–Cl2′: 84.58(4), Tc···Tc′: 3.673. Symmetry code: 1 – *x*, *y*, 1 – *z*.

A visualization of the importance of the steric bulk of the ligands is given in Figures 5 and 6. Figure 5 illustrates the importance of ‘virtually’ peripheral residues on the ligands (here CNAr^Dipp2^ vs CNAr^Tripp2^). Having a raw view to the molecular structures of the ligands, it might be surprising that the additional *i*-propyl residue in 4-position of CNAr^Tripp2^ has a significant contribution to the steric bulk of the ligand. The inspection of the structures of the related rhenium thionitrosyl complexes, however, clearly shows that this is indeed the case. The presence of these residues extents the shielding of the coordination spheres of the metal ions from two half-spheres to a torus. Reactions on the residual ligands of the coordination sphere remain possible as long as the M–C bonds remain rotatable. The top views to both complexes, however, clearly show that the coordination of a third of these isocyanide ligands is not possible.

**Figure 5:**
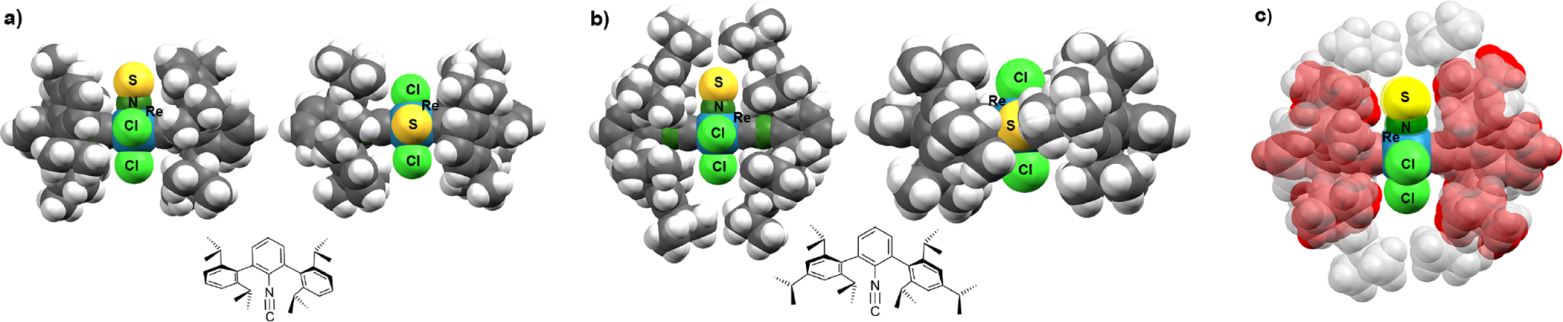
Side views and top views of space-filling models of the rhenium complexes **7** (a) and **6** (b) illustrate the degree of steric shielding due to the CNAr^Dipp2^ and CNAr^Tripp2^ ligands. Overlay of both structures (c), where the CNAr^Dipp2^ ligands are shown in red and the CNAr^Tripp2^ ligand in gray. The spheres represent each 75% of the van der Waals radii of the atoms.

**Figure 6:**
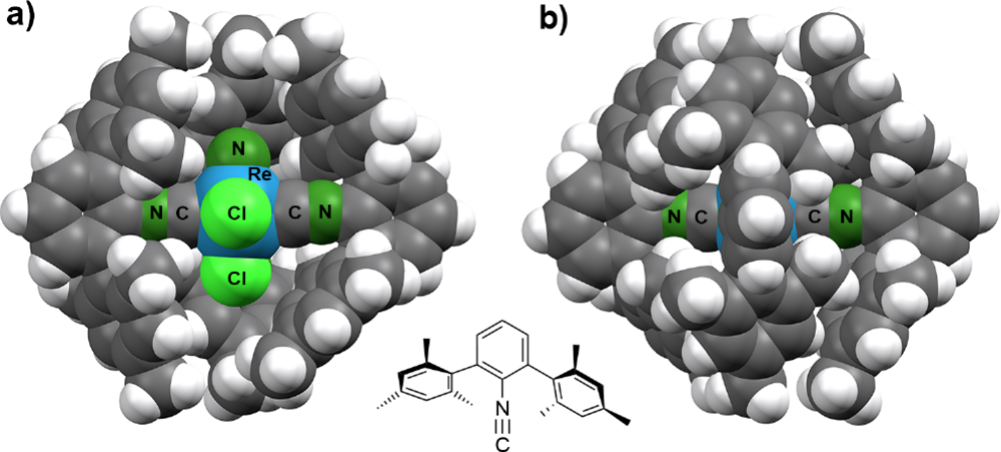
Space-filling model of the CNAr^Mes2^ complex **3** in front view (a) and back view (b).

Another situation is found in the corresponding CNAr^Mes2^ complexes. Figure 6 depicts space-filling projections of the nitrido complex **3** with three isocyanides in its coordination sphere. Expectedly, they are in meridional positions and particularly the ‘back-side view’ explains the limits of steric protection by this ligand. Although a tight packing of the coordination sphere is clearly shown, the bond angles around the rhenium atom do not reflect unusual distortions. The C1–Re–C3 and C2–Re–C3 angle are in the range of 90° and the relatively low C1–Re–C2 angle of 166.0(1)° is mainly due to the Re≡N triple bond, which causes *cis* angles between 94.5(4)° and 101.1(4)°.

## Conclusions

Rhenium­(V) and technetium­(V) nitrido complexes with sterically encumbered *m*-terphenyl isocyanides readily react with disulfur dichloride under formation of thionitrosyl species containing the transition metals in the oxidation states “+1” or “+2”. The structures of the products depend on the steric bulk of the ligands in a way that paramagnetic *trans*-[M^II^(NS)­Cl_3_(CNAr^Tripp2^)_2_] and *trans*-[M^II^(NS)­Cl_3_-(CNAr^Dipp2^)_2_] species are formed in moderate to good yields, while a large and less controllable variety of products are formed when the steric encumbrance of the isocyanides decreases. The isolated products with such ligands also involve monomeric and dimeric rhenium­(I) and technetium­(I) species as could be confirmed for the less bulky CNAr^Mes2^ ligand.

It would now be interesting to synthesize corresponding nitrosyl complexes for a comparison of analogous thionitrosyl and nitrosyl compounds. From such considerations, more information about the extend of electronic and steric influences of the individual CNAr^R2^ ligands on the structure and stability of the products are expected. Previous studies on carbonyl compounds including DFT calculations suggest an elaborate interplay between different parameters and strongly suggest a striking role of the position and nature of the residues attached to the *m*-terphenyl isocyanides.^54,59^ Corresponding nitrosyl compounds should be accessible at least for technetium and corresponding experiments are currently underway in our laboratories.

## Experimental Section

### Materials

All chemicals were reagent grade and used without further purification. ^99^Tc was purchased as solid ammonium pertechnetate from Oak Ridge National Laboratory (ORNL). The salt was purified by recrystallization from aqueous solutions. For this, the gray-black crystalline solid obtained from ORNL was dissolved in a minimum amount of warm water (60 °C) and filtered through a fine-porosity glass frit, leaving behind a very small amount of a black solid (TcO_2_·*n*H_2_O). The filtrate was brought to dryness by evaporation of the water, giving large colorless crystals of pure (NH_4_)­TcO_4_.^84^ CNAr^Tripp2^,^47^ CNAr^Dipp2^,^109^ CNAr^Mes2^,^110^ (NBu_4_)­[TcNCl_4_],^111^ and (NBu_4_)­[ReNCl_4_],^112^ and [TcNCl_2_(PPh_3_)_2_][Bibr ref111] were prepared by literature procedures.

### Physical Measurements

IR spectra were measured with a Shimadzu FTIR Affinity-1 spectrometer in KBr pellets (Tc compounds) and a Thermo Scientific Nicolet iS10 ATR spectrometer (ligands and Re complexes) between 400 and 4000 cm^–1^. NMR spectra were recorded on JEOL 400 MHz spectrometers. Chemical shifts (δ) are given relative to the signals of external standards (tetramethylsilane (^1^H), and NH_4_TcO_4_ in D_2_O (^99^Tc). X-Band EPR spectra were recorded in solution with a Magnettech Miniscope MS400 spectrometer at 300 and 77 K. Simulation and visualization of the EPR spectra were conducted with the EasySpin tool box in MATLAB.^113,114^ ESI mass spectra were measured with an Agilent 6210 ESI-TOF mass spectrometer. All MS results are given in the form of *m*/*z* assignment. Elemental analysis of carbon, hydrogen, nitrogen, and sulfur were determined using a Heraeus vario EL elemental analyzer. Combustion analyses could not be conducted for the radioactive technetium compounds for radiation protection reasons.

### Radiation Precautions

^99^Tc is a long-lived weak β^–^ emitter (*E*_max_ = 0.292 MeV). Normal glassware provides adequate protection against the weak beta radiation when milligram amounts are used. Secondary X-rays (bremsstrahlung) play a significant role only when larger amounts of ^99^Tc are handled. All manipulations were done in a laboratory approved for the handling of radioactive materials.

### X-ray Crystallography

Single crystal X-ray diffraction data were collected on a Bruker APEX 3 or a STOE IPDS II with Mo Kα radiation. Absorption corrections were carried out by multiscan or integration methods.^115,116^ Structure solution and refinement were performed with the SHELX program package embedded in the OLEX2 platform.^117–119^ Hydrogen atoms were placed at calculated positions and treated with the “riding model” option of SHELXL. The representation of molecular structures was done using the program MERCURY.^120^ Some remaining crystallographic problems are commented on in the respective.cif files and/or as Supporting Information.

Additional information on the structure determinations is contained in the Supporting Information and has been deposited with the Cambridge Crystallographic Data Centre.

### Syntheses of the Complexes

#### [ReNCl_2_(CNAr^Tripp2^)_2_(MeOH)] (1)

(NBu_4_)­[ReNCl_4_] (58 mg, 0.1 mmol) was dissolved in acetonitrile (2 mL) and CNAr^Tripp2^ (100 mg, 0.2 mmol) was added in a mixture of CH_2_Cl_2_ (2 mL) and MeOH (2 mL). The mixture was heated under reflux for 30 min, and the solvent was removed in vacuum. The residue was dissolved in CH_2_Cl_2_ (10 mL) and filtered over a silica pad. Finally, more methanol (2 mL) was added. Slow evaporation gave red crystals of the product. Yield: 77 mg (69%). Elemental analysis (finely powdered sample): calcd for C_75_H_102_Cl_2_N_3_ORe: C, 68.3; H, 7.8; N, 3.2%. Found: C, 70.0; H, 8.1; N, 3.5%. Please note that the carbon value of the elemental analysis is outside the recommended limits, what we assign to a instrumental problem since a contamination of the sample with residual ligand would also require significantly higher values for hydrogen and nitrogen. IR (ATR, cm^–1^): 2958(s), 2929­(m), 2867 (m), 2149­(vs) (ν_CN_), 1607­(m), 1569­(m), 1458(s), 1413­(m), 1384­(m), 1362­(m), 1319­(m), 1240­(w), 1171­(m), 1106­(m), 1086­(m), 1055­(m) (ν_ReN_), 1010­(w), 942­(m), 921­(w), 875(s), 807(s), 774­(m), 758­(m), 652­(m). ^1^H NMR (CDCl_3_, ppm): 7.47 (t, *J* = 8.2, 7.0 Hz, 2H, CH_ar_), 7.34 (d, *J* = 7.3 Hz, 4H, CH_ar_), 7.00 (s, 8H, CH_ar_), 2.84 (hept, *J* = 6.9 Hz, 4H, CH), 2.41 (hept, *J* = 6.9 Hz, 8H, CH), 1.23 (d, *J* = 6.9, 2.7 Hz, 24, CH_3_), 1.05 (dd, *J* = 6.9 Hz, 48H, CH_3_). MS (ESI+): *m*/*z* = 1291.716 [M–MeOH–Cl + CH_3_CN]^+^ (ber.: 1291.727), 1298.744 [M–Cl–MeOH + CH_3_CN + Li]^+^ (calcd. 1298.743).

#### [ReNCl_2_(CNAr^Dipp2^)_2_] (2)

The compound was prepared as described for complex **1** using 0.2 mmol of CNAr^Dipp2^ (85 mg, 0.2 mmol) Yield: 73 mg (65%). Elemental analysis (finely powdered and vacuum-dried sample): Calcd for C_62_H_74_Cl_2_N_3_Re: C, 66.6; H, 6.7; N, 3.8%. Found: C, 66.9; H, 7.0; N, 3.9%. IR (ATR, cm^–1^): 3377­(w), 3145­(m), 3053­(m), 2959­(vs), 2925(s), 2866(s), 2155­(vs) (ν_CN_), 1695­(w), 1585­(m), 1579­(m), 1461(s), 1437(s), 1409(s), 1383(s), 1361(s), 1328­(m), 1252­(m), 1178­(m), 1104­(w), 1090(s), 1056(s) (ν_ReN_), 1007­(m), 937­(w), 806(s), 796­(vs), 759­(vs), 689­(m), 583(s). ^1^H NMR (CDCl_3_, ppm): 7.51 (dd, *J* = 7.6 Hz, 2H, CH_ar_), 7.36–7.30 (m, 8H, CH_ar_), 7.18 (d, *J* = 7.8 Hz, 8H, CH_ar_), 2.45 (hept, *J* = 6.9 Hz, 8H, C–H), 1.13 (d, *J* = 6.9 Hz, 24H, CH_3_), 1.09 (d, *J* = 6.8 Hz, 24H, CH_3_). MS (ESI+): *m*/*z* = 1123.550 [M–Cl + CH_3_CN]^+^ (ber.: 1123.539), 1140.483 [M + Na]^+^ (calcd. 1140.471), 1156.454 [M + K]^+^ (calcd. 1156.445).

#### [ReNCl_2_(CNAr^Mes2^)_3_] (3)

The compound was prepared as described for complex **1** using 0.3 mmol of CNAr^Mes2^ (102 mg, 0.3 mmol). Recrystallization was done from a CH_2_Cl_2_/acetonitrile mixture. Yield: 42 mg (33%). Elemental analysis (finely powdered and vacuum-dried sample): calcd for C_75_H_75_Cl_2_N_4_Re: C, 69.8; H, 5.9; N, 4.3%. Found: C, 69.9; H, 5.9; N, 4.3%. IR (ATR, cm^–1^): 2917­(w), 2197­(vs) (ν_CN_), 2160­(vs) (ν_CN_), 1614­(m), 1574­(w), 1456­(m), 1412­(w), 1376­(w), 1274­(w), 1189­(w), 1163­(w), 1035(s) (ν_ReN_), 1007­(m), 816­(vs), 780­(m), 760­(vs), 738­(m), 606­(m), 573­(m). ^1^H NMR:7.49 (dt, *J* = 15.2, 7.6 Hz, 2H, CH), 7.24–7.21 (dd, *J* = 7.6, 2.4 Hz, 4H CH), 6.98 (s, 8H, CH), 6.91 (s, 1H, CH), 6.88–6.82 (m, 4H, CH), 6.73 (s, 2H, CH), 2.32 (d, *J* = 6.2 Hz, 18H), 2.27 (s, 3H), 2.06 (s, 9H), 2.03 (s, 12H), 2.00 (s, 6H), 1.97 (s, 6H). MS (ESI+): *m*/*z* = 1253.535 [M – Cl]^+^ (calcd. 1253.524).

#### [TcNCl_2_(CNAr^Tripp2^)_2_] (4)

(NBu_4_)­[TcNCl_4_] (50 mg, 0.1 mmol) was dissolved in a mixture of CH_2_Cl_2_ (1 mL) and acetonitrile (2 mL) and added to a solution of CNAr^Tripp2^ (102 mg, 0.2 mmol) in CH_2_Cl_2_. The mixture was heated under reflux for 30 min. The solvents were removed in vacuum and the resulting orange-red solid was redissolved in 2 mL CH_2_Cl_2_ and overlayered with pentane (2 mL). Slow diffusion of the solvents gave orange-red microcrystals, which were filtered off and dried in vacuum. Yield: 98 mg (82%). IR (KBr, cm^–1^): 2961­(vs), 2928(s), 2868­(m), 2178­(vs) (ν_CN_), 1607­(m), 1570­(w), 1460(s), 1418­(m), 1385­(w), 1362­(w), 1317­(w), 1172­(w), 1105­(w), 1067­(w), 1043­(m) (ν_TcN_), 943­(w), 878­(m), 806­(m), 758­(w), 580­(w), 463­(w). ^1^H NMR (CDCl_3_, ppm): 7.51 (m_c_, 2H, CH_ar_), 7.33 (m_c_, 4H, CH_ar_), 6.99 (s, 8H, CH_ar_), 2.85 (h, *J* = 6.8 Hz, 4H, CH), 2.39 (h, *J* = 6.6 Hz, 8H, CH), 1.24 (d, *J* = 6.9, 24H, CH_3_), 1.07 (d, *J* = 6.9, 48H, CH_3_).

#### [{TcCl­(CN^*t*^Bu)_4_}_2_(μ-N)]_2_[TcCl_6_] (5)

CN^*t*^Bu (110 mg, 150 μL, 1.2 mmol) was added to a suspension of [TcNCl_2_(PPh_3_)_2_] (142 mg, 0.2 mmol) in MeCN (3 mL) and the mixture was heated under reflux for 2 h. All volatiles were removed in vacuum. The remaining orange-red residue was dissolved in CH_2_Cl_2_ and overlayered with toluene, which gave an orange-red powder. The solid was filtered off, washed with diethyl ether and redissolved in a small amount of CH_2_Cl_2_. Overlayering with diethyl ether and diffusion of the solvents gave two different types of crystals: orange-red needles of compound **5** and colorless blocks of [NH_2_PPh_3_]­Cl. They were separated manually. Yield: 40 mg (52%). IR (KBr, cm^–1^): 2980­(m), 2933­(w), 2130­(vs) (ν_CN_), 1454­(m), 1369(s), 1234­(m), 1191­(vs), 936­(vs), 860­(m), 730­(w).

#### [Re­(NS)­Cl_3_(CNAr^Tripp2^)_2_] (6)

[ReNCl_2_(CNAr^Tripp2^)_2_] (30 mg, 0.025 mmol) was dissolved in CH_2_Cl_2_ (10 mL) and S_2_Cl_2_ (0.3 mL) was added. The mixture was stirred at room temperature for 30 min and all volatiles were removed in vacuum. The remaining orange-red solid was dissolved in CH_2_Cl_2_ (2 mL) and the solution was overlayered with *n*-hexane. Slow diffusion of the solvents gave red blocks. Yield: 27 mg (87%). Elemental analysis: calcd for C_74_H_98_Cl_3_N_3_ReS: C, 65.6; H, 7.3; N, 3.1%. Found: C, 64.9; H, 7.0; N, 3.4%. IR (ATR, cm^–1^): 2957­(vs), 2926­(m), 2866­(m), 2190­(vs) (ν_CN_), 1608­(m), 1571­(m), 1457(s), 1428­(w), 1412­(w), 1384­(m), 1362­(m), 1320­(m), 1273­(w), 1239­(vs) (ν_NS_), 1175­(m), 1105­(w), 1069­(w), 1055­(w), 943­(w), 920­(w), 874(s), 851­(w), 824­(w), 807(s), 772­(m), 759(s), 652­(m), 582­(m), 556­(m). EPR (RT, CHCl_3_): *g*_0_ = 2.009; *a*_0_^Re^ = 351 × 10^–4^ cm^–1^. EPR (77 K, CH_2_Cl_2_) *g*_*x*_ = 2.300, *g*_*y*_ = 2.360, *g*_*z*_ = 1.880; *A*_*x*_^Re^ = 190 × 10^–4^ cm^–1^, *A*_*y*_^Re^ = 210 × 10^–4^ cm^–1^, *A*_*z*_^Re^ = 530 × 10^–4^ cm^–1^. MS (ESI+): *m*/*z* = 1359.65 [M + Li]^+^ (calcd. 1359.626), 1375.609 [M + Na]^+^ (calcd. 1375.609), 1391.581 [M + K]^+^ (calcd. 1391.574).

#### [Re­(NS)­Cl_3_(CNAr^Dipp2^)_2_] (7)

The compound was prepared following the procedure described for complex **6** using [ReNCl_2_(CNAr^Dipp2^)_2_] (28 mg, 0.025 mmol) as starting material. Orange-red crystals, yield: 28 mg (90%). Elemental analysis: calcd for C_62_H_74_Cl_3_N_3_ReS: C, 62.8; H, 6.3; N, 3.5%. Found: C, 63.2; H, 6.2; N, 3.6%. IR (ATR, cm^–1^): 2959­(vs), 2925­(m), 2866­(m), 2192(s) (ν_CN_), 1594(s), 1580­(m), 1524(s), 1501­(m), 1461(s), 1440­(m), 1409­(m), 1383(s), 1361(s), 1349­(m), 1308­(w), 1288­(w), 1228­(vs) (ν_NS_), 1177­(w), 1177­(m), 1118­(m), 1055­(m), 1018­(m), 954­(m), 928­(m), 872­(m), 852­(m), 824­(vs), 804(s), 797(s), 752­(vs), 682­(m), 632­(m), 603­(m), 593­(m). EPR (RT, CHCl_3_): *g*_0_ = 2.013; *a*_0_^Re^ = 340 × 10^–4^ cm^–1^. EPR (77 K, CH_2_Cl_2_): *g*_*x*_ = 2.300, *g*_*y*_ = 2.350, *g*_*z*_ = 1.880; *A*_*x*_^Re^ = 190 × 10^–4^ cm^–1^, *A*_*y*_^Re^ = 200 × 10^–4^ cm^–1^, *A*_*z*_^Re^ = 530 × 10^–4^ cm^–1^. MS (ESI+): *m*/*z* = 1191.445 [M + Li]^+^ (calcd. 1191.438), 1207.409 [M + Na]^+^ (calcd. 1207.412), 1223.380 [M + K]^+^ (calcd. 1223.386).

#### [Re­(NS)­Cl_2_(CNAr^Mes2^)_3_] (8)

The compound was prepared following the procedure described for complex **6** using [ReNCl_2_(CNAr^Mes2^)_3_] (32 mg, 0.03 mmol) as starting material. Orange-yellow crystals, yield: 24 mg (77%). Elemental analysis: calcd for C_75_H_75_Cl_2_N_4_ReS: C, 68.2; H, 5.7; N, 4.2%. Found: C, 67.9; H, 6.0; N, 4.1%. IR (ATR, cm^–1^): 2946­(m), 2916­(m), 2854­(w), 2184­(w), 2145 (vs) (ν_CN_), 2111­(vs) (ν_CN_), 1613­(m), 1575­(w), 1455­(m), 1414­(w), 1376­(w), 1273­(w), 1217(s) (ν_NS_), 1071­(w), 1032­(m), 849(s), 804­(m), 783­(w), 754(s), 737­(w), 606­(w). ^1^H NMR (CDCl_3_, ppm) 7.32–7.37 (t, J = 7.6 Hz, 3H), 7.14–7.19 (m, 6H, CH), 7.01 −6.96 (m, 12H), 2.32–2.35 (m, 18H, CH_3_), 2.01–2.05 (m, 36H, CH_3_). MS (ESI+): *m*/*z* = 1343.457 [M + Na]^+^ (calcd. 1343.454), 1359.441 [M+K]^+^ (calcd. 1359.428).

#### [Tc­(NS)­Cl_3_(CNAr^Tripp2^)_2_] (9)

[TcNCl_2_(CNAr^Tripp2^)_2_] (24 mg, 0.02 mmol) was dissolved in CH_2_Cl_2_ (10 mL) and S_2_Cl_2_ (0.3 mL) was added. The resulting dark red solution was stirred for 30 min at room temperature. The addition of 20 mL of *n*-hexane resulted in the precipitation of a dark red solid, which was subsequently washed by *n*-hexane and diethyl ether. Recrystallization from CH_2_Cl_2_/*n*-hexane gave dark red crystals. Yield: 20 mg (77%). IR (KBr, cm^–1^): 2960­(vs), 2929(s), 2869(s), 2202(s) (ν_CN_), 1607­(m), 1461(s), 1384­(m), 1362­(m), 1320­(w), 1247(s) (ν_NS_), 1173­(m), 1106­(m), 1056­(m), 942­(m), 876­(m), 807­(m), 761­(m). EPR (RT, CHCl_3_) *g*_0_ = 2.034; *a*_0_^Tc^= 135 × 10^–4^ cm^–1^. EPR (77 K, CH_2_Cl_2_): *g*_∥_ = 2.019, *g*_⊥_ = 2.120; *A*_∥_^Tc^ = 225 × 10^–4^ cm^–1^, *A*_⊥_^Tc^ = 89 × 10^–4^ cm^–1^.

#### [Tc­(NS)­Cl_3_(CNAr^Dipp2^)_2_] (10)

The compound was prepared as described for the synthesis of compound **9** using [TcNCl_2_(CNAr^Dipp2^)_2_] (21 mg, 0.02 mmol) as starting material. Red crystals, yield: 18 mg (83%). IR (KBr, cm^–1^): 2924­(m), 2866­(m), 2368­(w), 2203­(vs) (ν_CN_), 2127­(m), 1584­(w), 1572­(w), 1462(s), 1384­(w), 1236­(vs) (ν_NS_), 1175­(w), 1051­(m), 880­(w), 800­(m), 756­(w). EPR (RT, CHCl_3_) *g*_0_ = 2.035; *a*_0_^Tc^ = 137 × 10^–4^ cm^–1^. EPR (77 K, CH_2_Cl_2_): *g*_∥_ = 2.120, *g*_⊥_ = 2.118; *A*_∥_^Tc^ = 225 × 10^–4^ cm^–1^, *A*_⊥_^Tc^ = 91 × 10^–4^ cm^–1^.

#### [Tc­(NS)­Cl_2_(CNAr^Mes2^)_3_]Cl (11)

The compound was prepared as described for the synthesis of compound **9** using [TcNCl_2_(CNAr^Mes2^)_3_] (24 mg, 0.02 mmol) as starting material. Recrystallization was attempted from CH_2_Cl_2_/*n*-hexane solutions. Red powder, yield: 7 mg (31%). IR (KBr, cm^–1^): 2922(s), 2857­(m), 2126(s) (ν_CN_), 2037(s) (ν_CN_), 1721 (m), 1624­(m), 1439(s), 1380­(w), 1281­(m), 1236­(m), 1177(s), 1123­(m), 1069­(w), 853­(m), 806­(w), 727­(m), 691­(m), 581­(m), 538(s), 453­(m). EPR (RT, CHCl_3_) *g*_0_ = 2.011; *a*_0_^Tc^ = 159 × 10^–4^ cm^–1^. EPR (77 K, CH_2_Cl_2_): *g*_∥_ = 1.970, *g*_⊥_ = 2.020; *A*_∥_^Tc^ = 260 × 10^–4^ cm^–1^, *A*_⊥_^Tc^ = 115 × 10^–4^ cm^–1^.

#### [{Tc­(NS)­Cl­(CNAr^Mes2^)_2_}_2_Cl_2_] (12)

[TcNCl_2_(CNAr^Mes2^)_3_] (24 mg, 0.02 mmol) was dissolved in CH_2_Cl_2_ (5 mL) and S_2_Cl_2_ (0.3 mL) was added. The resulting dark orange solution was stirred for 10 min at room temperature. Volatiles were removed under reduced pressure and the obtained brown residue was extracted with diethyl ether. The obtained orange-red solution was filtered and *n*-hexane was added. Slow evaporation of the solution lead to the formation of few orange yellow crystals suitable for X-ray diffraction. IR (KBr, cm^–1^): 3384­(m), 2917(s), 2161(s) (ν_CN_), 2119(s) (ν_CN_), 1613­(m), 1507­(m), 1440(s), 1376­(m), 1276­(w), 1218­(m), 1096­(w), 878­(w), 847(s), 803­(m), 755­(m), 554­(w), 527­(w).

## Supplementary Material



## Notes

Collection of X-ray and spectroscopic data with tables containing crystallographic details, including full listings of bond lengths and angles, ellipsoid plots of the molecular structures, including disorders, selected IR, NMR, EPR, and MS spectra (PDF)
