# Supplementary material for: Thionitrosyl Complexes of Technetium and Rhenium with Sterically Encumbered m‑Terphenyl Isocyanides—Steric Bulk Matters
Source: Organometallics. 2025 Aug 19;44(17):1965–76. doi: 10.1021/acs.organomet.5c00250 (PMC12421688; doi:10.1021/acs.organomet.5c00250)
Supplement: Supplementary file 1 [file om5c00250_si_001.pdf]

Supplementary Materials to the paper entitled:

Thionitrosyl Complexes of Technetium and Rhenium with Sterically Encumbered *m*-Terphenyl Isocyanides – Steric Bulk Matters.

Domenik Nowak,[1] Guilhem Claude,[1] Anna-Maria Tsirigoni,[1] Adelheid Hagenbach,[1] Joshua S. Figueroa,[2*] and Ulrich Abram[1*]

[1)]Institute of Chemistry and Biochemistry, Freie Universität Berlin, Fabeckstr. 34/36, 14195 Berlin, Germany. email: ulrich.abram@fu-berlin.de

[2)]Department of Chemistry and Biochemistry, University of California San Diego, 9500 Gilman Drive, MC 0358, La Jolla, CA 92093, USA. Email: jsfig@ucsd.edu

Table of content

1. Crystallographic Data **S-4**

Table S1. Crystallographic data and data collection parameters. S-4

Figure S1. Ellipsoid representation of the structure of [ReNCl$_2$(CNAr^{Tripp2})$_2$(MeOH)] (**1**), also illustrating the disorder between two Re positions, and the N^{3-} and the methanol ligands in the axial positions. The thermal ellipsoids are set at a 30% probability level. Hydrogen atoms bonding to carbon atoms are omitted for clarity. b) Unit cell plot. S-8

Figure S2. Unit cell plot.of [ReNCl$_2$(CNAr^{Tripp2})$_2$(MeOH)] (**1**). S-8

Table S2. Bond lengths (Å) in [ReNCl$_2$(CNAr^{Tripp2})$_2$(MeOH)] (**1**). S-9

Table S3. Bond angles (°) in [ReNCl$_2$(CNAr^{Tripp2})$_2$(MeOH)] (**1**). S-9

Figure S3. Ellipsoid representation of the structure of [ReNCl$_2$(CNAr^{Mes})$_3$] (**3**) x 2.25 CH_3CN x 0.25 CH_2Cl_2, illustrating both crstallographically independent molecules of the complex. Also note the disorders of the atoms in the axial positions of both complex molecules and the disorder of one of the solvent acetonitrile molecules with CH_2Cl_2. The thermal ellipsoids are set at a 30% probability level. Hydrogen atoms are omitted for clarity. S-10

Figure S4. Unit cell plot of [ReNCl$_2$(CNAr^{Mes})$_3$] (**3**) x 2.25 CH_3CN x 0.25 CH_2Cl_2. .... S-11

Table S4. Bond lengths (Å) in [ReNCl$_2$(CNAr^{Mes})$_3$] (**3**) x 2.25 CH_3CN x 0.25 CH_2Cl_2. .... S-11

Table S5. Bond angles (°) in [ReNCl$_2$(CNAr^{Mes})$_3$] (**3**) x 2.25 CH_3CN x 0.25 CH_2Cl_2. .... S-13

Figure S5. Ellipsoid representation of the structure of [{TcCl(CN^{t}Bu)$_4$}$_2$(μ-N)][TcCl$_6$] (**5**) x 2 MeOH. Also note the disorders of the atoms in the [TcCl$_6$]$^{2-}$ anion and in some of the *tert.*butyl groups. The thermal ellipsoids are set at a 30% probability level. Hydrogen atoms are omitted for clarity. S-16

Figure S6. Unit cell plot of [{TcCl(CN^{t}Bu)$_4$}$_2$(μ-N)][TcCl$_6$] (**5**) x 2 MeOH. S-16

Table S6. Bond lengths (Å) in [{TcCl(CN^{t}Bu)$_4$}$_2$(μ-N)][TcCl$_6$] (**5**) x 2 MeOH. S-17

Table S7. Bond angles (°) in [{TcCl(CN^{t}Bu)$_4$}$_2$(μ-N)][TcCl$_6$] (**5**) x 2 MeOH S-17

Figure S7. Ellipsoid representation of the structure of [Re(NS)Cl_3($CNAr^{Tripp2}$)$_2$] (**6**), also illustrating disorders of the ligands in axial positions and in some of the isopropyl groups. The thermal ellipsoids are set at a 30% probability level. Hydrogen atoms are omitted for clarity. . S-19

Figure S8. Unit cell plot of [Re(NS)Cl_3($CNAr^{Tripp2}$)$_2$] (**6**). S-19

Table S8. Bond lengths (Å) in [Re(NS)Cl_3($CNAr^{Tripp2}$)$_2$] (**6**). S-20

Table S9. Bond angles (°) in [Re(NS)Cl_3($CNAr^{Tripp2}$)$_2$] (**6).** S-20

Figure S9. Ellipsoid representation of the structure of [Re(NS)Cl_3($CNAr^{Dipp2}$)$_2$] (**7**), illustrating both crystallographically independent molecules of the complex. Also note the disorders of the atoms in the central coordination planes of both complex molecules. The thermal ellipsoids are set at a 30% probability level. Hydrogen atoms are omitted for clarity. S-21

Figure S10. Unit cell plot of [Re(NS)Cl_3($CNAr^{Dipp2}$)$_2$] (**7**). S-22

Table S10. Bond lengths (Å) in [Re(NS)Cl_3($CNAr^{Dipp2}$)$_2$] (**7**). S-22

Table S11. Bond angles (°) in [Re(NS)Cl_3($CNAr^{Dipp2}$)$_2$] (**7**). S-23

Figure S11. Ellipsoid representation of the structure of [Tc(NS)Cl_3($CNAr^{Tripp2}$)$_2$] (**9**), also illustrating the disorder found for the atoms in the axial positions. The thermal ellipsoids are set at a 30% probability level. Hydrogen atoms are omitted for clarity. S-25

Figure S12. Unit cell plot of [Tc(NS)Cl_3($CNAr^{Tripp2}$)$_2$] (**9**). S-25

Table S12. Bond lengths (Å) in [Tc(NS)Cl_3($CNAr^{Tripp2}$)$_2$] (**9**). S-26

Table S13. Bond angles (°) in [Tc(NS)Cl_3($CNAr^{Tripp2}$)$_2$] (**9**). S-26

Figure S13. Ellipsoid representations of both crystallogrphically independent molecules of [Tc(NS)Cl_3($CNAr^{Dipp2}$)$_2$] (**10**) also illustrating the positional disorders inside the coordination spheres of the technetium atoms. The thermal ellipsoids are set at a 50% probability level. Hydrogen atoms are omitted for clarity. S-27

Figure S14. Unit cell plot of [Tc(NS)Cl_3($CNAr^{Dipp2}$)$_2$] (**10**). S-28

Table S14. Bond lengths (Å) in [Tc(NS)Cl_3($CNAr^{Dipp2}$)$_2$] (**10**). S-28

Table S15. Bond angles (°) in [Tc(NS)Cl_3($CNAr^{Dipp2}$)$_2$] (**10**). S-29

Figure S15. Ellipsoid representation of the structure of [{Tc(NS)Cl($CNAr^{Mes2}$)$_2$}$_2$$Cl_2$] (**12**) x 2 CH_2Cl_2, also illustrating the disorder of the axial ligands. The thermal ellipsoids are set at a 50% probability level. Hydrogen atoms are omitted for clarity. S-30

Figure S16. Unit cell plot of [{Tc(NS)Cl($CNAr^{Mes2}$)$_2$}$_2$$Cl_2$] (**12**) x 2 CH_2Cl_2. .... S-31

Table S16. Bond lengths (Å) in [{Tc(NS)Cl($CNAr^{Mes2}$)$_2$}$_2$$Cl_2$] (**12**) x 2 CH_2Cl_2. .... S-31

Table S17. Bond angles (°) in [{Tc(NS)Cl($CNAr^{Mes2}$)$_2$}$_2$$Cl_2$] (**12**) x 2 CH_2Cl_2. .... S-32

2. Selected Spectroscopic Data S-34

Figure S17. IR spectrum (ATR) of [ReNCl_2($CNAr^{Tripp2}$)$_2$(MeOH)] (**1**). S-34

Figure S18. ^{1}H NMR spectrum of [ReNCl_2($CNAr^{Tripp2}$)$_2$(MeOH)] (**1**) in $CDCl_3$. S-34

Figure S19. ESI+ mass spectrum of [ReNCl_2($CNAr^{Tripp2}$)$_2$(MeOH)] (**1**) in acetonitrile. S-34

Figure S20. IR spectrum (ATR) of [ReNCl_2($CNAr^{Dipp2}$)$_2$] (**2**). S-35

Figure S21. ^{1}H NMR spectrum of [ReNCl_2($CNAr^{Dipp2}$)$_2$] (**2**) in $CDCl_3$. S-35

Figure S22. ESI+ mass spectrum of [ReNCl_2($CNAr^{Dipp2}$)$_2$] (**2**) in acetonitrile. S-35

Figure S23. IR spectrum (ATR) of [ReNCl_2($CNAr^{Mes2}$)$_3$] (**3**). S-36

Figure S24. ^{1}H NMR spectrum of [ReNCl$_2$(CNAr^{Mes2})$_3$] (**3**) in CDCl$_3$.................................... S-36

Figure S25. ESI+ mass spectrum of [ReNCl$_2$(CNAr^{Mes2})$_3$] (**3**) in acetonitrile. S-36

Figure S26. IR spectrum (KBr) of [TcNCl$_2$(CNAr^{Tripp2})$_2$] (**4**)... S-37

Figure S27. ^{1}H NMR spectrum of [TcNCl$_2$(CNAr^{Tripp2})$_2$] (**4**) in CDCl$_3$.................................. S-37

Figure S28. IR spectrum (KBr) of [{TcCl(CN^{t}Bu)$_4$}$_2$(μ-N)]$_2$[TcCl$_6$] (**5**). S-37

Figure S29. IR spectrum (ATR) of [Re(NS)Cl$_3$(CNAr^{Tripp2})$_2$] (**6**)... S-38

Figure S30. X-Band EPR spectra of [Re(NS)Cl$_3$(CNAr^{Tripp2})$_2$] (**6**) in CHCl$_3$ a) at room-temperature and b) at 77 K.. S-38

Figure S31. ESI+ mass spectrum of [Re(NS)Cl$_3$(CNAr^{Tripp2})$_2$] (**6**) in acetonitrile. S-38

Figure S32. IR spectrum (ATR) of [Re(NS)Cl$_3$(CNAr^{Dipp2})$_2$] (**7**). S-39

Figure S33. X-Band EPR spectra of [Re(NS)Cl$_3$(CNAr^{Dipp2})$_2$] (**7**) in CHCl$_3$ a) at room-temperature and b) at 77 K.. S-39

Figure S34. ESI+ mass spectrum of [Re(NS)Cl$_3$(CNAr^{Dipp2})$_2$] (**7**) in acetonitrile.................... S-39

Figure S35. IR spectrum (ATR) of [Re(NS)Cl$_2$(CNAr^{Mes2})$_3$] (**8**)... S-40

Figure S36. ^{1}H NMR spectrum [Re(NS)Cl$_2$(CNAr^{Mes2})$_3$] (**8**) in CDCl$_3$................................. S-40

Figure S37. ESI+ mass spectrum of [Re(NS)Cl$_2$(CNAr^{Mes2})$_3$] (**8**) in acetonitrile. S-40

Figure S38. IR spectrum (KBr) of [Tc(NS)Cl$_3$(CNAr^{Tripp2})$_2$] (**9**).. S-41

Figure S39. X-Band EPR spectra of [Tc(NS)Cl$_3$(CNAr^{Tripp2})$_2$] (**9**) in CH$_2$Cl$_2$ a) at room-temperature and b) at 77 K.. S-41

Figure S40. IR spectrum (KBr) of [Tc(NS)Cl$_3$(CNAr^{Dipp2})$_2$] (**10**)... S-41

Figure S41. X-Band EPR spectra of [Tc(NS)Cl$_3$(CNAr^{Dipp2})$_2$] (**10**) in CH$_2$Cl$_2$ a) at room-temperature and b) at 77 K.. S-42

Figure S42. IR spectrum (KBr) of [Tc(NS)Cl$_2$(CNAr^{Mes2})$_3$]Cl (**11**). S-42

Figure S43. X-Band EPR spectra of [Tc(NS)Cl$_2$(CNAr^{Mes2})$_3$]Cl (**11**) in CH$_2$Cl$_2$ a) at room-temperature and b) at 77 K. Arrows indicate the signals of a minor amount of a second Tc(II) complex. S-42

Figure S44. IR spectrum (KBr) of [{Tc(NS)Cl(CNAr^{Mes2})$_2$}$_2$Cl$_2$] (**12**). S-43

Figure S45. ^{99}Tc NMR spectrum of a reaction mixture between [TcNCl$_2$(CNAr^{Mes2})$_3$] and S$_2$Cl$_2$ in CH$_2$Cl$_2$, which finally allows the isolation of [{Tc(NS)Cl(CNAr^{Mes2})$_2$}$_2$Cl$_2$] (**12**). Note the intermediate appearance of seven Tc(I) complex species. S-43

1. Crystallographic Data

Table S1. Crystallographic data and data collection parameters.

	$[ReNCl_2(CNAr^{Tripp2})_2(MeOH)]$ (**1**)	$[ReNCl_2(CNAr^{Mes})_3]$ (**3**) x 2.25 CH_3CN x 0.25 CH_2Cl_2
Empirical formula	$C_{75}H_{102}Cl_2N_3ORe$	$C_{79.75}H_{82.25}Cl_{2.5}N_{6.25}Re$
Formula weight	1318.69	1403.09
Temperature/K	100.00	100.00
Crystal system	monoclinic	triclinic
Space group	$P2_1/c$	$P\bar{1}$
a/Å	12.7497(8)	14.130(8)
b/Å	17.1403(9)	23.694(16)
c/Å	16.8730(8)	23.76(2)
α/°	90	106.23(3)
β/°	106.881(2)	103.514(18)
γ/°	90	105.305(18)
Volume/Å^3	3528.4(3)	6949(9)
Z	2	4
ρ_{calc} / gcm^{-3}	1.241	1.341
μ / mm^{-1}	1.841	1.893
F(000)	1380.0	2888.0
Crystal size / mm^3	0.25 × 0.25 × 0.25	0.52 × 0.09 × 0.05
Radiation	MoKα (λ = 0.71073)	MoKα (λ = 0.71073)
2Θ range for data collection/°	4.098 to 54.25	4.016 to 54.34
Index ranges	$-16 \leq h \leq 16$, $-21 \leq k \leq 21$, $-21 \leq l \leq 21$	$-18 \leq h \leq 17$, $-30 \leq k \leq 30$, $-30 \leq l \leq 30$
Reflections collected	83196	289975
Independent reflections	7794 [R_{int} = 0.0484, R_{sigma} = 0.0212]	30784 [R_{int} = 0.0549, R_{sigma} = 0.0257]
Data/restraints/parameters	7794/0/403	30784/3/1680
Goodness-of-fit on F^2	1.100	1.202
Final R indexes [I>=2σ (I)]	R_1 = 0.0267, wR_2 = 0.0573	R_1 = 0.0397, wR_2 = 0.0762
Final R indexes [all data]	R_1 = 0.0306, wR_2 = 0.0590	R_1 = 0.0440, wR_2 = 0.0776
Largest diff. peak/hole / e Å^{-3}	0.50/-0.76	1.27/-1.22
Diffractometer	Bruker APEX-II CCD	Bruker APEX-II CCD
Remarks	-	The large voids between the bulky complex molecules are filled by solvent molecules, which could be resolved as 2.25 CH_3CN and 0.25 CH_2Cl_2 per complex
CCDC access code	2421707	2421708

Table S1. Crystallographic data and data collection parameters (continued)

	[{TcCl(CN^{t}Bu)$_4$}$_2$(μ-N)]$_2$[TcCl$_6$] (**5**) x 2 MeOH	[Re(NS)Cl$_3$(CNAr^{Tripp2})$_2$] (**6**)
Empirical formula	$C_{82}H_{152}Cl_{10}N_{18}O_2Tc_5$	$C_{74}H_{98}Cl_3N_3ReS$
Formula weight	2266.71	1354.16
Temperature/K	100	200.00
Crystal system	Monoclinic	monoclinic
Space group	$P2_1/n$	$P2_1/c$
a/Å	11.723(3)	12.876(3)
b/Å	20.448(4)	17.444(4)
c/Å	24.690(4)	17.198(3)
α/°	90	90
β/°	91.07(1)	106.53(3)
γ/°	90	90
Volume/Å^3	5917(2)	3703.2(14)
Z	2	2
ρ_{calc} / gcm^{-3}	1.272	1.214
μ / mm^{-1}	7.050	1.817
F(000)	2342.0	1410.0
Crystal size / mm^3	0.15 × 0.05 × 0.05	0.42 × 0.23 × 0.06
Radiation	CuKα (λ = 1.54178)	MoKα (λ = 0.71073)
2Θ range for data collection/°	5.612 to 153.738	6.592 to 48.998
Index ranges	$-14 \leq h \leq 14$, $-25 \leq k \leq 25$, $-30 \leq l \leq 30$	$-15 \leq h \leq 15$, $-20 \leq k \leq 20$, $-20 \leq l \leq 20$
Reflections collected	84845	18305
Independent reflections	10095 [R_{int} = 0.1350, R_{sigma} = 0.0856]	6130 [R_{int} = 0.0502, R_{sigma} = 0.0481]
Data/restraints/parameters	10095/30/528	6130/18/402
Goodness-of-fit on F^2	1.045	0.945
Final R indexes [I>=2σ (I)]	R_1 = 0.0740, wR_2 = 0.1546	R_1 = 0.0316, wR_2 = 0.0626
Final R indexes [all data]	R_1 = 0.1102, wR_2 = 0.1704	R_1 = 0.0592, wR_2 = 0.0691
Largest diff. peak/hole / e Å^{-3}	0.98/-0.76	0.51/-0.53
Diffractometer	Bruker APEX II	STOE IPDS
Remarks	-	-
CCDC access code	2429651	2421709

Table S1. Crystallographic data and data collection parameters (continued)

	$[Re(NS)Cl_3(CNAr^{Dipp2})_2]$ (**7**)	$[Tc(NS)Cl_3(CNAr^{Tripp2})_2]$ (**9**)
Empirical formula	$C_{62}H_{74}Cl_3N_3ReS$	$C_{74}H_{98}Cl_3N_3STc$
Formula weight	1185.85	1265.96
Temperature/K	100.00	105.00
Crystal system	monoclinic	monoclinic
Space group	C2/c	$P2_1/c$
a/Å	24.2156(19)	12.7502(9)
b/Å	24.1421(18)	17.1387(11)
c/Å	20.9430(17)	16.9797(13)
α/°	90	90
β/°	108.356(3)	107.029(3)
γ/°	90	90
Volume/Å^3	11620.6(16)	3547.8(4)
Z	8	2
ρ_{calc} / gcm^{-3}	1.356	1.185
μ / mm^{-1}	2.305	0.387
F(000)	4872.0	1346.0
Crystal size / mm^3	0.2 × 0.18 × 0.15	0.25 × 0.17 × 0.16
Radiation	MoKα (λ = 0.71073)	MoKα (λ = 0.71073)
2Θ range for data collection/°	4.268 to 54.998	4.1 to 54.2
Index ranges	$-31 \leq h \leq 31, -31 \leq k \leq 31, -27 \leq l \leq 27$	$-16 \leq h \leq 16, -21 \leq k \leq 21, -21 \leq l \leq 21$
Reflections collected	112467	76546
Independent reflections	13316 [R_{int} = 0.0498, R_{sigma} = 0.0258]	7818 [R_{int} = 0.0445, R_{sigma} = 0.0197]
Data/restraints/parameters	13316/0/667	7818/0/397
Goodness-of-fit on F^2	1.057	1.058
Final R indexes [I>=2σ (I)]	R_1 = 0.0270, wR_2 = 0.0588	R_1 = 0.0300, wR_2 = 0.0688
Final R indexes [all data]	R_1 = 0.0320, wR_2 = 0.0608	R_1 = 0.0342, wR_2 = 0.0709
Largest diff. peak/hole / e Å^{-3}	1.61/-1.54	0.70/-0.55
Diffractometer	Bruker APEX-II CCD	Bruker Apex CCD
Remarks	-	-
CCDC access code	2421710	2421711

Table S1. Crystallographic data and data collection parameters (continued)

	$[Tc(NS)Cl_3(CNAr^{Dipp2})_2]$ (**10**)	$[\{Tc(NS)Cl(CNAr^{Mes2})_2\}_2Cl_2]$ (**12**) x 2 CH_2Cl_2
Empirical formula	$C_{62}H_{74}Cl_3N_3STc$	$C_{102}H_{104}Cl_8N_6S_2Tc_2$
Formula weight	1097.65	1957.63
Temperature/K	200.00	104.00
Crystal system	monoclinic	triclinic
Space group	C2/c	$P\bar{1}$
a/Å	24.3880(14)	13.3612(12)
b/Å	24.3946(13)	14.3325(11)
c/Å	21.1855(13)	15.0922(13)
α/°	90	110.367(3)
β/°	108.191(5)	109.892(3)
γ/°	90	99.704(3)
Volume/Å^3	11974.1(12)	2409.0(4)
Z	8	1
ρ_{calc} / gcm^{-3}	1.218	1.349
μ / mm^{-1}	0.448	0.601
F(000)	4616.0	1012.0
Crystal size / mm^3	0.2 × 0.2 × 0.08	0.47 × 0.38 × 0.25
Radiation	MoKα (λ = 0.71073)	MoKα (λ = 0.71073)
2Θ range for data collection/°	9.526 to 52	4.58 to 48.992
Index ranges	$-30 \leq h \leq 29$, $-30 \leq k \leq 28$, $-26 \leq l \leq 26$	$-15 \leq h \leq 15$, $-16 \leq k \leq 16$, $-17 \leq l \leq 17$
Reflections collected	47952	44687
Independent reflections	11691 [R_{int} = 0.0974, R_{sigma} = 0.0797]	8002 [R_{int} = 0.0420, R_{sigma} = 0.0284]
Data/restraints/parameters	11691/0/679	8002/8/563
Goodness-of-fit on F^2	0.930	1.096
Final R indexes [I>=2σ (I)]	R_1 = 0.0468, wR_2 = 0.0781	R_1 = 0.0650, wR_2 = 0.1720
Final R indexes [all data]	R_1 = 0.0976, wR_2 = 0.0900	R_1 = 0.0744, wR_2 = 0.1877
Largest diff. peak/hole / e Å^{-3}	0.28/-0.54	1.97/-1.40
Diffractometer	STOE IPDS	Bruker Apex CCD
Remarks	-	-
CCDC access code	2421712	2421713

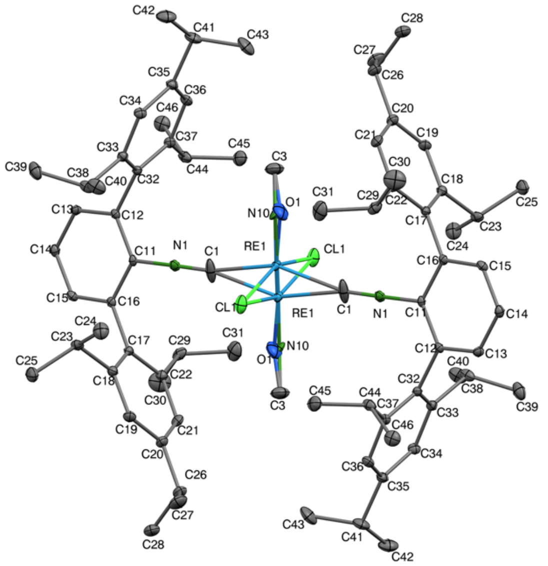

Figure S1. Ellipsoid representation of the structure of [ReNCl$_2$(CNAr^{Tripp2})$_2$(MeOH)] (**1**), also illustrating the disorder between two Re positions, and the N^{3-} and the methanol ligands in the axial positions. The thermal ellipsoids are set at a 30% probability level. Hydrogen atoms bonding to carbon atoms are omitted for clarity. b) Unit cell plot.

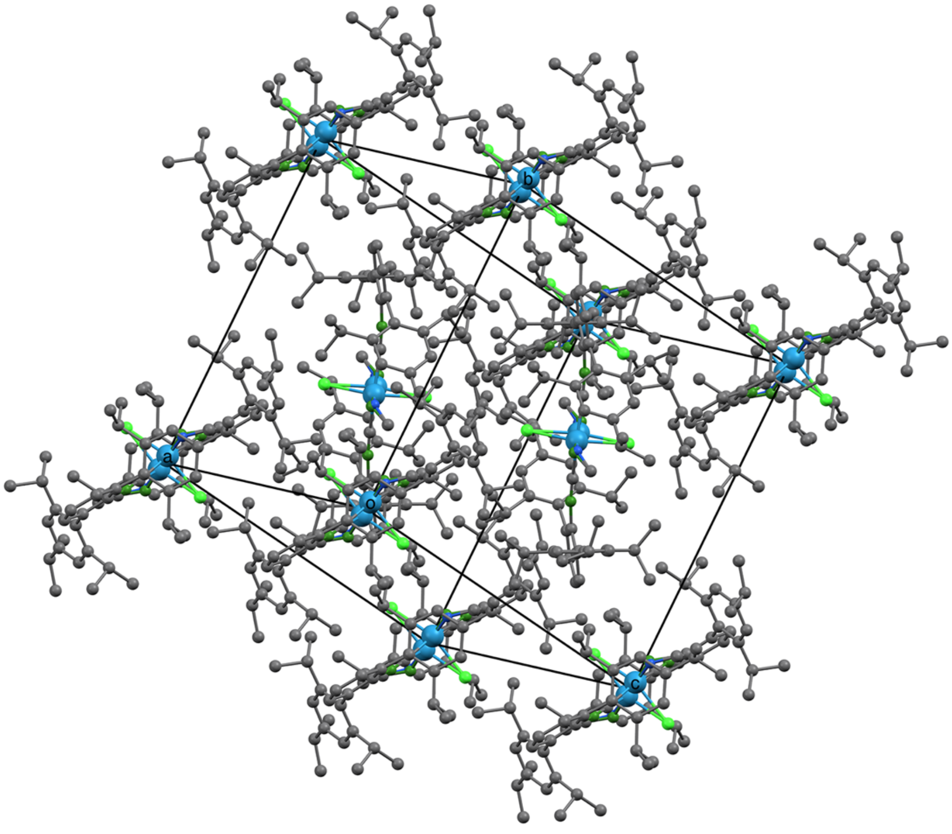

Figure S2. Unit cell plot.of [ReNCl$_2$(CNAr^{Tripp2})$_2$(MeOH)] (**1**).

Table S2. Bond lengths (Å) in $[ReNCl_2(CNAr^{Tripp2})_2(MeOH)]$ (**1**).

Re1	Re1[1]	0.9367(3)	C21	C22	1.391(2)
Re1	Cl1	2.3633(5)	C22	C29	1.523(2)
Re1	Cl1[1]	2.4262(5)	C23	C24	1.528(3)
Re1	N10[1]	2.476(13)	C23	C25	1.524(3)
Re1	N10	1.540(13)	C26	C27	1.528(3)
Re1	C1	2.098(2)	C26	C28	1.532(3)
Re1	C1[1]	2.131(2)	C29	C30	1.523(3)
Re1	O1	2.506(15)	C29	C31	1.529(3)
N1	C1	1.141(2)	C32	C33	1.403(2)
N1	C11	1.401(2)	C32	C37	1.400(2)
C11	C12	1.399(2)	C33	C34	1.398(2)
C11	C16	1.401(2)	C33	C38	1.525(2)
C12	C13	1.391(2)	C34	C35	1.391(2)
C12	C32	1.493(2)	C35	C36	1.393(2)
C13	C14	1.389(2)	C35	C41	1.525(2)
C14	C15	1.386(2)	C36	C37	1.387(2)
C15	C16	1.392(2)	C37	C44	1.524(2)
C16	C17	1.492(2)	C38	C39	1.532(3)
C17	C18	1.404(2)	C38	C40	1.533(3)
C17	C22	1.405(2)	C41	C42	1.515(3)
C18	C19	1.399(2)	C41	C43	1.526(3)
C18	C23	1.524(2)	C44	C45	1.531(3)
C19	C20	1.385(3)	C44	C46	1.528(3)
C20	C21	1.396(3)	O1	C3	1.309(15)
C20	C26	1.525(2)			

[1]2-x,-y,2-z

Table S3. Bond angles (°) in $[ReNCl_2(CNAr^{Tripp2})_2(MeOH)]$ (**1**).

Re1[1]	Re1	Cl1[1]	74.97(2)	C18	C17	C22	120.46(15)
Re1[1]	Re1	Cl1	82.52(2)	C22	C17	C16	120.12(15)
Re1[1]	Re1	N10	177.5(6)	C17	C18	C23	121.89(15)
Re1[1]	Re1	N10[1]	1.5(4)	C19	C18	C17	118.83(16)
Re1[1]	Re1	C1	79.19(8)	C19	C18	C23	119.27(15)
Re1[1]	Re1	C1[1]	75.23(8)	C20	C19	C18	121.89(17)
Re1[1]	Re1	O1	5.0(4)	C19	C20	C21	118.02(16)
Cl1	Re1	Cl1[1]	157.495(7)	C19	C20	C26	122.31(17)
Cl1[1]	Re1	N10[1]	75.4(4)	C21	C20	C26	119.65(16)
Cl1	Re1	N10[1]	82.1(4)	C22	C21	C20	122.31(17)
Cl1	Re1	O1	79.3(4)	C17	C22	C29	121.03(15)
Cl1[1]	Re1	O1	78.3(4)	C21	C22	C17	118.49(16)
N10	Re1	Cl1	98.1(6)	C21	C22	C29	120.47(16)
N10	Re1	Cl1[1]	104.3(6)	C18	C23	C24	111.36(15)
N10	Re1	N10[1]	179.1(3)	C18	C23	C25	111.88(15)
N10	Re1	C1	103.2(6)	C25	C23	C24	110.64(16)
N10	Re1	C1[1]	102.4(6)	C20	C26	C27	113.85(16)
N10	Re1	O1	177.0(11)	C20	C26	C28	110.35(16)
N10[1]	Re1	O1	3.7(8)	C27	C26	C28	110.23(18)
C1[1]	Re1	Cl1	86.97(5)	C22	C29	C30	110.52(17)
C1[1]	Re1	Cl1[1]	87.31(6)	C22	C29	C31	112.26(16)
C1	Re1	Cl1[1]	86.09(6)	C30	C29	C31	110.93(18)
C1	Re1	Cl1	89.75(6)	C33	C32	C12	119.36(14)
C1[1]	Re1	N10[1]	76.7(4)	C37	C32	C12	119.49(14)
C1	Re1	N10[1]	77.7(4)	C37	C32	C33	121.03(15)

C1	Re1	C1[1]	154.42(3)	C32	C33	C38	120.75(15)
C1	Re1	O1	75.4(4)	C34	C33	C32	118.47(15)
C1[1]	Re1	O1	79.0(4)	C34	C33	C38	120.73(15)
Re1	Cl1	Re1[1]	22.507(8)	C35	C34	C33	121.66(16)
C1	N1	C11	173.72(19)	C34	C35	C36	118.17(15)
Re1	N10	Re1[1]	0.9(2)	C34	C35	C41	123.36(16)
Re1	C1	Re1[1]	25.58(2)	C36	C35	C41	118.44(16)
N1	C1	Re1[1]	165.6(2)	C37	C36	C35	122.28(16)
N1	C1	Re1	168.5(2)	C32	C37	C44	121.63(15)
N1	C11	C16	118.07(14)	C36	C37	C32	118.37(16)
C12	C11	N1	118.82(14)	C36	C37	C44	119.96(16)
C12	C11	C16	123.10(14)	C33	C38	C39	110.17(16)
C11	C12	C32	122.17(14)	C33	C38	C40	113.33(15)
C13	C12	C11	117.44(14)	C39	C38	C40	111.16(16)
C13	C12	C32	120.39(14)	C35	C41	C43	108.95(17)
C14	C13	C12	120.88(15)	C42	C41	C35	114.65(17)
C15	C14	C13	120.18(15)	C42	C41	C43	110.62(18)
C14	C15	C16	121.24(15)	C37	C44	C45	112.39(16)
C11	C16	C17	120.94(14)	C37	C44	C46	110.32(15)
C15	C16	C11	117.02(15)	C46	C44	C45	111.34(16)
C15	C16	C17	122.01(14)	C3	O1	Re1	136.3(10)
C18	C17	C16	119.41(15)				

[1]2-x,-y,2-z

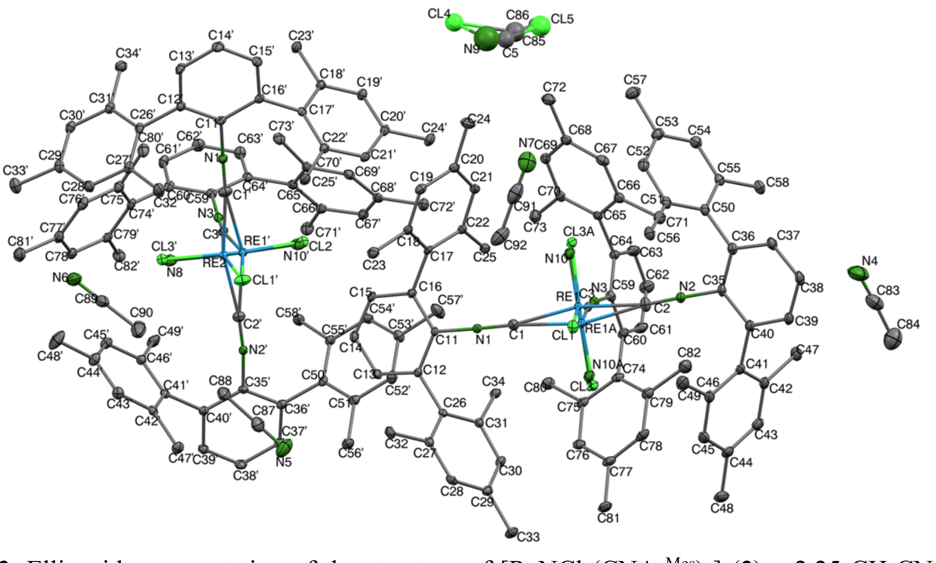

Figure S3. Ellipsoid representation of the structure of [$ReNCl_2(CNAr^{Mes})_3$] (**3**) x 2.25 CH_3CN x 0.25 CH_2Cl_2, illustrating both crstallographically independent molecules of the complex. Also note the disorders of the atoms in the axial positions of both complex molecules and the disorder of one of the solvent acetonitrile molecules with CH_2Cl_2. The thermal ellipsoids are set at a 30% probability level. Hydrogen atoms are omitted for clarity.

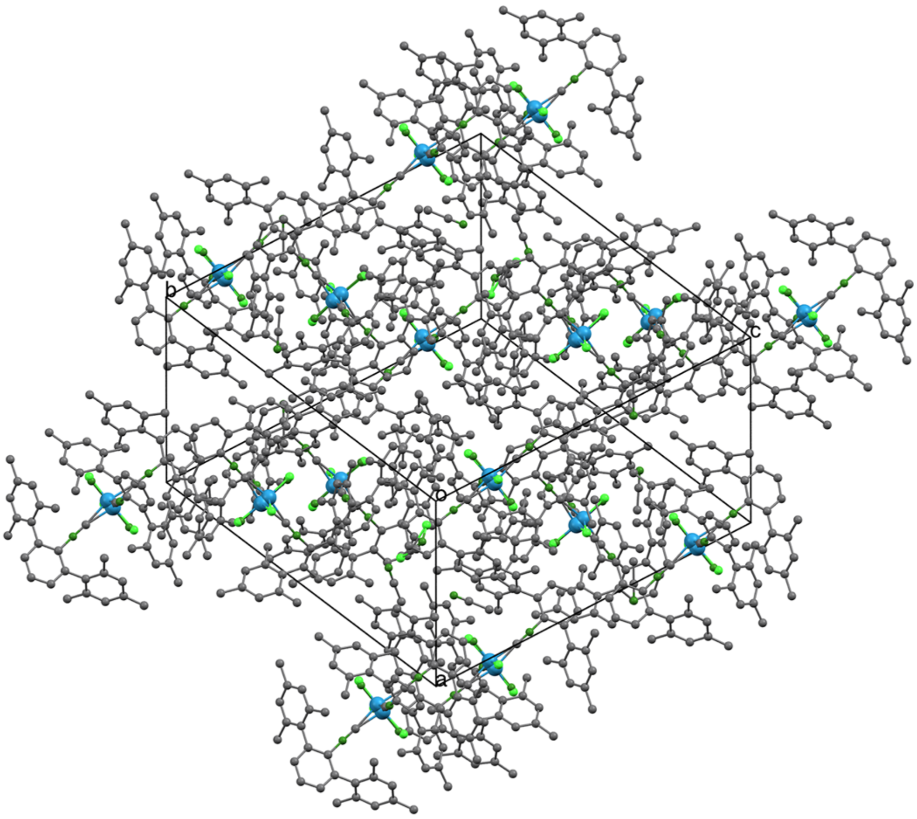

Figure S4. Unit cell plot of $[ReNCl_2(CNAr^{Mes})_3]$ (**3**) x 2.25 CH_3CN x 0.25 CH_2Cl_2.

Table S4. Bond lengths (Å) in $[ReNCl_2(CNAr^{Mes})_3]$ (**3**) x 2.25 CH_3CN x 0.25 CH_2Cl_2.

C17	C22	1.398(4)	C51	C50	1.405(4)
C17	C16	1.493(4)	C51	C52	1.392(4)
C17	C18	1.407(4)	C51	C56	1.503(4)
C2	N2	1.140(4)	C50	C36	1.500(4)
C2	Re1A	2.099(3)	C50	C55	1.403(4)
C2	Re1	2.113(3)	C36	C35	1.396(4)
N2	C35	1.406(4)	C36	C37	1.392(4)
N3	C3	1.136(4)	C41'	C46'	1.396(4)
N3	C59	1.407(3)	C31	C30	1.390(4)
C3	Re1A	2.049(3)	C31	C34	1.508(4)
C3	Re1	2.071(3)	C75	C76	1.392(4)
Cl1	Re1A	2.4408(15)	C75	C80	1.509(4)
Cl1	Re1	2.4359(16)	C28'	C29'	1.385(4)
Cl1'	Re1'	2.4302(17)	C77	C76	1.395(4)
Cl1'	Re2	2.4544(17)	C77	C78	1.382(5)
N10'	Re1'	1.76(2)	C77	C81	1.506(4)
Cl3A	Re1A	2.532(4)	C46'	C45'	1.388(4)
N10A	Re1A	1.674(11)	C46'	C49'	1.510(4)
Cl3'	Re1'	2.522(5)	C79	C78	1.388(4)
N1	C1	1.142(4)	C79	C82	1.512(5)
N1	C11	1.408(3)	C60	C61	1.387(4)
N2'	C2'	1.138(3)	C38'	C39'	1.385(4)
N2'	C35'	1.403(3)	C29'	C30'	1.388(4)
N1'	C11'	1.400(3)	C29'	C33'	1.509(4)
N1'	C1'	1.134(4)	C16'	C17'	1.492(4)
N3'	C3'	1.145(3)	C16'	C15'	1.396(4)

N3'	C59'	1.406(3)	C65	C66	1.396(4)
C3'	Re1'	2.088(3)	C65	C70	1.407(4)
C3'	Re2	2.044(3)	C82'	C79'	1.506(4)
C59	C64	1.399(4)	C19	C20	1.388(4)
C59	C60	1.398(4)	C30	C29	1.388(4)
C1	Re1A	2.115(3)	C43'	C44'	1.389(4)
C1	Re1	2.088(3)	C68'	C67'	1.392(4)
C11'	C12'	1.400(4)	C68'	C72'	1.507(4)
C11'	C16'	1.399(4)	C66	C71	1.508(4)
C70'	C65'	1.405(4)	C66	C67	1.390(4)
C70'	C69'	1.389(4)	C53'	C54'	1.385(4)
C70'	C73'	1.507(4)	C53'	C52'	1.386(4)
C2'	Re1'	2.111(3)	C53'	C57'	1.498(4)
C2'	Re2	2.094(3)	C79'	C78'	1.390(4)
C65'	C64'	1.489(4)	C51'	C52'	1.383(4)
C65'	C66'	1.406(4)	C51'	C56'	1.506(4)
C64	C65	1.489(4)	C41	C42	1.403(4)
C64	C63	1.397(4)	C41	C46	1.396(4)
C22	C25	1.508(4)	C17'	C18'	1.403(4)
C22	C21	1.390(4)	C15'	C14'	1.382(4)
C11	C16	1.403(4)	N6	C89	1.127(5)
C11	C12	1.400(4)	C78'	C77'	1.388(4)
C42'	C41'	1.400(4)	C19'	C20'	1.395(4)
C42'	C47'	1.508(4)	C19'	C18'	1.387(4)
C42'	C43'	1.385(4)	C13	C14	1.391(4)
C16	C15	1.393(4)	C37	C38	1.384(4)
C18	C23	1.505(4)	C63	C62	1.387(4)
C18	C19	1.387(4)	C70	C73	1.509(4)
C40'	C35'	1.395(4)	C70	C69	1.386(4)
C40'	C41'	1.489(4)	C21	C20	1.385(4)
C40'	C39'	1.394(4)	C20	C24	1.509(4)
C64'	C59'	1.402(4)	C23'	C18'	1.512(4)
C64'	C63'	1.392(4)	C29	C28	1.393(4)
C27'	C28'	1.396(4)	C29	C33	1.511(4)
C27'	C26'	1.394(4)	C63'	C62'	1.385(4)
C27'	C32'	1.512(4)	C75'	C80'	1.511(4)
C50'	C55'	1.401(4)	C75'	C76'	1.382(4)
C50'	C36'	1.488(4)	C58	C55	1.507(4)
C50'	C51'	1.407(4)	C61	C62	1.384(4)
C12	C26	1.494(4)	C52	C53	1.377(5)
C12	C13	1.393(4)	C61'	C62'	1.380(4)
C74'	C60'	1.490(4)	C44'	C45'	1.388(4)
C74'	C79'	1.397(4)	C44'	C48'	1.507(4)
C74'	C75'	1.403(4)	C21'	C20'	1.381(4)
C40	C35	1.403(4)	C77'	C76'	1.385(4)
C40	C41	1.496(4)	C77'	C81'	1.511(4)
C40	C39	1.393(4)	C68	C67	1.385(4)
C31'	C26'	1.402(4)	C68	C69	1.388(4)
C31'	C30'	1.385(4)	C68	C72	1.510(4)
C31'	C34'	1.508(4)	C38	C39	1.379(4)
C55'	C54'	1.389(4)	C54	C55	1.386(4)
C55'	C58'	1.512(4)	C54	C53	1.393(4)
C13'	C12'	1.396(4)	C20'	C24'	1.508(4)
C13'	C14'	1.386(4)	C42	C43	1.389(4)
C69'	C68'	1.382(4)	C42	C47	1.508(4)
C1'	Re1'	2.107(3)	C45	C44	1.383(5)
C1'	Re2	2.105(3)	C45	C46	1.393(5)
C15	C14	1.382(4)	C44	C43	1.387(5)

C26	C27	1.399(4)	C44	C48	1.509(4)
C26	C31	1.402(4)	C46	C49	1.507(5)
C35'	C36'	1.396(4)	N7	C91	1.078(6)
C60'	C59'	1.399(4)	C87	N5	1.131(5)
C60'	C61'	1.394(4)	C87	C88	1.459(5)
C37'	C36'	1.393(4)	C53	C57	1.511(4)
C37'	C38'	1.388(4)	C92	C91	1.463(7)
C12'	C26'	1.489(4)	C89	C90	1.447(5)
C22'	C17'	1.406(4)	N4	C83	1.149(6)
C22'	C25'	1.506(4)	C83	C84	1.412(6)
C22'	C21'	1.392(4)	Cl3	Re1	2.533(5)
C66'	C67'	1.388(4)	Re1	N10	1.667(13)
C66'	C71'	1.510(4)	Re2	N8	1.677(9)
C27	C32	1.512(4)	Re2	Cl2	2.530(4)
C27	C28	1.388(4)	Cl4	C5	1.714(9)
C74	C75	1.394(4)	Cl5	C5	1.745(9)
C74	C79	1.401(4)	C86	C85	1.489(14)
C74	C60	1.493(4)	C85	N9	1.416(17)

Table S5. Bond angles (°) in [ReNCl$_2$(CNAr^{Mes})$_3$] (**3**) x 2.25 CH_3CN x 0.25 CH_2Cl_2.

C22	C17	C16	121.9(2)	C69'	C68'	C67'	118.6(3)
C22	C17	C18	119.9(2)	C69'	C68'	C72'	120.5(3)
C18	C17	C16	118.2(2)	C67'	C68'	C72'	120.9(3)
N2	C2	Re1A	174.1(3)	C65	C66	C71	121.7(3)
N2	C2	Re1	169.6(3)	C67	C66	C65	118.9(3)
C2	N2	C35	168.9(3)	C67	C66	C71	119.4(3)
C3	N3	C59	168.7(3)	C54'	C53'	C52'	118.3(3)
N3	C3	Re1A	172.4(2)	C54'	C53'	C57'	121.3(3)
N3	C3	Re1	171.1(2)	C52'	C53'	C57'	120.3(3)
C1	N1	C11	167.0(3)	C74'	C79'	C82'	121.6(3)
C2'	N2'	C35'	169.7(3)	C78'	C79'	C74'	118.8(3)
C1'	N1'	C11'	172.0(3)	C78'	C79'	C82'	119.6(3)
C3'	N3'	C59'	170.2(3)	C50'	C51'	C56'	121.8(3)
N3'	C3'	Re1'	169.6(2)	C52'	C51'	C50'	118.8(3)
N3'	C3'	Re2	172.8(2)	C52'	C51'	C56'	119.3(3)
C64	C59	N3	118.7(2)	C42	C41	C40	118.1(3)
C60	C59	N3	118.1(2)	C46	C41	C40	121.6(3)
C60	C59	C64	123.2(2)	C46	C41	C42	120.3(3)
N1	C1	Re1A	174.1(2)	C53'	C54'	C55'	121.8(3)
N1	C1	Re1	170.3(2)	C51'	C52'	C53'	122.0(3)
N1'	C11'	C12'	118.8(2)	C31'	C30'	C29'	121.8(3)
C16'	C11'	N1'	118.0(2)	C22'	C17'	C16'	121.9(2)
C16'	C11'	C12'	123.2(2)	C18'	C17'	C22'	120.1(2)
C65'	C70'	C73'	123.1(2)	C18'	C17'	C16'	118.0(2)
C69'	C70'	C65'	118.7(3)	C14'	C15'	C16'	121.2(3)
C69'	C70'	C73'	118.1(3)	C77'	C78'	C79'	122.0(3)
N2'	C2'	Re1'	169.4(2)	C18'	C19'	C20'	121.8(3)
N2'	C2'	Re2	173.1(3)	C14	C13	C12	121.3(3)
C70'	C65'	C64'	120.3(2)	C38	C37	C36	121.3(3)
C70'	C65'	C66'	120.2(3)	C62	C63	C64	121.1(3)
C66'	C65'	C64'	119.5(2)	C65	C70	C73	121.0(3)
C59	C64	C65	123.3(2)	C69	C70	C65	119.1(3)
C63	C64	C59	116.8(3)	C69	C70	C73	119.8(3)
C63	C64	C65	119.9(2)	C15	C14	C13	120.0(3)
C17	C22	C25	123.1(2)	C20	C21	C22	122.2(3)
C21	C22	C17	118.8(3)	C19	C20	C24	120.9(3)

C21	C22	C25	118.1(2)	C21	C20	C19	118.1(3)
C16	C11	N1	118.1(2)	C21	C20	C24	121.0(3)
C12	C11	N1	118.4(2)	C30	C29	C28	117.9(2)
C12	C11	C16	123.4(2)	C30	C29	C33	120.4(3)
C41'	C42'	C47'	120.7(2)	C28	C29	C33	121.8(3)
C43'	C42'	C41'	118.9(2)	C62'	C63'	C64'	121.2(3)
C43'	C42'	C47'	120.4(2)	C38'	C39'	C40'	120.7(3)
C11	C16	C17	122.8(2)	C74'	C75'	C80'	121.4(3)
C15	C16	C17	120.2(2)	C76'	C75'	C74'	119.2(3)
C15	C16	C11	116.9(2)	C76'	C75'	C80'	119.4(3)
C17	C18	C23	121.2(2)	C62	C61	C60	120.7(3)
C19	C18	C17	119.2(3)	C75	C76	C77	121.5(3)
C19	C18	C23	119.5(3)	C53	C52	C51	122.4(3)
C35'	C40'	C41'	120.1(2)	C27	C28	C29	121.9(3)
C39'	C40'	C35'	116.9(2)	C62'	C61'	C60'	121.1(3)
C39'	C40'	C41'	123.0(2)	C61	C62	C63	120.5(3)
C59'	C64'	C65'	122.8(2)	C43'	C44'	C48'	121.1(3)
C63'	C64'	C65'	120.1(2)	C45'	C44'	C43'	118.5(3)
C63'	C64'	C59'	117.0(3)	C45'	C44'	C48'	120.4(3)
C28'	C27'	C32'	118.9(3)	C15'	C14'	C13'	120.3(3)
C26'	C27'	C28'	119.0(3)	C20'	C21'	C22'	121.8(3)
C26'	C27'	C32'	122.1(3)	C78'	C77'	C81'	120.6(3)
C55'	C50'	C36'	121.0(2)	C76'	C77'	C78'	118.1(3)
C55'	C50'	C51'	120.0(3)	C76'	C77'	C81'	121.4(3)
C51'	C50'	C36'	119.0(2)	C67	C68	C69	118.3(3)
C11	C12	C26	122.7(2)	C67	C68	C72	122.0(3)
C13	C12	C11	116.8(2)	C69	C68	C72	119.7(3)
C13	C12	C26	120.4(2)	C39	C38	C37	120.2(3)
C79'	C74'	C60'	119.2(2)	C38	C39	C40	121.3(3)
C79'	C74'	C75'	120.0(3)	C55	C54	C53	121.7(3)
C75'	C74'	C60'	120.7(3)	C19'	C20'	C24'	120.8(3)
C35	C40	C41	123.4(2)	C21'	C20'	C19'	118.4(3)
C39	C40	C35	116.8(3)	C21'	C20'	C24'	120.8(3)
C39	C40	C41	119.8(3)	C50	C55	C58	121.6(3)
C26'	C31'	C34'	121.5(3)	C54	C55	C50	119.1(3)
C30'	C31'	C26'	119.2(3)	C54	C55	C58	119.3(3)
C30'	C31'	C34'	119.3(3)	C44'	C45'	C46'	121.5(3)
C50'	C55'	C58'	122.3(3)	C17'	C18'	C23'	121.4(2)
C54'	C55'	C50'	118.9(2)	C19'	C18'	C17'	118.9(2)
C54'	C55'	C58'	118.8(3)	C19'	C18'	C23'	119.7(3)
C14'	C13'	C12'	120.9(3)	C41	C42	C47	121.8(3)
C68'	C69'	C70'	122.0(3)	C43	C42	C41	119.0(3)
N1'	C1'	Re1'	169.1(3)	C43	C42	C47	119.1(3)
N1'	C1'	Re2	174.0(3)	C77	C78	C79	122.3(3)
C14	C15	C16	121.3(3)	C2	Re1A	Cl1	86.89(9)
C27	C26	C12	120.0(2)	C2	Re1A	Cl3A	79.99(10)
C27	C26	C31	119.9(2)	C2	Re1A	C1	163.55(12)
C31	C26	C12	120.1(2)	C3	Re1A	C2	90.01(11)
C40'	C35'	N2'	116.9(2)	C3	Re1A	Cl1	167.59(9)
C40'	C35'	C36'	124.0(2)	C3	Re1A	Cl3A	79.06(11)
C36'	C35'	N2'	119.1(2)	C3	Re1A	C1	91.68(11)
C59'	C60'	C74'	122.2(2)	Cl1	Re1A	Cl3A	88.57(9)
C61'	C60'	C74'	120.5(2)	N10A	Re1A	C2	99.9(4)
C61'	C60'	C59'	117.3(3)	N10A	Re1A	C3	92.7(4)
C38'	C37'	C36'	120.6(3)	N10A	Re1A	Cl1	99.7(4)
C11'	C12'	C26'	123.3(2)	N10A	Re1A	Cl3A	171.8(4)
C13'	C12'	C11'	117.2(2)	N10A	Re1A	C1	96.3(3)
C13'	C12'	C26'	119.4(2)	C1	Re1A	Cl1	87.97(9)

C64'	C59'	N3'	118.3(2)	C1	Re1A	Cl3A	84.27(11)
C60'	C59'	N3'	118.6(2)	C44	C45	C46	122.6(3)
C60'	C59'	C64'	123.0(3)	C66'	C67'	C68'	121.6(3)
C17'	C22'	C25'	122.6(2)	C68	C67	C66	122.0(3)
C21'	C22'	C17'	118.9(3)	C45	C44	C43	117.9(3)
C21'	C22'	C25'	118.4(2)	C45	C44	C48	121.8(3)
C65'	C66'	C71'	122.2(3)	C43	C44	C48	120.3(3)
C67'	C66'	C65'	118.9(3)	C41	C46	C49	122.0(3)
C67'	C66'	C71'	118.9(3)	C45	C46	C41	118.4(3)
C26	C27	C32	121.4(2)	C45	C46	C49	119.6(3)
C28	C27	C26	119.2(2)	C61'	C62'	C63'	120.3(3)
C28	C27	C32	119.4(2)	C75'	C76'	C77'	121.9(3)
C75	C74	C79	120.7(3)	C44	C43	C42	121.8(3)
C75	C74	C60	119.0(2)	N5	C87	C88	179.3(5)
C79	C74	C60	120.3(3)	C52	C53	C54	118.2(3)
C50	C51	C56	122.5(3)	C52	C53	C57	121.1(3)
C52	C51	C50	118.5(3)	C54	C53	C57	120.8(3)
C52	C51	C56	119.0(3)	C70	C69	C68	121.6(3)
C51	C50	C36	121.7(3)	N6	C89	C90	179.3(6)
C55	C50	C51	120.1(3)	N7	C91	C92	179.2(5)
C55	C50	C36	118.2(2)	N4	C83	C84	177.3(5)
C35	C36	C50	123.8(2)	Cl1'	Re1'	Cl3'	83.66(10)
C37	C36	C50	119.3(3)	N10'	Re1'	Cl1'	102.6(8)
C37	C36	C35	116.9(3)	N10'	Re1'	Cl3'	173.6(8)
C42'	C41'	C40'	119.2(2)	N10'	Re1'	C3'	94.8(8)
C46'	C41'	C42'	120.4(2)	N10'	Re1'	C2'	97.8(8)
C46'	C41'	C40'	120.3(2)	N10'	Re1'	C1'	100.8(8)
C35'	C36'	C50'	121.3(2)	C3'	Re1'	Cl1'	162.57(11)
C37'	C36'	C50'	121.7(2)	C3'	Re1'	Cl3'	78.95(13)
C37'	C36'	C35'	116.9(2)	C3'	Re1'	C2'	89.06(12)
C26	C31	C34	122.8(2)	C3'	Re1'	C1'	89.89(11)
C30	C31	C26	119.1(2)	C2'	Re1'	Cl1'	87.93(9)
C30	C31	C34	118.1(2)	C2'	Re1'	Cl3'	83.43(14)
C74	C75	C80	120.7(3)	C1'	Re1'	Cl1'	87.52(9)
C76	C75	C74	118.8(3)	C1'	Re1'	Cl3'	78.11(14)
C76	C75	C80	120.5(3)	C1'	Re1'	C2'	161.37(14)
C29'	C28'	C27'	121.8(3)	C2	Re1	Cl1	86.70(9)
C76	C77	C81	121.1(3)	C2	Re1	Cl3	82.48(12)
C78	C77	C76	118.1(3)	C3	Re1	C2	89.03(11)
C78	C77	C81	120.8(3)	C3	Re1	Cl1	164.17(10)
C41'	C46'	C49'	121.1(2)	C3	Re1	C1	91.84(11)
C45'	C46'	C41'	119.0(3)	C3	Re1	Cl3	78.05(12)
C45'	C46'	C49'	119.9(3)	Cl1	Re1	Cl3	86.28(11)
C74	C79	C82	121.7(3)	C1	Re1	C2	166.04(13)
C78	C79	C74	118.4(3)	C1	Re1	Cl1	88.71(9)
C78	C79	C82	119.9(3)	C1	Re1	Cl3	84.07(12)
C59	C60	C74	121.8(2)	N10	Re1	C2	96.8(4)
C61	C60	C59	117.8(3)	N10	Re1	C3	94.5(4)
C61	C60	C74	120.5(2)	N10	Re1	Cl1	101.1(4)
C39'	C38'	C37'	120.9(3)	N10	Re1	C1	97.0(4)
C40	C35	N2	118.1(2)	N10	Re1	Cl3	172.5(4)
C36	C35	N2	118.5(2)	Cl1'	Re2	Cl2	85.83(10)
C36	C35	C40	123.4(3)	C3'	Re2	Cl1'	166.24(9)
C28'	C29'	C30'	118.2(3)	C3'	Re2	C2'	90.72(11)
C28'	C29'	C33'	121.6(3)	C3'	Re2	C1'	91.16(11)
C30'	C29'	C33'	120.1(3)	C3'	Re2	Cl2	80.41(12)
C11'	C16'	C17'	123.9(2)	C2'	Re2	Cl1'	87.67(9)
C15'	C16'	C11'	117.1(2)	C2'	Re2	C1'	164.83(12)

C15'	C16'	C17'	119.0(2)	C2'	Re2	Cl2	83.22(12)
C66	C65	C64	121.9(2)	C1'	Re2	Cl1'	86.93(9)
C66	C65	C70	120.0(3)	C1'	Re2	Cl2	82.26(12)
C70	C65	C64	118.0(2)	N8	Re2	Cl1'	103.1(3)
C27'	C26'	C31'	120.1(3)	N8	Re2	C3'	90.7(2)
C27'	C26'	C12'	121.0(2)	N8	Re2	C2'	97.5(3)
C31'	C26'	C12'	118.9(3)	N8	Re2	C1'	97.5(3)
C18	C19	C20	121.7(3)	N8	Re2	Cl2	171.1(3)
C29	C30	C31	122.0(3)	Cl4	C5	Cl5	111.1(6)
C42'	C43'	C44'	121.6(3)	N9	C85	C86	165.9(11)

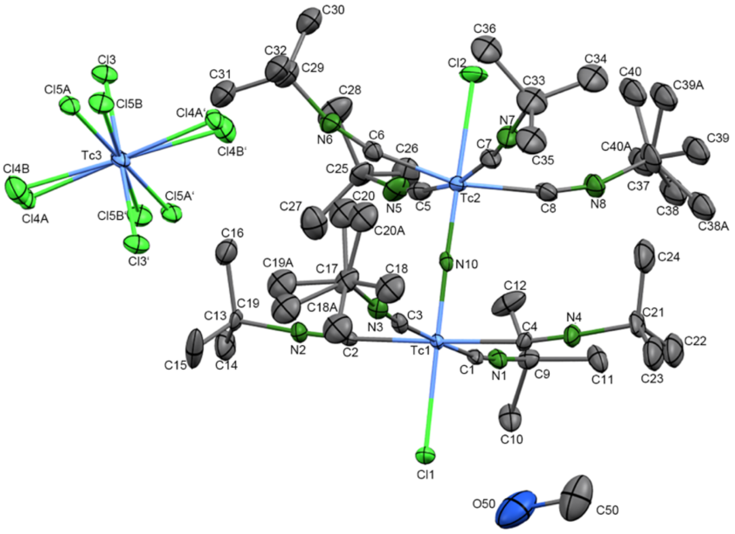

Figure S5. Ellipsoid representation of the structure of [{TcCl(CN^{t}Bu)$_4$}$_2$(μ-N)][TcCl$_6$] (**5**) x 2 MeOH. Also note the disorders of the atoms in the [TcCl$_6$]$^{2-}$ anion and in some of the *tert.*butyl groups. The thermal ellipsoids are set at a 30% probability level. Hydrogen atoms are omitted for clarity.

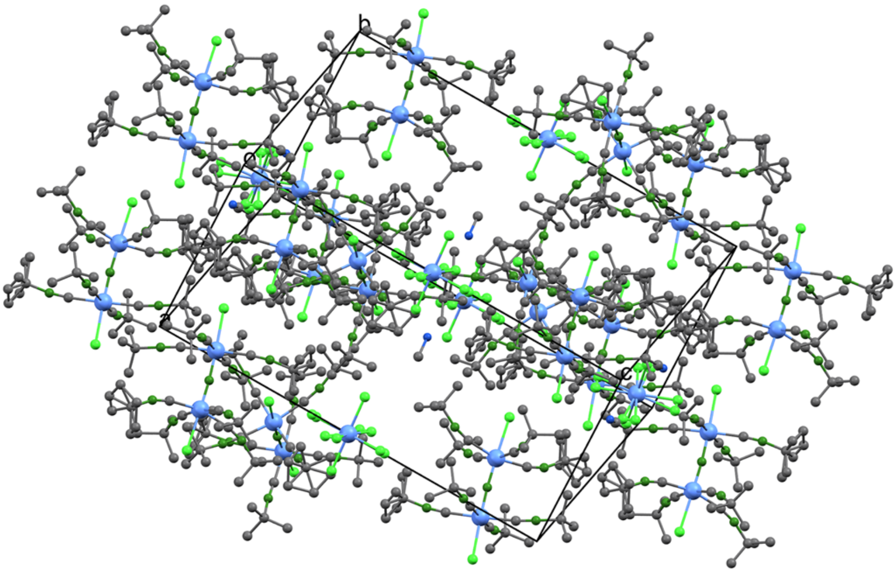

Figure S6. Unit cell plot of [{TcCl(CN^{t}Bu)$_4$}$_2$(μ-N)][TcCl$_6$] (**5**) x 2 MeOH.

Table S6. Bond lengths (Å) in [{TcCl(CN^{t}Bu)$_4$}$_2$(μ-N)][$TcCl_6$] (**5**) x 2 MeOH.

Tc1	Cl1	2.5287(19)	N4	C4	1.133(9)
Tc1	N10	1.784(6)	N4	C21	1.477(9)
Tc1	C1	2.084(6)	N7	C33	1.449(11)
Tc1	C3	2.075(6)	N7	C7	1.169(10)
Tc1	C4	2.065(7)	C10	C9	1.526(11)
Tc1	C2	2.062(7)	C33	C34	1.462(18)
Tc2	Cl2	2.502(2)	C33	C36	1.516(19)
Tc2	N10	1.770(6)	C33	C35	1.489(19)
Tc2	C6	2.062(8)	C29	C31	1.555(17)
Tc2	C8	2.072(8)	C29	C32	1.465(16)
Tc2	C7	2.071(8)	C29	C30	1.481(17)
Tc2	C5	2.074(8)	C21	C24	1.499(17)
Tc3	Cl3[1]	2.364(2)	C21	C22	1.470(15)
Tc3	Cl3	2.364(2)	C21	C23	1.519(17)
Tc3	Cl4A[1]	2.348(3)	C9	C12	1.502(13)
Tc3	Cl4A	2.348(3)	C9	C11	1.525(11)
Tc3	Cl5A[1]	2.373(3)	N5	C5	1.164(11)
Tc3	Cl5A	2.373(3)	N5	C25	1.469(11)
Tc3	Cl4B[1]	2.350(9)	O50	C50	1.464(18)
Tc3	Cl4B	2.350(9)	C37	C38	1.467(13)
Tc3	Cl5B[1]	2.365(7)	C37	C39	1.518(14)
Tc3	Cl5B	2.365(7)	C37	C40	1.531(14)
N1	C1	1.165(8)	C37	C40A	1.520(17)
N1	C9	1.459(8)	C37	C38A	1.520(17)
N3	C3	1.148(9)	C37	C39A	1.478(16)
N3	C17	1.469(9)	C25	C26	1.522(12)
N2	C13	1.456(9)	C25	C28	1.501(17)
N2	C2	1.143(9)	C25	C27	1.506(15)
N6	C6	1.138(11)	C17	C18	1.520(13)
N6	C29	1.464(14)	C17	C19	1.474(13)
N8	C8	1.135(10)	C17	C20	1.508(13)
N8	C37	1.480(9)	C17	C18A	1.510(17)
C13	C14	1.506(11)	C17	C19A	1.515(17)
C13	C16	1.513(13)	C17	C20A	1.482(17)
C13	C15	1.513(11)			

[1]1-x,1-y,-z

Table S7. Bond angles (°) in [{TcCl(CN^{t}Bu)$_4$}$_2$(μ-N)][$TcCl_6$] (**5**) x 2 MeOH

N10	Tc1	Cl1	177.53(15)	C14	C13	C15	113.7(8)
N10	Tc1	C1	87.6(2)	C15	C13	C16	106.7(8)
N10	Tc1	C3	90.7(3)	C4	N4	C21	170.0(8)
N10	Tc1	C4	88.2(3)	C7	N7	C33	170.6(11)
N10	Tc1	C2	90.5(3)	N6	C6	Tc2	167.3(7)
C1	Tc1	Cl1	90.0(2)	N8	C8	Tc2	168.0(8)
C3	Tc1	Cl1	91.7(2)	N7	C33	C34	108.1(9)
C3	Tc1	C1	178.3(3)	N7	C33	C36	106.7(9)
C4	Tc1	Cl1	92.3(2)	N7	C33	C35	107.8(10)
C4	Tc1	C1	89.6(3)	C34	C33	C36	112.1(11)
C4	Tc1	C3	90.3(3)	C34	C33	C35	114.9(11)
C2	Tc1	Cl1	89.04(19)	C35	C33	C36	106.9(11)
C2	Tc1	C1	93.0(2)	N4	C4	Tc1	173.3(7)
C2	Tc1	C3	87.1(2)	N2	C2	Tc1	174.0(6)

C2	Tc1	C4	177.1(3)	N7	C7	Tc2	171.2(8)
N10	Tc2	Cl2	178.82(17)	N6	C29	C31	108.0(9)
N10	Tc2	C6	98.0(3)	N6	C29	C32	108.1(10)
N10	Tc2	C8	97.4(3)	N6	C29	C30	105.0(9)
N10	Tc2	C7	94.9(3)	C32	C29	C31	106.8(9)
N10	Tc2	C5	95.2(3)	C32	C29	C30	120.1(11)
C6	Tc2	Cl2	82.6(2)	C30	C29	C31	108.4(11)
C6	Tc2	C8	164.6(3)	N4	C21	C24	107.1(8)
C6	Tc2	C7	88.0(3)	N4	C21	C23	109.8(8)
C6	Tc2	C5	86.3(3)	C24	C21	C23	107.8(9)
C8	Tc2	Cl2	82.1(2)	C22	C21	N4	106.1(7)
C8	Tc2	C5	92.7(3)	C22	C21	C24	114.3(10)
C7	Tc2	Cl2	86.2(3)	C22	C21	C23	111.6(11)
C7	Tc2	C8	90.4(3)	N1	C9	C10	107.3(6)
C7	Tc2	C5	169.0(4)	N1	C9	C12	108.8(7)
C5	Tc2	Cl2	83.8(2)	N1	C9	C11	107.3(6)
Cl3[1]	Tc3	Cl3	180.0	C12	C9	C10	111.2(8)
Cl3[1]	Tc3	Cl5A[1]	90.85(11)	C12	C9	C11	111.8(8)
Cl3[1]	Tc3	Cl5A	89.15(11)	C11	C9	C10	110.2(7)
Cl3	Tc3	Cl5A	90.85(11)	C5	N5	C25	168.5(10)
Cl3	Tc3	Cl5A[1]	89.15(11)	N5	C5	Tc2	172.3(8)
Cl3	Tc3	Cl5B[1]	94.0(3)	N8	C37	C39	107.3(8)
Cl3[1]	Tc3	Cl5B[1]	86.0(3)	N8	C37	C40	104.5(8)
Cl3[1]	Tc3	Cl5B	94.0(3)	N8	C37	C40A	104.4(13)
Cl3	Tc3	Cl5B	86.0(3)	N8	C37	C38A	107.8(13)
Cl4A	Tc3	Cl3	88.24(12)	C38	C37	N8	112.1(9)
Cl4A[1]	Tc3	Cl3	91.76(12)	C38	C37	C39	116.6(13)
Cl4A[1]	Tc3	Cl4A	180.0	C38	C37	C40	112.4(13)
Cl4A	Tc3	Cl5A	89.3(3)	C39	C37	C40	102.9(12)
Cl4A[1]	Tc3	Cl5A	90.7(3)	C38A	C37	C40A	96(2)
Cl4A[1]	Tc3	Cl5B[1]	72.1(6)	C39A	C37	N8	112.7(13)
Cl4A	Tc3	Cl5B[1]	107.9(6)	C39A	C37	C40A	116(2)
Cl4B	Tc3	Cl3	93.5(3)	C39A	C37	C38A	118(2)
Cl4B[1]	Tc3	Cl3	86.5(3)	N5	C25	C26	108.8(8)
Cl4B	Tc3	Cl5B	89.4(10)	N5	C25	C28	104.7(8)
Cl4B[1]	Tc3	Cl5B[1]	89.4(10)	N5	C25	C27	108.4(8)
Cl4B[1]	Tc3	Cl5B	90.6(10)	C28	C25	C26	111.7(9)
Cl4B	Tc3	Cl5B[1]	90.6(10)	C28	C25	C27	113.8(10)
Cl5B	Tc3	Cl5B[1]	180.0	C27	C25	C26	109.2(9)
C1	N1	C9	176.7(6)	N3	C17	C18	105.7(8)
Tc2	N10	Tc1	175.7(3)	N3	C17	C19	112.2(8)
C3	N3	C17	170.8(8)	N3	C17	C20	105.2(8)
N1	C1	Tc1	172.9(6)	N3	C17	C18A	109.4(16)
N3	C3	Tc1	174.2(7)	N3	C17	C19A	96.9(15)
C2	N2	C13	175.7(7)	N3	C17	C20A	117.0(16)
C6	N6	C29	165.5(8)	C19	C17	C18	112.4(10)
C8	N8	C37	162.8(10)	C19	C17	C20	113.9(12)
N2	C13	C14	108.3(7)	C20	C17	C18	106.8(11)
N2	C13	C16	106.9(6)	C18A	C17	C19A	96(3)
N2	C13	C15	108.1(6)	C20A	C17	C18A	121(2)
C14	C13	C16	112.9(8)	C20A	C17	C19A	111(3)

[1]1-x,1-y,-z

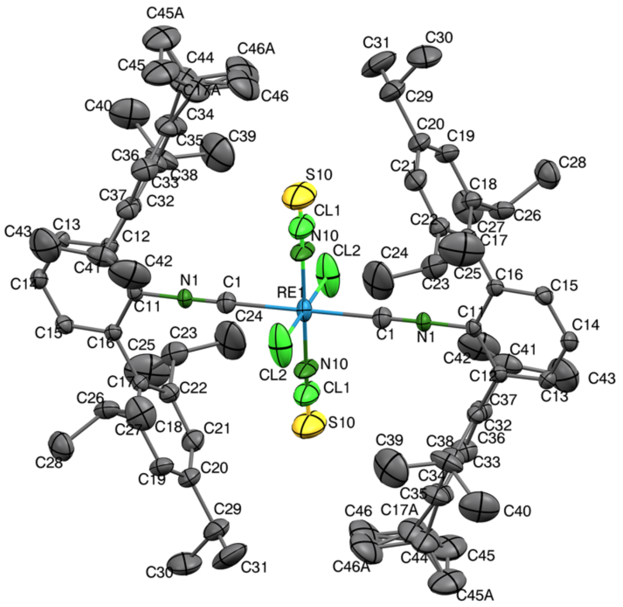

Figure S7. Ellipsoid representation of the structure of [Re(NS)Cl$_3$(CNAr^{Tripp2})$_2$] (**6**), also illustrating disorders of the ligands in axial positions and in some of the isopropyl groups. The thermal ellipsoids are set at a 30% probability level. Hydrogen atoms are omitted for clarity.

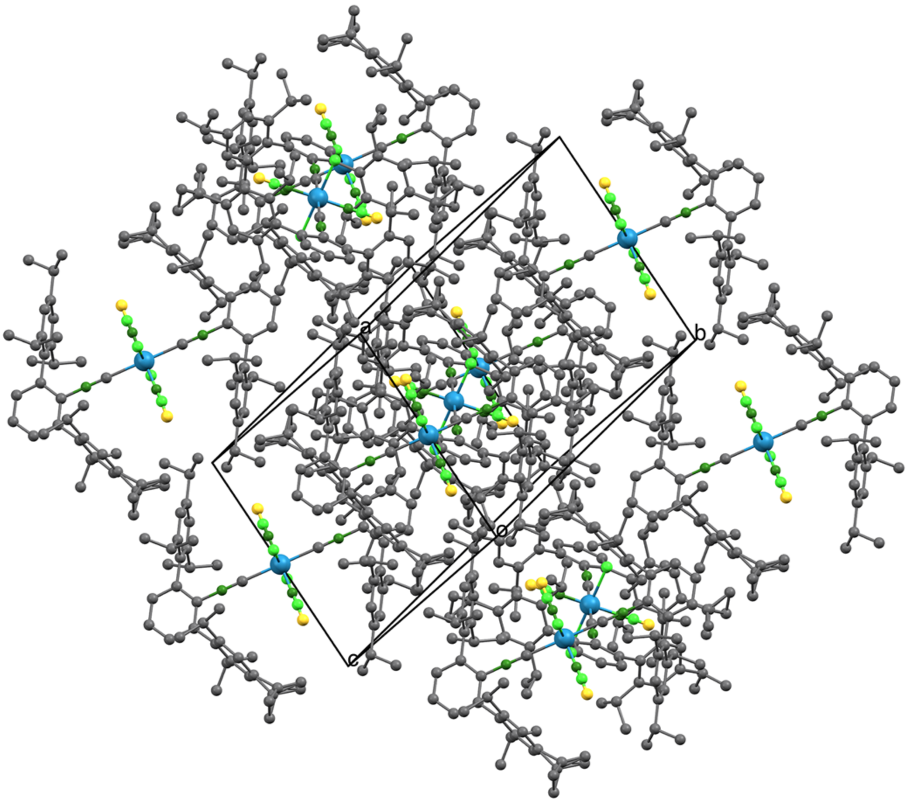

Figure S8. Unit cell plot of [Re(NS)Cl$_3$(CNAr^{Tripp2})$_2$] (**6**).

Table S8. Bond lengths (Å) in [Re(NS)Cl$_3$(CNAr^{Tripp2})$_2$] (**6**).

Re1	Cl2	2.3256(13)	C23	C24	1.525(8)
Re1	Cl2[1]	2.3257(13)	C23	C25	1.503(8)
Re1	C1[1]	2.110(4)	C26	C27	1.521(7)
Re1	C1	2.110(4)	C26	C28	1.528(7)
Re1	Cl1	2.386(5)	C29	C30	1.508(8)
Re1	Cl1[1]	2.386(5)	C29	C31	1.497(8)
Re1	N10	1.665(10)	C32	C33	1.384(5)
Re1	N10[1]	1.665(10)	C32	C37	1.391(5)
N1	C1	1.136(4)	C33	C34	1.386(6)
N1	C11	1.397(4)	C33	C38	1.531(6)
C11	C12	1.401(5)	C34	C35	1.357(7)
C11	C16	1.391(5)	C35	C36	1.385(7)
C12	C13	1.388(5)	C35	C44	1.540(10)
C12	C32	1.497(5)	C35	C17A	1.552(16)
C13	C14	1.381(5)	C36	C37	1.391(6)
C14	C15	1.381(5)	C37	C41	1.523(6)
C15	C16	1.388(5)	C38	C39	1.508(9)
C16	C17	1.498(5)	C38	C40	1.537(8)
C17	C18	1.400(5)	C41	C42	1.522(7)
C17	C22	1.402(5)	C41	C43	1.520(8)
C18	C19	1.401(6)	C44	C45	1.36(4)
C18	C26	1.519(6)	C44	C46	1.57(3)
C19	C20	1.354(6)	C17A	C45A	1.59(3)
C20	C21	1.384(6)	C17A	C46A	1.37(5)
C20	C29	1.539(6)	Cl1	S10	0.856(5)
C21	C22	1.394(6)	Cl1	N10	0.721(7)
C22	C23	1.511(6)	S10	N10	1.574(9)

[1]1-x,1-y,1-z

Table S9. Bond angles (°) in [Re(NS)Cl$_3$(CNAr^{Tripp2})$_2$] (**6**).

Cl2	Re1	Cl2[1]	180.0	C19	C20	C29	122.0(4)
Cl2[1]	Re1	Cl1[1]	90.23(14)	C21	C20	C29	119.7(5)
Cl2[1]	Re1	Cl1	89.77(14)	C20	C21	C22	122.7(5)
Cl2	Re1	Cl1[1]	89.77(14)	C17	C22	C23	121.9(4)
Cl2	Re1	Cl1	90.23(14)	C21	C22	C17	117.7(4)
C1	Re1	Cl2	89.47(11)	C21	C22	C23	120.3(4)
C1[1]	Re1	Cl2	90.52(11)	C22	C23	C24	112.4(5)
C1[1]	Re1	Cl2[1]	89.48(11)	C25	C23	C22	111.2(5)
C1	Re1	Cl2[1]	90.52(11)	C25	C23	C24	112.0(6)
C1	Re1	C1[1]	180.0	C18	C26	C27	111.9(4)
C1[1]	Re1	Cl1[1]	89.63(14)	C18	C26	C28	111.5(4)
C1	Re1	Cl1[1]	90.38(14)	C27	C26	C28	110.3(4)
C1[1]	Re1	Cl1	90.37(14)	C30	C29	C20	109.8(5)
C1	Re1	Cl1	89.63(14)	C31	C29	C20	114.0(5)
Cl1[1]	Re1	Cl1	180.0	C31	C29	C30	111.2(6)
N10[1]	Re1	Cl2[1]	89.4(5)	C33	C32	C12	120.4(3)
N10	Re1	Cl2[1]	90.6(5)	C33	C32	C37	120.4(3)
N10[1]	Re1	Cl2	90.6(5)	C37	C32	C12	119.1(3)
N10	Re1	Cl2	89.4(5)	C32	C33	C34	118.7(4)
N10[1]	Re1	C1[1]	89.2(4)	C32	C33	C38	120.7(4)
N10	Re1	C1[1]	90.8(4)	C34	C33	C38	120.6(4)
N10	Re1	C1	89.2(4)	C35	C34	C33	122.6(4)

N10[1]	Re1	C1	90.8(4)	C34	C35	C36	118.1(4)
N10[1]	Re1	Cl1[1]	0.9(5)	C34	C35	C44	119.0(15)
N10[1]	Re1	Cl1	179.1(5)	C34	C35	C17A	124(3)
N10	Re1	Cl1	0.9(5)	C36	C35	C44	122.7(15)
N10	Re1	Cl1[1]	179.1(5)	C36	C35	C17A	117(2)
N10[1]	Re1	N10	180.0	C35	C36	C37	121.6(4)
C1	N1	C11	176.0(4)	C32	C37	C36	118.6(4)
N1	C1	Re1	178.8(3)	C32	C37	C41	121.5(4)
N1	C11	C12	118.0(3)	C36	C37	C41	119.9(4)
C16	C11	N1	118.0(3)	C33	C38	C40	110.0(5)
C16	C11	C12	124.0(3)	C39	C38	C33	111.7(5)
C11	C12	C32	122.4(3)	C39	C38	C40	111.0(5)
C13	C12	C11	116.2(3)	C42	C41	C37	112.7(5)
C13	C12	C32	121.4(3)	C43	C41	C37	110.3(5)
C14	C13	C12	121.6(4)	C43	C41	C42	112.6(6)
C13	C14	C15	120.2(3)	C35	C44	C46	106.3(17)
C14	C15	C16	121.1(3)	C45	C44	C35	118(2)
C11	C16	C17	121.6(3)	C45	C44	C46	108.6(16)
C15	C16	C11	116.9(3)	C35	C17A	C45A	103(2)
C15	C16	C17	121.5(3)	C46A	C17A	C35	116(4)
C18	C17	C16	119.5(3)	C46A	C17A	C45A	101(3)
C18	C17	C22	120.4(3)	S10	Cl1	Re1	174.4(6)
C22	C17	C16	120.1(3)	N10	Cl1	Re1	2.1(12)
C17	C18	C19	118.5(4)	N10	Cl1	S10	172.4(13)
C17	C18	C26	121.9(3)	Cl1	S10	N10	3.5(6)
C19	C18	C26	119.6(3)	Cl1	N10	Re1	177.0(17)
C20	C19	C18	122.3(4)	Cl1	N10	S10	4.1(7)
C19	C20	C21	118.4(5)	S10	N10	Re1	178.6(8)

[1]1-x,1-y,1-z

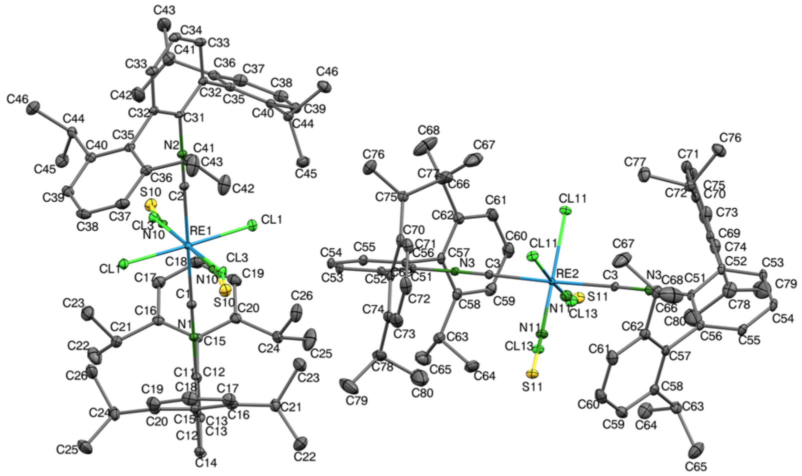

Figure S9. Ellipsoid representation of the structure of $[Re(NS)Cl_3(CNAr^{Dipp2})_2]$ (**7**), illustrating both crystallographically independent molecules of the complex. Also note the disorders of the atoms in the central coordination planes of both complex molecules. The thermal ellipsoids are set at a 30% probability level. Hydrogen atoms are omitted for clarity.

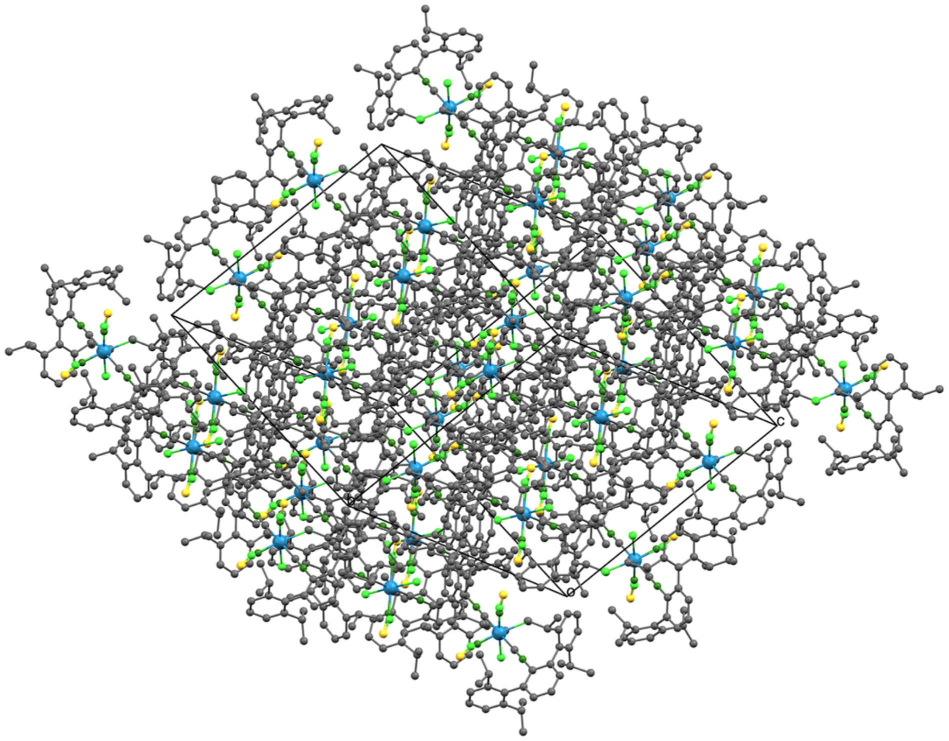

Figure S10. Unit cell plot of [Re(NS)Cl$_3$(CNAr$^{\mathrm{Dipp2}}$)$_2$] (**7**).

Table S10. Bond lengths (Å) in [Re(NS)Cl$_3$(CNAr$^{\mathrm{Dipp2}}$)$_2$] (**7**).

Re1	Cl2	2.3418(6)	C32	C35	1.495(3)
Re1	Cl2^{1}	2.3417(6)	C33	C34	1.385(3)
Re1	C1	2.096(3)	C35	C36	1.409(3)
Re1	C2	2.104(3)	C35	C40	1.405(3)
Re1	N10^{1}	1.871(8)	C36	C37	1.393(4)
Re1	N10	1.872(8)	C36	C41	1.525(4)
Re1	Cl1	2.3289(19)	C37	C38	1.381(4)
Re1	Cl1^{1}	2.3289(19)	C38	C39	1.384(4)
Re2	Cl12	2.3769(6)	C39	C40	1.395(4)
Re2	Cl12^{2}	2.3769(6)	C40	C44	1.527(4)
Re2	Cl11	2.3188(13)	C41	C42	1.517(4)
Re2	Cl11^{2}	2.3187(13)	C41	C43	1.524(4)
Re2	N11^{2}	1.811(5)	C44	C45	1.531(3)
Re2	N11	1.811(5)	C44	C46	1.527(4)
Re2	C3	2.100(2)	C51	C52	1.399(3)
Re2	C3^{2}	2.100(2)	C51	C56	1.402(3)
Cl11	S20	1.0554(19)	C52	C53	1.393(3)
Cl11	N11	0.510(4)	C52	C69	1.494(3)
S10	N10	1.521(8)	C53	C54	1.385(4)
S10	Cl1	1.115(3)	C54	C55	1.386(4)
S20	N11	1.532(5)	C55	C56	1.396(3)
N1	C1	1.154(4)	C56	C57	1.491(3)
N1	C11	1.397(4)	C57	C58	1.405(3)
N2	C2	1.144(4)	C57	C62	1.409(4)
N2	C31	1.406(4)	C58	C59	1.394(4)
N3	C3	1.145(3)	C58	C63	1.516(4)
N3	C51	1.403(3)	C59	C60	1.381(4)
C11	C12	1.407(3)	C60	C61	1.384(4)
C11	C12^{1}	1.407(3)	C61	C62	1.391(4)

C12	C13	1.390(3)	C62	C66	1.524(4)
C12	C15	1.496(4)	C63	C64	1.531(4)
C13	C14	1.388(3)	C63	C65	1.530(4)
C15	C16	1.403(4)	C66	C67	1.500(4)
C15	C20	1.410(4)	C66	C68	1.512(5)
C16	C17	1.393(4)	C69	C70	1.409(4)
C16	C21	1.521(4)	C69	C74	1.408(4)
C17	C18	1.383(4)	C70	C71	1.389(4)
C18	C19	1.380(5)	C70	C75	1.524(4)
C19	C20	1.392(4)	C71	C72	1.387(4)
C20	C24	1.521(4)	C72	C73	1.375(5)
C21	C22	1.524(4)	C73	C74	1.393(4)
C21	C23	1.527(4)	C74	C78	1.526(4)
C24	C25	1.523(4)	C75	C76	1.527(4)
C24	C26	1.524(4)	C75	C77	1.525(4)
C31	C32[1]	1.399(3)	C78	C79	1.511(5)
C31	C32	1.399(3)	C78	C80	1.508(5)
C32	C33	1.399(3)			

[1]1-x,+y,1/2-z; [2]-x,+y,1/2-z

Table S11. Bond angles (°) in [Re(NS)Cl_3(CNAr^{Dipp2})$_2$] (**7**).

Cl2[1]	Re1	Cl2	178.43(3)	C19	C20	C24	120.3(3)
C1	Re1	Cl2	90.788(15)	C16	C21	C22	111.2(3)
C1	Re1	Cl2[1]	90.786(15)	C16	C21	C23	111.7(2)
C1	Re1	C2	180.0	C22	C21	C23	110.9(3)
C1	Re1	Cl1	90.74(8)	C20	C24	C25	113.1(3)
C1	Re1	Cl1[1]	90.74(8)	C20	C24	C26	111.1(2)
C2	Re1	Cl2	89.212(15)	C25	C24	C26	110.2(3)
C2	Re1	Cl2[1]	89.214(15)	C32	C31	N2	118.03(14)
C2	Re1	Cl1[1]	89.26(8)	C32[1]	C31	N2	118.03(14)
C2	Re1	Cl1	89.26(8)	C32	C31	C32[1]	123.9(3)
N10[1]	Re1	Cl2	93.9(3)	C31	C32	C33	116.8(2)
N10	Re1	Cl2	86.2(3)	C31	C32	C35	121.7(2)
N10[1]	Re1	Cl2[1]	86.2(3)	C33	C32	C35	121.4(2)
N10	Re1	Cl2[1]	93.9(3)	C34	C33	C32	120.6(2)
N10	Re1	C1	88.9(3)	C33[1]	C34	C33	121.1(3)
N10[1]	Re1	C1	88.9(3)	C36	C35	C32	119.3(2)
N10	Re1	C2	91.1(3)	C40	C35	C32	119.2(2)
N10[1]	Re1	C2	91.1(3)	C40	C35	C36	121.5(2)
N10[1]	Re1	N10	177.9(6)	C35	C36	C41	120.9(2)
N10	Re1	Cl1[1]	177.2(4)	C37	C36	C35	117.7(2)
N10[1]	Re1	Cl1[1]	3.3(4)	C37	C36	C41	121.4(2)
Cl1	Re1	Cl2	88.90(8)	C38	C37	C36	121.6(3)
Cl1[1]	Re1	Cl2	91.08(8)	C37	C38	C39	120.1(3)
Cl1	Re1	Cl1[1]	178.51(16)	C38	C39	C40	120.7(3)
Cl12	Re2	Cl12[2]	87.59(3)	C35	C40	C44	122.0(2)
Cl11	Re2	Cl12	87.88(4)	C39	C40	C35	118.4(2)
Cl11[2]	Re2	Cl12	175.44(4)	C39	C40	C44	119.6(2)
Cl11[2]	Re2	Cl12[2]	87.88(4)	C42	C41	C36	111.5(2)
Cl11	Re2	Cl12[2]	175.44(4)	C42	C41	C43	110.0(3)
Cl11[2]	Re2	Cl11	96.66(7)	C43	C41	C36	114.0(3)
N11	Re2	Cl12	86.99(17)	C40	C44	C45	111.9(2)
N11[2]	Re2	Cl12	174.31(17)	C46	C44	C40	111.1(2)
N11[2]	Re2	Cl12[2]	86.99(17)	C46	C44	C45	110.6(2)

N11	Re2	Cl12^{2}	174.31(17)
N11	Re2	Cl11	1.5(2)
N11^{2}	Re2	Cl11	97.56(16)
N11	Re2	Cl11^{2}	97.56(16)
N11^{2}	Re2	Cl11^{2}	1.5(2)
N11	Re2	N11^{2}	98.5(3)
N11	Re2	C3	91.97(19)
N11^{2}	Re2	C3	89.44(19)
N11	Re2	C3^{2}	89.45(19)
N11^{2}	Re2	C3^{2}	91.97(19)
C3^{2}	Re2	Cl12^{2}	88.82(6)
C3	Re2	Cl12	88.82(6)
C3^{2}	Re2	Cl12	89.62(6)
C3	Re2	Cl12^{2}	89.62(6)
C3	Re2	Cl11	90.77(7)
C3	Re2	Cl11^{2}	90.67(7)
C3^{2}	Re2	Cl11	90.67(7)
C3^{2}	Re2	Cl11^{2}	90.77(7)
C3	Re2	C3^{2}	177.83(12)
S20	Cl11	Re2	158.93(13)
N11	Cl11	Re2	5.4(7)
N11	Cl11	S20	154.4(7)
Cl11	S20	N11	8.3(2)
C1	N1	C11	180.0
C2	N2	C31	180.0
C3	N3	C51	178.8(2)
Cl11	N11	Re2	173.1(9)
Cl11	N11	S20	17.3(5)
S20	N11	Re2	168.2(4)
N1	C1	Re1	180.0
N2	C2	Re1	180.0
N3	C3	Re2	178.6(2)
N1	C11	C12^{1}	118.45(15)
N1	C11	C12	118.45(15)
C12	C11	C12^{1}	123.1(3)
C11	C12	C15	120.3(2)
C13	C12	C11	117.1(2)
C13	C12	C15	122.6(2)
C14	C13	C12	121.2(2)
C13^{1}	C14	C13	120.4(3)
C16	C15	C12	119.5(2)
C16	C15	C20	121.7(2)
C20	C15	C12	118.8(2)
C15	C16	C21	122.3(2)
C17	C16	C15	118.1(3)
C17	C16	C21	119.7(3)
C18	C17	C16	120.8(3)
C19	C18	C17	120.6(3)
C18	C19	C20	120.9(3)
C15	C20	C24	121.7(2)
C19	C20	C15	118.0(3)
C52	C51	N3	118.3(2)
C52	C51	C56	123.7(2)
C56	C51	N3	118.0(2)
C51	C52	C69	121.7(2)
C53	C52	C51	116.9(2)
C53	C52	C69	121.4(2)
C54	C53	C52	121.1(2)
C53	C54	C55	120.5(2)
C54	C55	C56	121.0(2)
C51	C56	C57	120.8(2)
C55	C56	C51	116.8(2)
C55	C56	C57	122.4(2)
C58	C57	C56	119.9(2)
C58	C57	C62	120.9(2)
C62	C57	C56	119.1(2)
C57	C58	C63	122.0(2)
C59	C58	C57	118.4(2)
C59	C58	C63	119.6(2)
C60	C59	C58	120.9(3)
C59	C60	C61	120.5(3)
C60	C61	C62	120.5(3)
C57	C62	C66	121.5(2)
C61	C62	C57	118.7(2)
C61	C62	C66	119.8(2)
C58	C63	C64	110.9(2)
C58	C63	C65	111.6(3)
C65	C63	C64	110.6(3)
C67	C66	C62	112.0(3)
C67	C66	C68	110.6(3)
C68	C66	C62	112.7(3)
C70	C69	C52	119.4(2)
C74	C69	C52	119.2(2)
C74	C69	C70	121.3(2)
C69	C70	C75	122.1(2)
C71	C70	C69	118.4(3)
C71	C70	C75	119.5(2)
C72	C71	C70	120.8(3)
C73	C72	C71	120.1(3)
C72	C73	C74	121.7(3)
C69	C74	C78	121.0(3)
C73	C74	C69	117.7(3)
C73	C74	C78	121.2(3)
C70	C75	C76	111.6(2)
C70	C75	C77	111.8(2)
C77	C75	C76	109.6(2)
C79	C78	C74	114.2(3)
C80	C78	C74	111.6(3)
C80	C78	C79	110.7(3)
S10	N10	Re1	169.5(7)
S10	Cl1	Re1	155.9(2)

[1]1-x,+y,1/2-z; [2]-x,+y,1/2-z

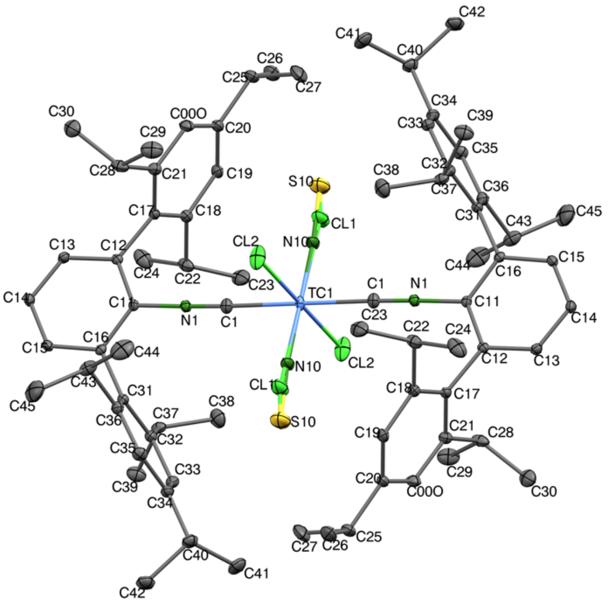

Figure S11. Ellipsoid representation of the structure of [Tc(NS)Cl_3($CNAr^{Tripp2}$)$_2$] (**9**), also illustrating the disorder found for the atoms in the axial positions. The thermal ellipsoids are set at a 30% probability level. Hydrogen atoms are omitted for clarity.

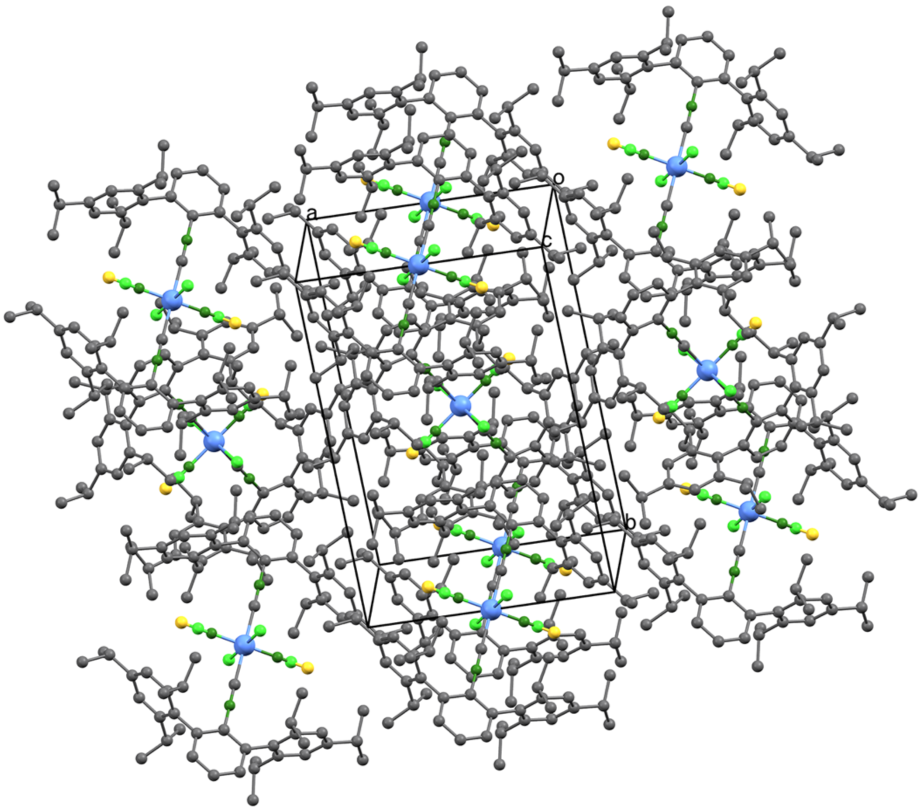

Figure S12. Unit cell plot of [Tc(NS)Cl_3($CNAr^{Tripp2}$)$_2$] (**9**).

Table S12. Bond lengths (Å) in $[Tc(NS)Cl_3(CNAr^{Tripp2})_2]$ (**9**).

Tc1	Cl2[1]	2.3393(4)	C15	C14	1.387(2)
Tc1	Cl2	2.3394(4)	C21	C00O	1.390(2)
Tc1	Cl1	2.4223(15)	C21	C28	1.521(2)
Tc1	C1	2.1087(15)	C22	C18	1.524(2)
Tc1	C1[1]	2.1087(15)	C22	C23	1.531(2)
Tc1	N10[1]	1.707(4)	C22	C24	1.533(3)
Tc1	N10	1.707(4)	C14	C13	1.389(2)
Cl1	S10	0.8480(15)	C34	C40	1.522(2)
Cl1	N10	0.718(3)	C34	C35	1.394(2)
S10	N10	1.540(4)	C37	C38	1.530(2)
N1	C11	1.4035(17)	C37	C39	1.526(2)
N1	C1	1.1447(19)	C18	C19	1.398(2)
C31	C16	1.4981(19)	C19	C20	1.394(2)
C31	C32	1.406(2)	C20	C00O	1.388(2)
C31	C36	1.406(2)	C20	C25	1.528(2)
C16	C15	1.3927(19)	C40	C42	1.534(2)
C16	C11	1.4001(19)	C40	C41	1.528(2)
C12	C17	1.4930(18)	C36	C35	1.393(2)
C12	C11	1.4004(19)	C36	C43	1.523(2)
C12	C13	1.3913(19)	C28	C29	1.534(2)
C32	C33	1.397(2)	C28	C30	1.529(2)
C32	C37	1.525(2)	C43	C44	1.529(3)
C33	C34	1.389(2)	C43	C45	1.526(3)
C17	C21	1.405(2)	C25	C26	1.516(3)
C17	C18	1.404(2)	C25	C27	1.529(3)

[1]1-x,1-y,1-z

Table S13. Bond angles (°) in $[Tc(NS)Cl_3(CNAr^{Tripp2})_2]$ (**9**).

Cl2[1]	Tc1	Cl2	180.0	C00O	C21	C17	118.21(14)
Cl2[1]	Tc1	Cl1	90.29(3)	C00O	C21	C28	120.22(13)
Cl2	Tc1	Cl1	89.71(3)	C16	C11	N1	117.97(12)
C1	Tc1	Cl2	89.23(4)	C16	C11	C12	123.70(12)
C1	Tc1	Cl2[1]	90.77(4)	C12	C11	N1	118.31(12)
C1[1]	Tc1	Cl2[1]	89.23(4)	C18	C22	C23	113.22(13)
C1[1]	Tc1	Cl2	90.77(4)	C18	C22	C24	110.30(14)
C1	Tc1	Cl1	89.66(5)	C23	C22	C24	111.21(14)
C1[1]	Tc1	Cl1	90.34(5)	N1	C1	Tc1	177.31(13)
C1[1]	Tc1	C1	180.0	C15	C14	C13	120.51(13)
N10[1]	Tc1	Cl2[1]	88.09(11)	C14	C13	C12	120.89(13)
N10	Tc1	Cl2	88.10(11)	C33	C34	C40	122.46(14)
N10[1]	Tc1	Cl2	91.90(11)	C33	C34	C35	118.20(14)
N10	Tc1	Cl2[1]	91.90(11)	C35	C34	C40	119.33(14)
N10[1]	Tc1	Cl1	178.21(11)	C32	C37	C38	111.29(13)
N10	Tc1	Cl1	1.79(11)	C32	C37	C39	111.80(13)
N10	Tc1	C1	88.85(10)	C39	C37	C38	110.47(13)
N10	Tc1	C1[1]	91.15(10)	C17	C18	C22	120.85(13)
N10[1]	Tc1	C1[1]	88.85(10)	C19	C18	C17	118.55(13)
N10[1]	Tc1	C1	91.15(10)	C19	C18	C22	120.55(13)
N10[1]	Tc1	N10	180.0	C20	C19	C18	121.65(14)
S10	Cl1	Tc1	163.25(13)	C19	C20	C25	123.07(14)
N10	Cl1	Tc1	4.3(3)	C00O	C20	C19	118.20(13)
N10	Cl1	S10	159.0(3)	C00O	C20	C25	118.70(14)
Cl1	S10	N10	9.62(15)	C20	C00O	C21	122.48(14)

C1	N1	C11	174.56(14)	C34	C40	C42	110.16(13)
C32	C31	C16	119.16(12)	C34	C40	C41	113.82(14)
C36	C31	C16	120.20(12)	C41	C40	C42	110.51(14)
C36	C31	C32	120.62(13)	C31	C36	C43	121.33(13)
C15	C16	C31	122.67(12)	C35	C36	C31	118.49(13)
C15	C16	C11	116.80(12)	C35	C36	C43	120.18(14)
C11	C16	C31	120.51(12)	C21	C28	C29	112.34(14)
C11	C12	C17	122.19(12)	C21	C28	C30	110.21(13)
C13	C12	C17	120.87(12)	C30	C28	C29	111.69(14)
C13	C12	C11	116.94(12)	C36	C35	C34	122.13(14)
C31	C32	C37	122.10(13)	C36	C43	C44	112.19(14)
C33	C32	C31	118.59(13)	C36	C43	C45	110.64(15)
C33	C32	C37	119.30(13)	C45	C43	C44	111.24(17)
C34	C33	C32	121.96(14)	C20	C25	C27	109.35(14)
C21	C17	C12	119.47(12)	C26	C25	C20	114.81(14)
C18	C17	C12	119.59(12)	C26	C25	C27	110.38(15)
C18	C17	C21	120.86(13)	Cl1	N10	Tc1	173.9(4)
C14	C15	C16	121.01(13)	Cl1	N10	S10	11.39(18)
C17	C21	C28	121.53(13)	S10	N10	Tc1	174.7(2)

[1]1-x,1-y,1-z

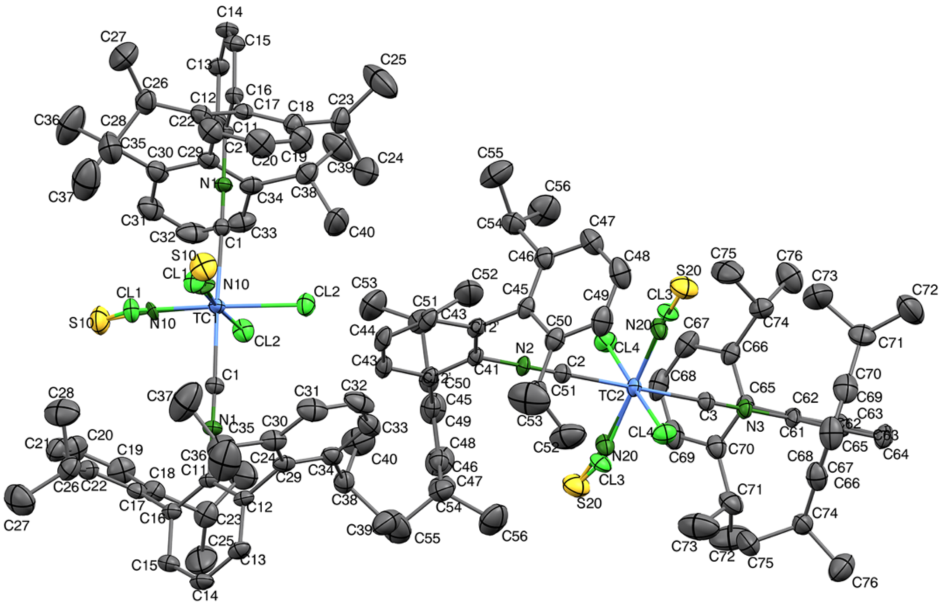

Figure S13. Ellipsoid representations of both crystallogrphically independent molecules of [Tc(NS)Cl_3(CNAr^{Dipp2})$_2$] (**10**) also illustrating the positional disorders inside the coordination spheres of the technetium atoms. The thermal ellipsoids are set at a 50% probability level. Hydrogen atoms are omitted for clarity.

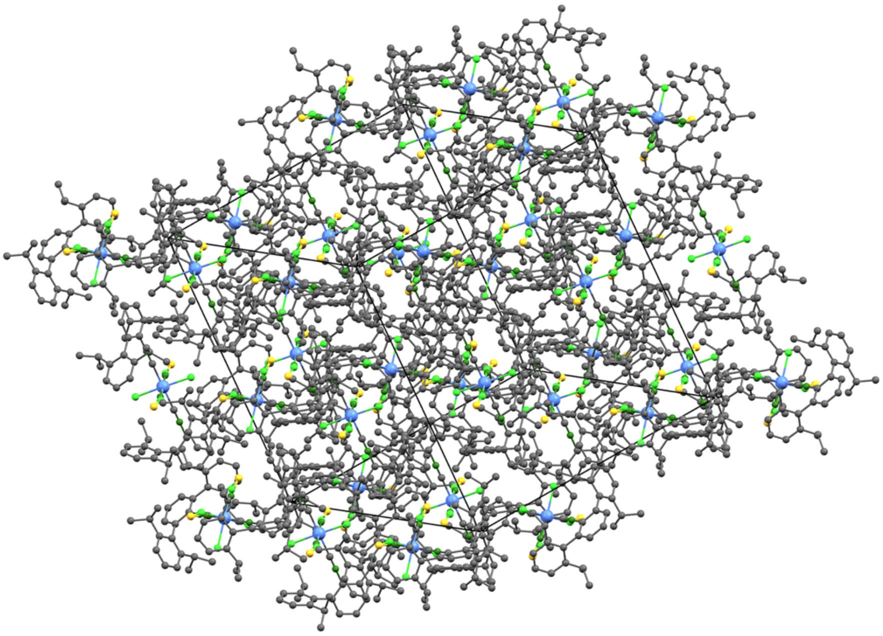

Figure S14. Unit cell plot of [Tc(NS)Cl$_3$(CNAr^{Dipp2})$_2$] (**10**).

Table S14. Bond lengths (Å) in [Tc(NS)Cl$_3$(CNAr^{Dipp2})$_2$] (**10**).

Tc1	Cl2^{1}	2.3682(9)	Tc2	C2	2.111(4)
Tc1	Cl2	2.3682(9)	Tc2	C3	2.131(4)
Tc1	C1	2.116(3)	Tc2	Cl3	2.309(2)
Tc1	C1^{1}	2.116(3)	Tc2	Cl3^{1}	2.309(2)
Tc1	Cl1^{1}	2.292(3)	Tc2	N20^{1}	1.814(9)
Tc1	Cl1	2.292(3)	Tc2	N20	1.814(9)
Tc1	N10	1.775(9)	N2	C2	1.151(5)
Tc1	N10^{1}	1.775(9)	N2	C41	1.406(5)
N1	C1	1.133(3)	N3	C3	1.134(5)
N1	C11	1.401(4)	N3	C61	1.408(5)
C11	C12	1.397(4)	C41	C12'^{1}	1.396(4)
C11	C16	1.393(4)	C41	C12'	1.396(4)
C12	C13	1.390(4)	C12'	C43	1.394(4)
C12	C29	1.497(4)	C12'	C45	1.492(4)
C13	C14	1.380(5)	C43	C44	1.371(4)
C14	C15	1.369(5)	C45	C46	1.405(5)
C15	C16	1.397(4)	C45	C50	1.406(5)
C16	C17	1.490(5)	C46	C47	1.390(5)
C17	C18	1.401(5)	C46	C54	1.511(6)
C17	C22	1.403(5)	C47	C48	1.368(6)
C18	C19	1.381(5)	C48	C49	1.367(6)
C18	C23	1.522(5)	C49	C50	1.385(5)
C19	C20	1.374(6)	C50	C51	1.517(5)
C20	C21	1.367(6)	C54	C55	1.511(6)
C21	C22	1.384(5)	C54	C56	1.532(6)
C22	C26	1.517(5)	C51	C52	1.508(6)
C23	C24	1.490(6)	C51	C53	1.504(7)
C23	C25	1.499(6)	C61	C62^{1}	1.386(4)
C26	C27	1.515(6)	C61	C62	1.386(4)
C26	C28	1.526(6)	C62	C63	1.403(4)
C29	C30	1.405(5)	C62	C65	1.494(5)
C29	C34	1.405(5)	C63	C64	1.374(4)
C30	C31	1.396(5)	C65	C70	1.405(4)

C30	C35	1.515(6)	C65	C66	1.404(5)
C31	C32	1.365(6)	C70	C69	1.392(6)
C32	C33	1.361(6)	C70	C71	1.517(6)
C33	C34	1.396(5)	C69	C68	1.358(6)
C34	C38	1.516(5)	C68	C67	1.376(6)
C35	C36	1.497(7)	C67	C66	1.382(5)
C35	C37	1.500(7)	C66	C74	1.513(5)
C38	C39	1.507(6)	C71	C72	1.512(6)
C38	C40	1.537(6)	C71	C73	1.508(7)
S10	N10	1.552(9)	C74	C76	1.507(6)
Tc2	Cl4^{1}	2.3315(9)	C74	C75	1.526(5)
Tc2	Cl4	2.3316(9)	S20	N20	1.553(9)

^{1}1-x,+y,3/2-z

Table S15. Bond angles (°) in [Tc(NS)Cl$_3$(CNAr^{Dipp2})$_2$] (**10**).

Cl2^{1}	Tc1	Cl2	88.05(4)	C2	Tc2	Cl3^{1}	90.49(15)
C1	Tc1	Cl2	89.77(8)	C3	Tc2	Cl4	89.16(3)
C1^{1}	Tc1	Cl2	88.60(8)	C3	Tc2	Cl4^{1}	89.17(3)
C1^{1}	Tc1	Cl2^{1}	89.77(8)	C3	Tc2	Cl3^{1}	89.51(15)
C1	Tc1	Cl2^{1}	88.60(8)	C3	Tc2	Cl3	89.51(15)
C1^{1}	Tc1	C1	177.73(17)	Cl3^{1}	Tc2	Cl4	90.65(13)
C1^{1}	Tc1	Cl1	90.37(14)	Cl3	Tc2	Cl4	89.34(13)
C1	Tc1	Cl1	91.19(14)	Cl3^{1}	Tc2	Cl4^{1}	89.33(13)
C1^{1}	Tc1	Cl1^{1}	91.19(14)	Cl3	Tc2	Cl4^{1}	90.65(13)
C1	Tc1	Cl1^{1}	90.37(14)	Cl3	Tc2	Cl3^{1}	179.0(3)
Cl1^{1}	Tc1	Cl2	89.72(10)	N20^{1}	Tc2	Cl4	92.4(5)
Cl1	Tc1	Cl2	177.55(11)	N20	Tc2	Cl4	87.6(5)
Cl1	Tc1	Cl2^{1}	89.72(10)	N20^{1}	Tc2	C2	88.4(5)
Cl1^{1}	Tc1	Cl2^{1}	177.55(11)	N20	Tc2	C2	88.4(5)
Cl1	Tc1	Cl1^{1}	92.5(2)	N20	Tc2	C3	91.6(5)
N10^{1}	Tc1	Cl2	85.8(4)	N20^{1}	Tc2	C3	91.6(5)
N10	Tc1	Cl2	173.7(4)	N20^{1}	Tc2	N20	176.7(10)
N10	Tc1	C1	91.4(4)	C2	N2	C41	180.0
N10^{1}	Tc1	C1	90.0(4)	C3	N3	C61	180.0
C1	N1	C11	179.1(3)	N2	C2	Tc2	180.0
N1	C1	Tc1	178.6(3)	N3	C3	Tc2	180.0
C12	C11	N1	118.1(3)	C12'^{1}	C41	N2	118.00(19)
C16	C11	N1	117.9(3)	C12'	C41	N2	118.00(19)
C16	C11	C12	124.0(3)	C12'^{1}	C41	C12'	124.0(4)
C11	C12	C29	122.2(3)	C41	C12'	C45	121.0(3)
C13	C12	C11	116.9(3)	C43	C12'	C41	116.4(3)
C13	C12	C29	120.9(3)	C43	C12'	C45	122.5(3)
C14	C13	C12	120.2(3)	C44	C43	C12'	120.9(4)
C15	C14	C13	121.8(3)	C43^{1}	C44	C43	121.3(4)
C14	C15	C16	120.5(3)	C46	C45	C12'	119.9(3)
C11	C16	C15	116.6(3)	C46	C45	C50	120.9(3)
C11	C16	C17	121.6(3)	C50	C45	C12'	119.2(3)
C15	C16	C17	121.8(3)	C45	C46	C54	121.6(3)
C18	C17	C16	118.9(3)	C47	C46	C45	118.0(4)
C18	C17	C22	121.4(3)	C47	C46	C54	120.4(4)
C22	C17	C16	119.7(3)	C48	C47	C46	121.0(4)
C17	C18	C23	122.0(3)	C49	C48	C47	120.8(4)
C19	C18	C17	117.9(4)	C48	C49	C50	121.0(4)
C19	C18	C23	120.1(3)	C45	C50	C51	121.6(3)
C20	C19	C18	121.3(4)	C49	C50	C45	118.3(4)
C21	C20	C19	120.2(4)	C49	C50	C51	120.1(4)

C20	C21	C22	121.3(4)	C46	C54	C55	111.5(4)
C17	C22	C26	122.1(3)	C46	C54	C56	111.7(4)
C21	C22	C17	117.9(3)	C55	C54	C56	110.5(4)
C21	C22	C26	120.0(3)	C52	C51	C50	112.6(3)
C24	C23	C18	113.2(4)	C53	C51	C50	113.7(4)
C24	C23	C25	110.2(4)	C53	C51	C52	110.6(4)
C25	C23	C18	113.1(4)	C62[1]	C61	N3	117.79(19)
C22	C26	C28	111.6(4)	C62	C61	N3	117.79(19)
C27	C26	C22	111.7(4)	C62	C61	C62[1]	124.4(4)
C27	C26	C28	111.1(4)	C61	C62	C63	116.7(3)
C30	C29	C12	119.0(3)	C61	C62	C65	122.6(3)
C30	C29	C34	121.5(3)	C63	C62	C65	120.7(3)
C34	C29	C12	119.4(3)	C64	C63	C62	120.2(4)
C29	C30	C35	121.7(3)	C63	C64	C63[1]	121.8(4)
C31	C30	C29	117.3(4)	C70	C65	C62	119.2(3)
C31	C30	C35	121.0(4)	C66	C65	C62	119.7(3)
C32	C31	C30	121.5(4)	C66	C65	C70	121.1(3)
C33	C32	C31	120.8(4)	C65	C70	C71	121.3(3)
C32	C33	C34	121.1(4)	C69	C70	C65	117.4(4)
C29	C34	C38	121.9(3)	C69	C70	C71	121.3(4)
C33	C34	C29	117.8(3)	C68	C69	C70	122.0(4)
C33	C34	C38	120.3(3)	C69	C68	C67	119.9(4)
C36	C35	C30	114.8(4)	C68	C67	C66	121.3(4)
C36	C35	C37	111.2(5)	C65	C66	C74	121.7(3)
C37	C35	C30	111.6(4)	C67	C66	C65	118.2(3)
C34	C38	C40	112.1(3)	C67	C66	C74	120.2(3)
C39	C38	C34	112.8(3)	C72	C71	C70	114.0(4)
C39	C38	C40	109.8(4)	C73	C71	C70	112.7(4)
S10	N10	Tc1	170.2(8)	C73	C71	C72	110.3(4)
Cl4[1]	Tc2	Cl4	178.33(5)	C66	C74	C75	112.2(3)
C2	Tc2	Cl4	90.84(3)	C76	C74	C66	112.4(3)
C2	Tc2	Cl4[1]	90.83(3)	C76	C74	C75	110.3(4)
C2	Tc2	C3	180.0	S20	N20	Tc2	166.9(10)
C2	Tc2	Cl3	90.49(15)				

[1]1-x,+y,3/2-z

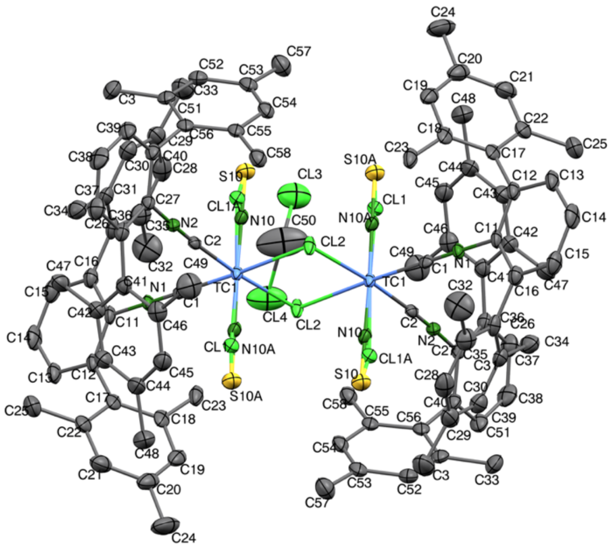

Figure S15. Ellipsoid representation of the structure of [{Tc(NS)Cl(CNAr^{Mes2})$_2$}$_2$Cl$_2$] (**12**) x 2 CH_2Cl_2, also illustrating the disorder of the axial ligands. The thermal ellipsoids are set at a 50% probability level. Hydrogen atoms are omitted for clarity.

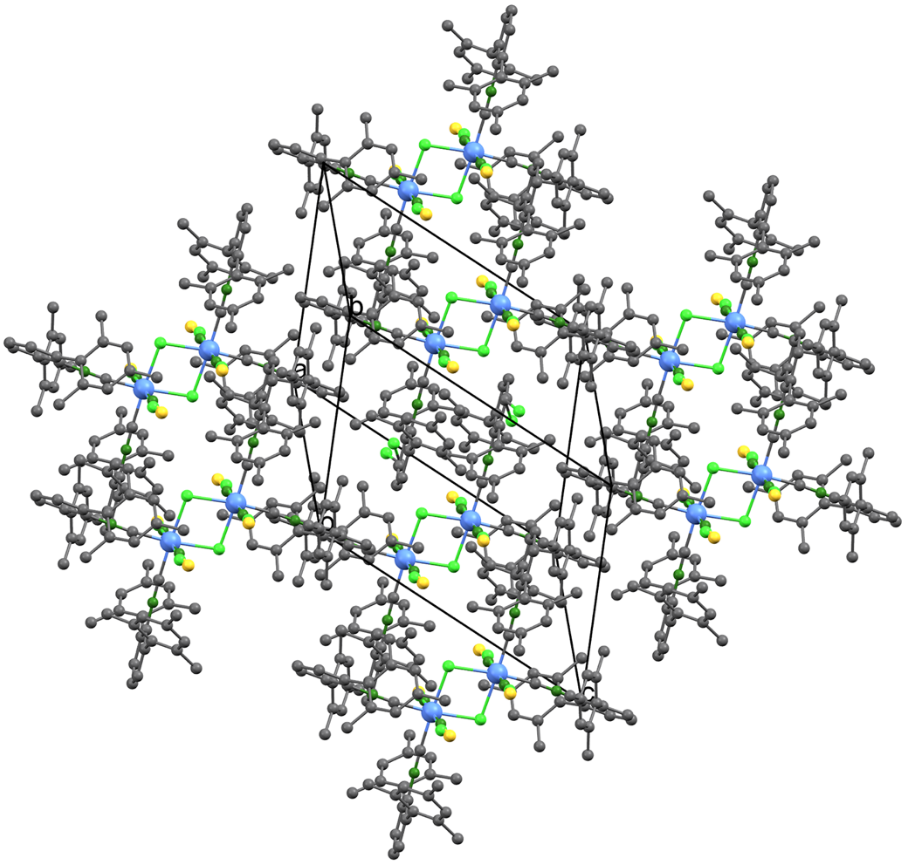

Figure S16. Unit cell plot of $[\{Tc(NS)Cl(CNAr^{Mes2})_2\}_2Cl_2]$ (**12**) x 2 CH_2Cl_2.

Table S16. Bond lengths (Å) in $[\{Tc(NS)Cl(CNAr^{Mes2})_2\}_2Cl_2]$ (**12**) x 2 CH_2Cl_2.

Tc1	Cl2[1]	2.4881(12)	C26	C16	1.498(9)
Tc1	Cl2	2.4768(13)	C26	C31	1.391(9)
Tc1	Cl1A	2.390(2)	C26	C27	1.406(10)
Tc1	N10A	1.759(7)	C55	C54	1.382(9)
Tc1	C1	1.996(5)	C55	C58	1.506(9)
Tc1	C2	1.993(5)	C40	C39	1.384(9)
Tc1	Cl1	2.344(7)	C51	C52	1.379(9)
Tc1	N10	1.770(11)	C51	C56	1.516(9)
Cl1A	S10	0.938(7)	C19	C20	1.372(12)
Cl1A	N10	0.635(11)	C12	C11	1.401(8)
S10A	N10A	1.553(7)	C12	C13	1.400(8)
S10A	Cl1	1.003(7)	C11	C16	1.403(9)
Cl3	C50	1.637(14)	C16	C15	1.393(9)
Cl4	C50	1.825(14)	C14	C13	1.382(11)
N10A	Cl1	0.607(7)	C14	C15	1.384(11)
N2	C2	1.149(7)	C21	C20	1.400(12)
N2	C35	1.393(7)	C30	C29	1.383(10)
N1	C1	1.159(7)	C30	C31	1.401(10)
N1	C11	1.399(7)	C37	C38	1.393(10)
C18	C17	1.416(9)	C42	C41	1.397(9)
C18	C19	1.388(10)	C42	C47	1.511(9)
C18	C23	1.506(10)	C42	C43	1.390(9)
C35	C36	1.400(8)	C45	C46	1.391(10)

C35	C40	1.407(8)	C45	C44	1.376(11)
C17	C22	1.408(10)	C41	C46	1.409(9)
C17	C12	1.492(9)	C29	C33	1.502(11)
C3	C55	1.414(8)	C29	C28	1.397(12)
C3	C40	1.484(9)	C34	C31	1.500(9)
C3	C51	1.415(9)	C39	C38	1.378(10)
C53	C54	1.367(10)	C20	C24	1.528(11)
C53	C52	1.402(9)	C46	C49	1.517(10)
C53	C57	1.501(10)	C27	C28	1.392(11)
C22	C21	1.392(10)	C27	C32	1.503(12)
C22	C25	1.517(10)	C44	C43	1.395(10)
C36	C37	1.373(9)	C44	C48	1.520(10)
C36	C41	1.497(9)	S10	N10	1.537(12)

[1]1-x,-y,1-z

Table S17. Bond angles (°) in [{Tc(NS)Cl(CNAr^{Mes2})$_2$}$_2$Cl$_2$] (**12**) x 2 CH_2Cl_2.

Cl2	Tc1	Cl2[1]	84.58(4)	C3	C55	C58	120.9(6)
Cl1A	Tc1	Cl2[1]	88.97(6)	C54	C55	C3	119.2(6)
Cl1A	Tc1	Cl2	91.22(6)	C54	C55	C58	119.8(6)
N10A	Tc1	Cl2[1]	94.1(2)	C35	C40	C3	119.3(5)
N10A	Tc1	Cl2	88.3(2)	C39	C40	C35	116.3(6)
N10A	Tc1	Cl1A	176.8(2)	C39	C40	C3	124.3(6)
N10A	Tc1	C1	89.9(3)	C3	C51	C56	120.5(6)
N10A	Tc1	C2	92.0(3)	C52	C51	C3	119.2(6)
N10A	Tc1	Cl1	4.5(3)	C52	C51	C56	120.3(6)
N10A	Tc1	N10	177.2(7)	C20	C19	C18	123.4(7)
C1	Tc1	Cl2[1]	175.56(15)	C11	C12	C17	123.8(5)
C1	Tc1	Cl2	93.60(14)	C13	C12	C17	119.9(6)
C1	Tc1	Cl1A	87.02(15)	C13	C12	C11	116.3(6)
C1	Tc1	Cl1	91.2(2)	N1	C11	C12	118.3(5)
C2	Tc1	Cl2[1]	89.21(14)	N1	C11	C16	117.7(5)
C2	Tc1	Cl2	173.78(14)	C12	C11	C16	124.0(5)
C2	Tc1	Cl1A	88.82(15)	C11	C16	C26	121.7(5)
C2	Tc1	C1	92.61(19)	C15	C16	C26	121.5(6)
C2	Tc1	Cl1	87.6(2)	C15	C16	C11	116.8(6)
Cl1	Tc1	Cl2[1]	92.88(18)	C13	C14	C15	120.8(6)
Cl1	Tc1	Cl2	92.51(18)	C53	C54	C55	122.5(6)
Cl1	Tc1	Cl1A	175.98(18)	C14	C13	C12	121.2(6)
N10	Tc1	Cl2[1]	85.6(7)	C22	C21	C20	121.4(8)
N10	Tc1	Cl2	88.9(7)	C29	C30	C31	122.0(7)
N10	Tc1	Cl1A	4.0(7)	C36	C37	C38	121.1(6)
N10	Tc1	C1	90.4(7)	C41	C42	C47	121.0(6)
N10	Tc1	C2	90.8(7)	C43	C42	C41	118.7(6)
N10	Tc1	Cl1	177.8(8)	C43	C42	C47	120.3(6)
Tc1	Cl2	Tc1[1]	95.43(4)	C44	C45	C46	122.2(7)
S10	Cl1A	Tc1	164.8(4)	C51	C52	C53	121.8(6)
N10	Cl1A	Tc1	11(2)	C42	C41	C36	121.1(6)
N10	Cl1A	S10	155(2)	C42	C41	C46	120.2(6)
Cl1	S10A	N10A	11.8(5)	C46	C41	C36	118.5(6)
S10A	N10A	Tc1	177.5(5)	C30	C29	C33	121.5(8)
Cl1	N10A	Tc1	162.2(14)	C30	C29	C28	117.9(7)
Cl1	N10A	S10A	19.8(10)	C28	C29	C33	120.6(8)
C2	N2	C35	178.1(5)	C14	C15	C16	120.9(7)
C1	N1	C11	178.5(6)	C38	C39	C40	121.0(6)

N1	C1	Tc1	177.9(5)	C26	C31	C30	119.4(6)
C17	C18	C23	123.0(6)	C26	C31	C34	121.6(6)
C19	C18	C17	117.7(7)	C30	C31	C34	119.0(6)
C19	C18	C23	119.3(6)	C19	C20	C21	118.2(7)
N2	C2	Tc1	175.5(4)	C19	C20	C24	122.2(8)
N2	C35	C36	118.0(5)	C21	C20	C24	119.6(9)
N2	C35	C40	117.9(5)	C45	C46	C41	118.8(6)
C36	C35	C40	124.1(5)	C45	C46	C49	119.9(7)
C18	C17	C12	120.6(6)	C41	C46	C49	121.3(7)
C22	C17	C18	120.4(6)	C39	C38	C37	120.8(6)
C22	C17	C12	118.9(5)	C26	C27	C32	121.3(7)
C55	C3	C40	119.7(6)	C28	C27	C26	119.5(7)
C55	C3	C51	119.0(6)	C28	C27	C32	119.2(8)
C51	C3	C40	121.2(5)	C45	C44	C43	117.9(7)
C54	C53	C52	118.2(6)	C45	C44	C48	121.4(7)
C54	C53	C57	121.6(6)	C43	C44	C48	120.6(7)
C52	C53	C57	120.1(7)	C42	C43	C44	122.2(7)
C17	C22	C25	122.1(6)	C27	C28	C29	121.6(7)
C21	C22	C17	118.9(7)	Cl3	C50	Cl4	115.4(6)
C21	C22	C25	119.0(7)	Cl1A	S10	N10	10.2(9)
C35	C36	C41	119.6(5)	S10A	Cl1	Tc1	161.5(6)
C37	C36	C35	116.6(6)	N10A	Cl1	Tc1	13.2(10)
C37	C36	C41	123.8(6)	N10A	Cl1	S10A	148.4(15)
C31	C26	C16	121.5(6)	Cl1A	N10	Tc1	165(3)
C31	C26	C27	119.6(6)	Cl1A	N10	S10	15.2(12)
C27	C26	C16	118.8(6)	S10	N10	Tc1	175.2(16)

[1]1-x,-y,1-z

2. Selected Spectroscopic Data

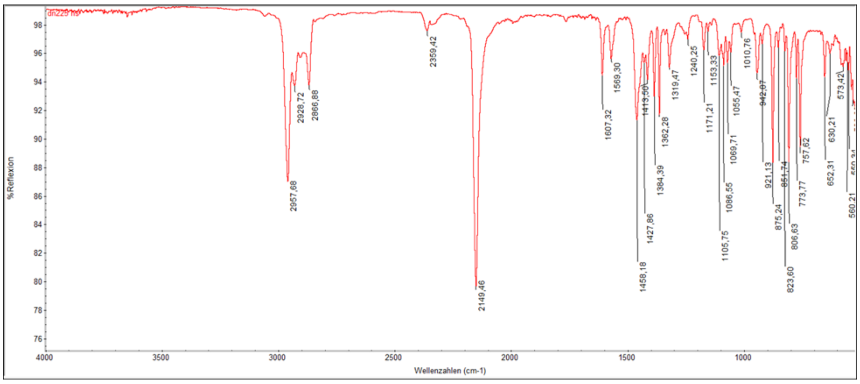

Figure S17. IR spectrum (ATR) of [ReNCl$_2$(CNAr^{Tripp2})$_2$(MeOH)] (**1**).

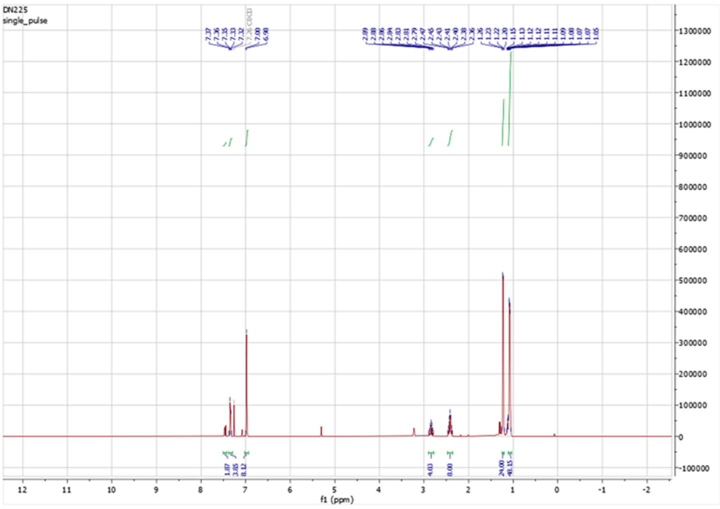

Figure S18. ^{1}H NMR spectrum of [ReNCl$_2$(CNAr^{Tripp2})$_2$(MeOH)] (**1**) in CDCl$_3$.

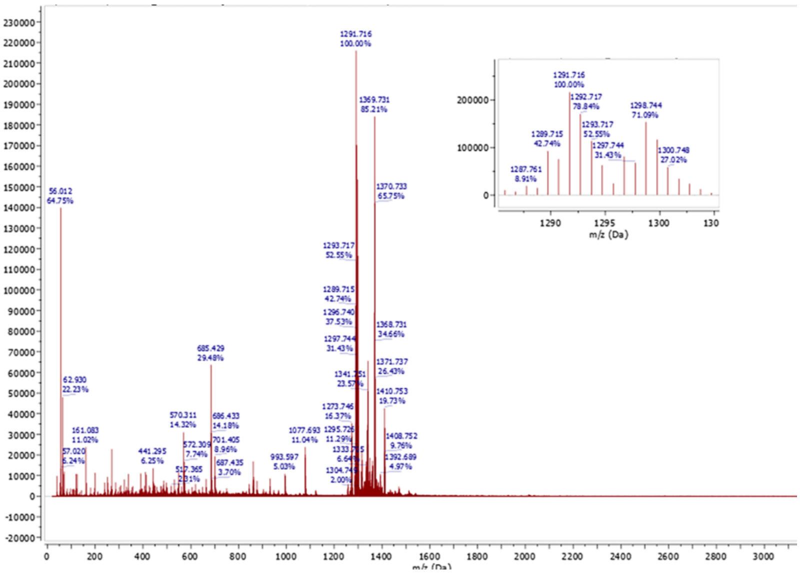

Figure S19. ESI+ mass spectrum of [ReNCl$_2$(CNAr^{Tripp2})$_2$(MeOH)] (**1**) in acetonitrile.

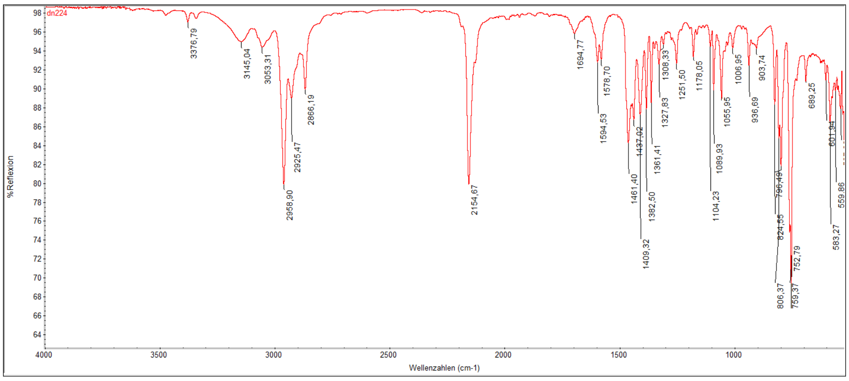

Figure S20. IR spectrum (ATR) of [ReNCl$_2$(CNAr^{Dipp2})$_2$] (**2**).

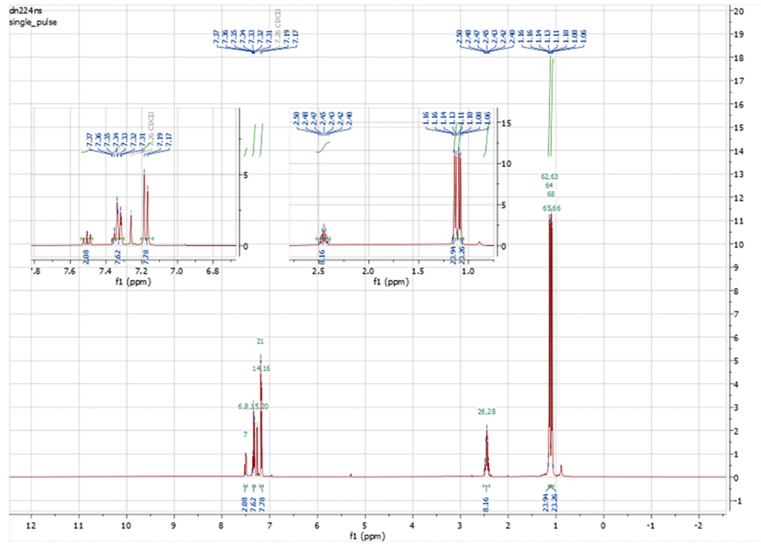

Figure S21. ^{1}H NMR spectrum of [ReNCl$_2$(CNAr^{Dipp2})$_2$] (**2**) in CDCl$_3$.

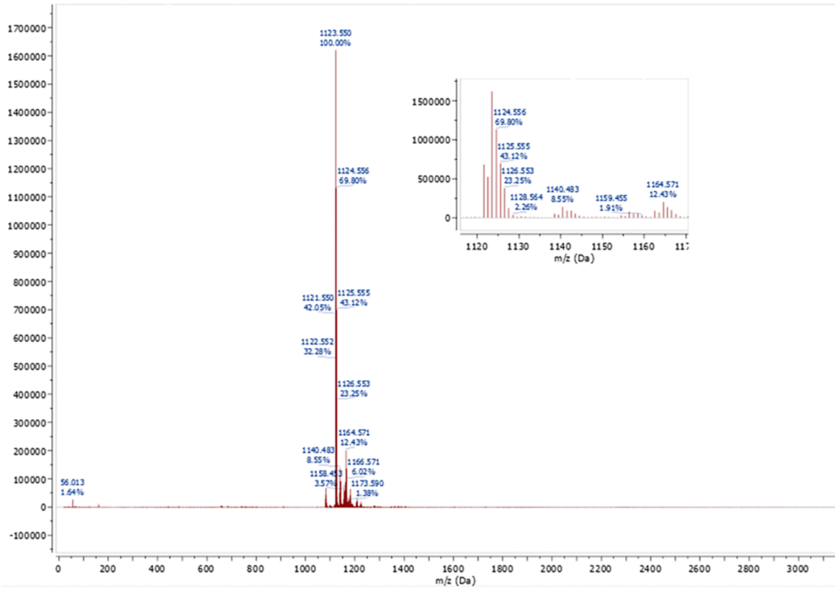

Figure S22. ESI+ mass spectrum of [ReNCl$_2$(CNAr^{Dipp2})$_2$] (**2**) in acetonitrile.

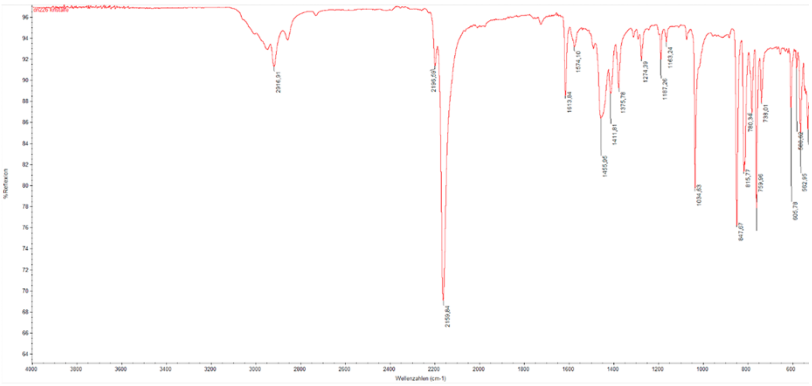

Figure S23. IR spectrum (ATR) of [ReNCl$_2$(CNAr^{Mes2})$_3$] (**3**).

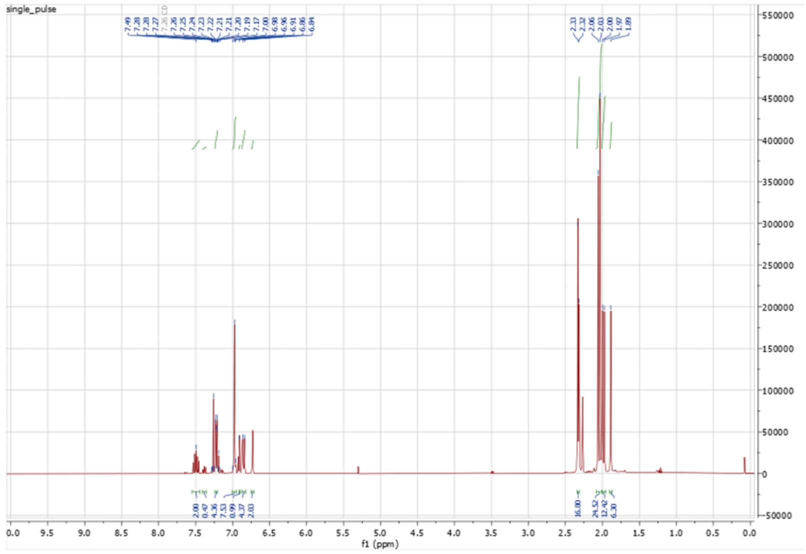

Figure S24. ^{1}H NMR spectrum of [ReNCl$_2$(CNAr^{Mes2})$_3$] (**3**) in CDCl$_3$.

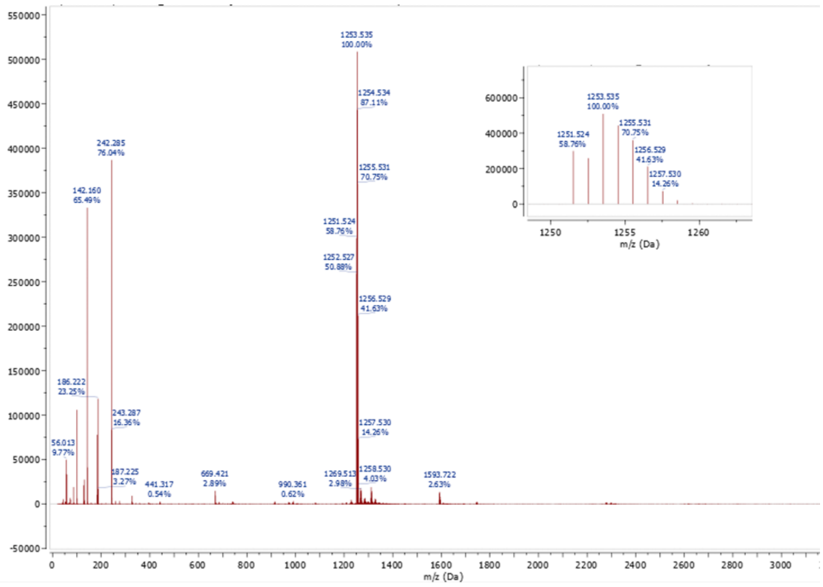

Figure S25. ESI+ mass spectrum of [ReNCl$_2$(CNAr^{Mes2})$_3$] (**3**) in acetonitrile.

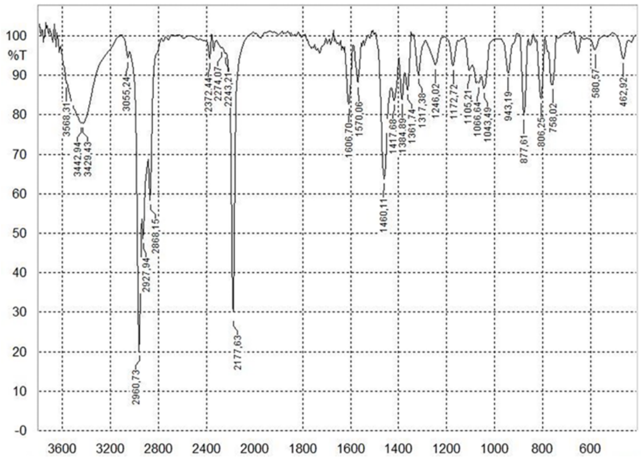

Figure S26. IR spectrum (KBr) of [TcNCl$_2$(CNAr^{Tripp2})$_2$] (**4**).

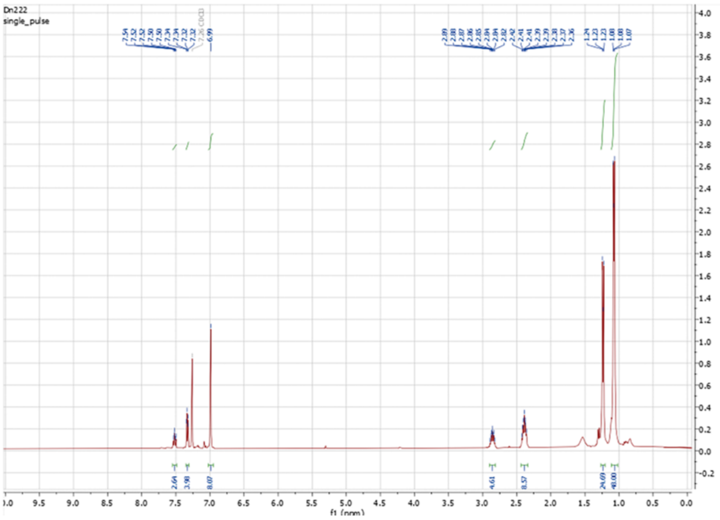

Figure S27. ^{1}H NMR spectrum of [TcNCl$_2$(CNAr^{Tripp2})$_2$] (**4**) in CDCl$_3$.

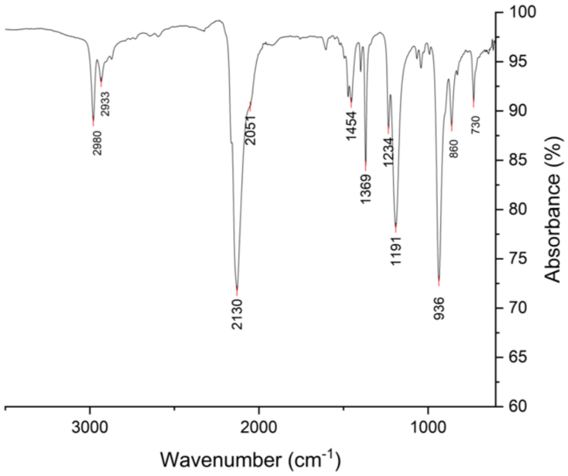

Figure S28. IR spectrum (KBr) of [{TcCl(CN^{t}Bu)$_4$}$_2$(μ-N)]$_2$[TcCl$_6$] (**5**).

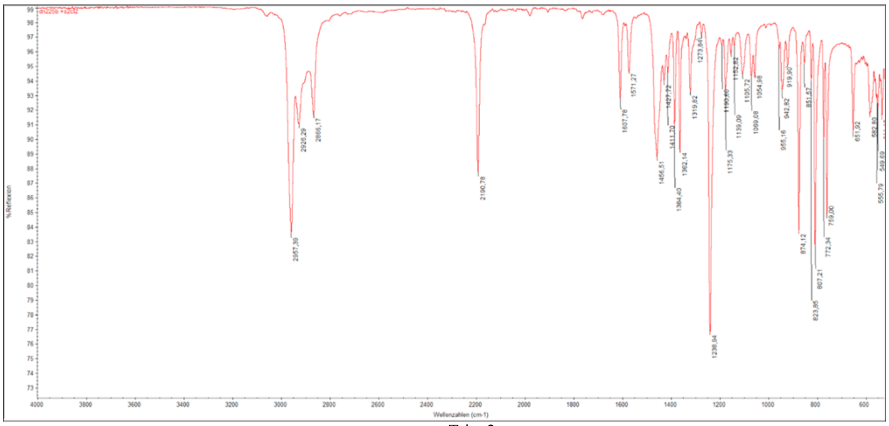

Figure S29. IR spectrum (ATR) of $[Re(NS)Cl_3(CNAr^{Tripp2})_2]$ (**6**).

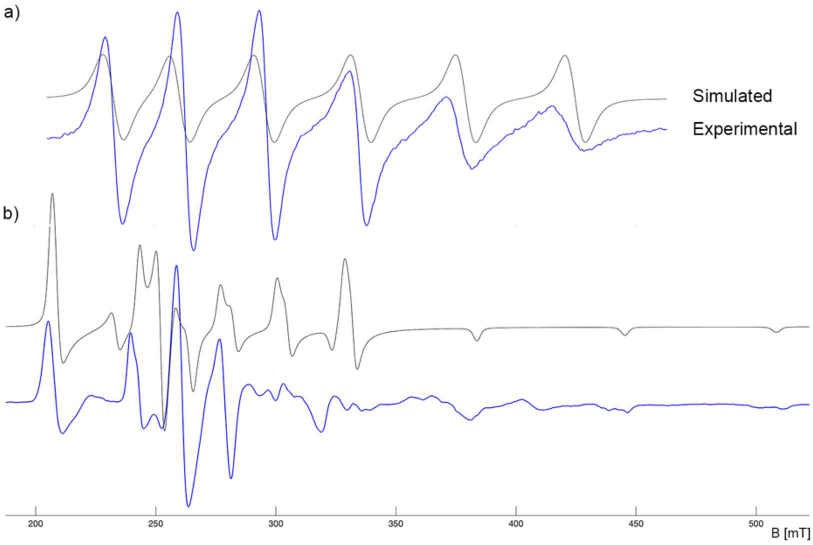

Figure S30. X-Band EPR spectra of $[Re(NS)Cl_3(CNAr^{Tripp2})_2]$ (**6**) in $CHCl_3$ a) at room-temperature and b) at 77 K.

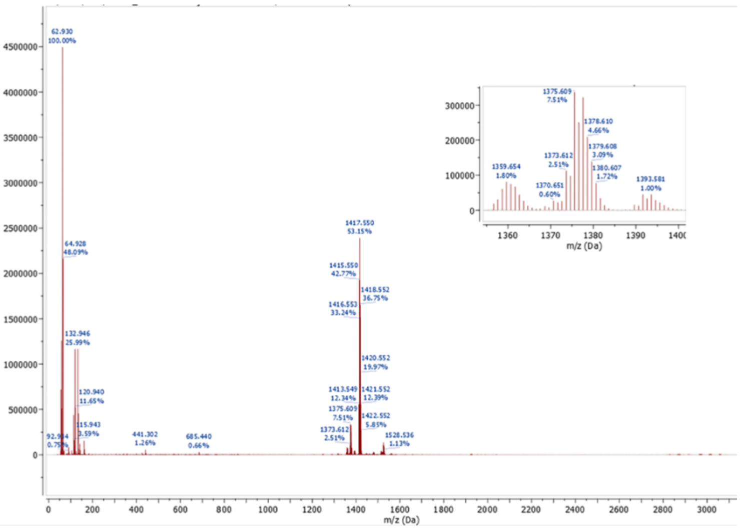

Figure S31. ESI+ mass spectrum of $[Re(NS)Cl_3(CNAr^{Tripp2})_2]$ (**6**) in acetonitrile.

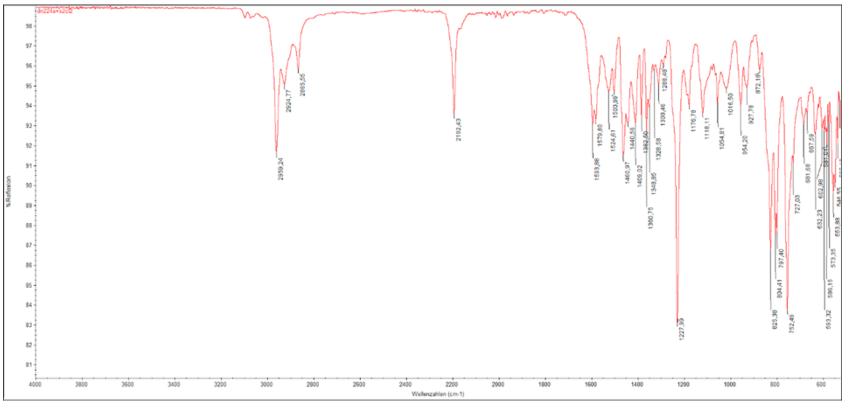

Figure S32. IR spectrum (ATR) of [Re(NS)Cl_3($CNAr^{Dipp2}$)$_2$] (**7**).

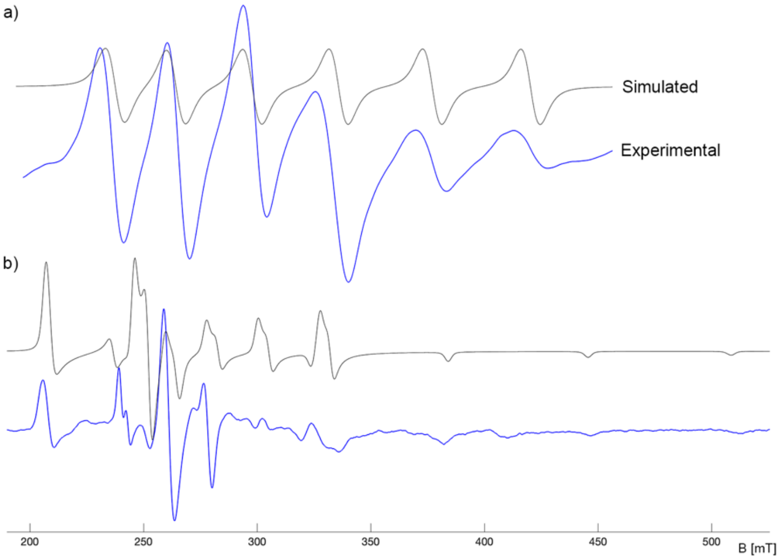

Figure S33. X-Band EPR spectra of [Re(NS)Cl_3($CNAr^{Dipp2}$)$_2$] (**7**) in $CHCl_3$ a) at room-temperature and b) at 77 K.

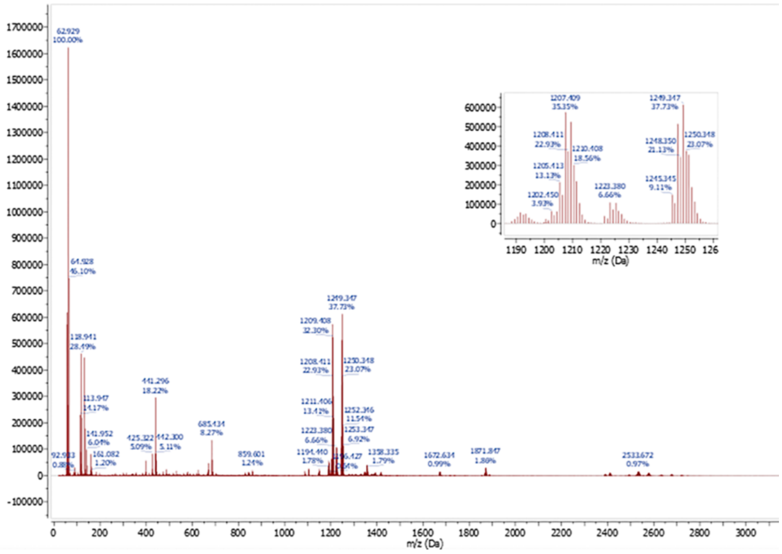

Figure S34. ESI+ mass spectrum of [Re(NS)Cl_3($CNAr^{Dipp2}$)$_2$] (**7**) in acetonitrile.

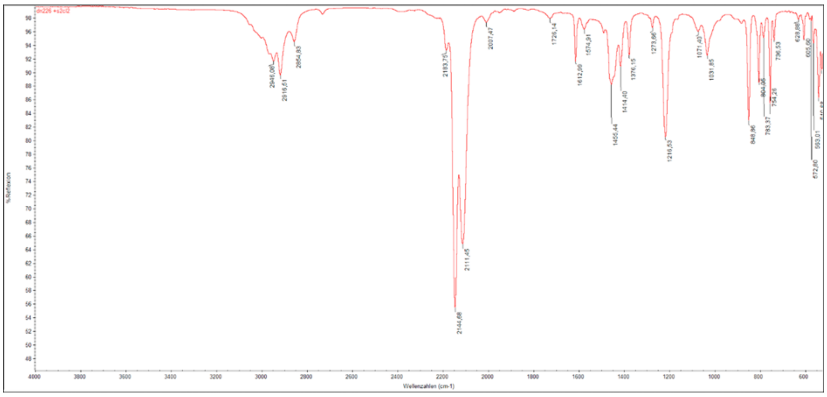

Figure S35. IR spectrum (ATR) of [Re(NS)Cl_2($CNAr^{Mes2}$)$_3$] (**8**).

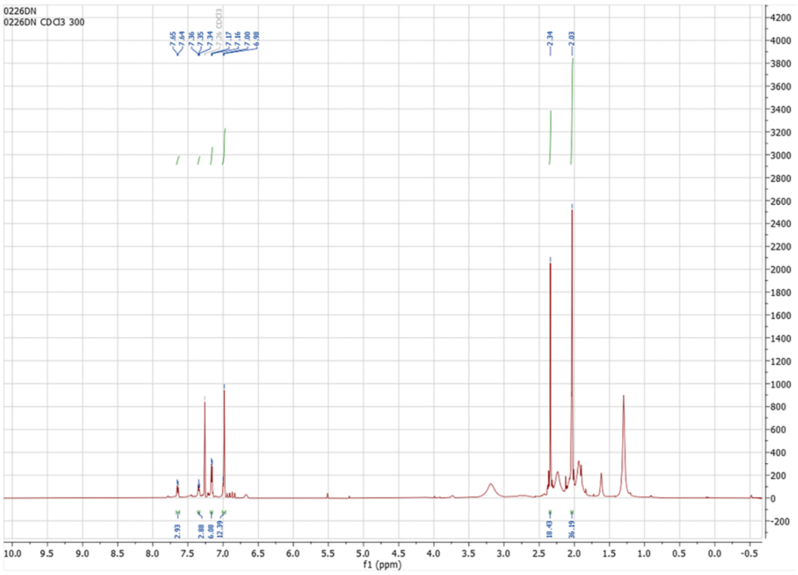

Figure S36. ^{1}H NMR spectrum [Re(NS)Cl_2($CNAr^{Mes2}$)$_3$] (**8**) in $CDCl_3$.

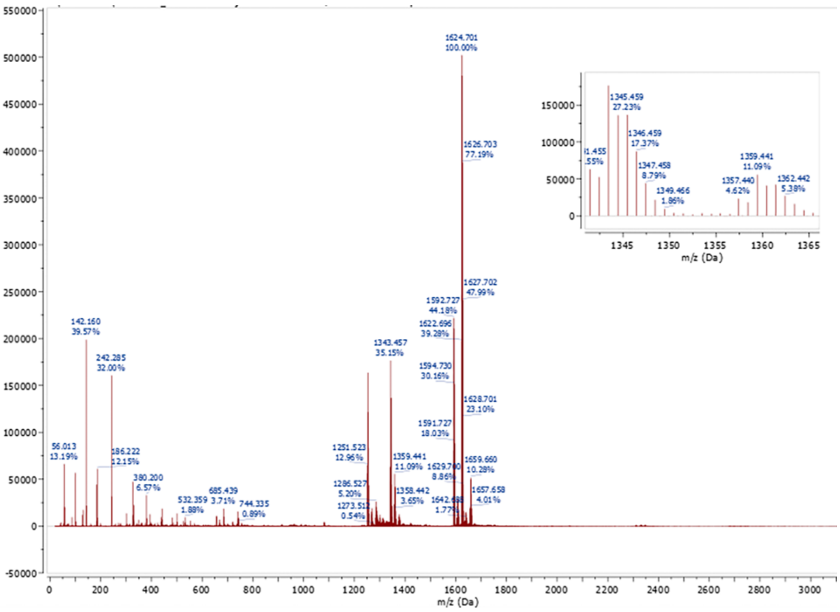

Figure S37. ESI+ mass spectrum of [Re(NS)Cl_2($CNAr^{Mes2}$)$_3$] (**8**) in acetonitrile.

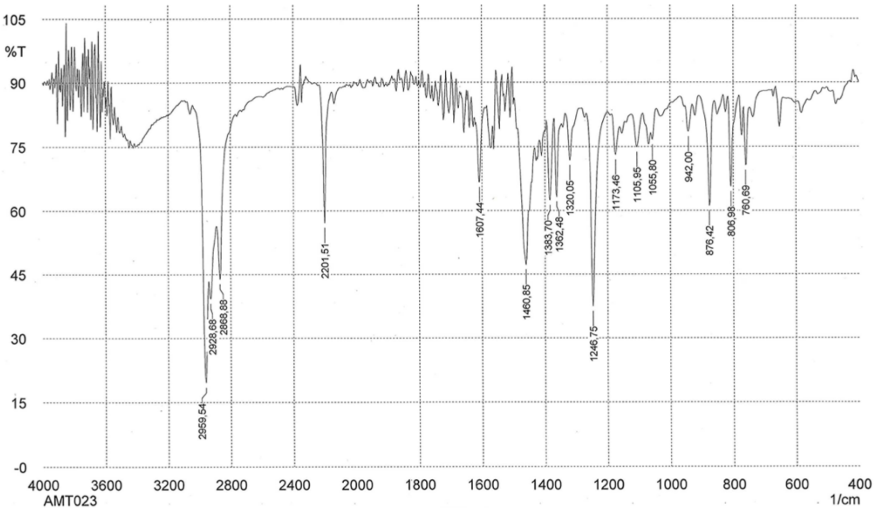

Figure S38. IR spectrum (KBr) of [Tc(NS)Cl_3($CNAr^{Tripp2}$)$_2$] (**9**).

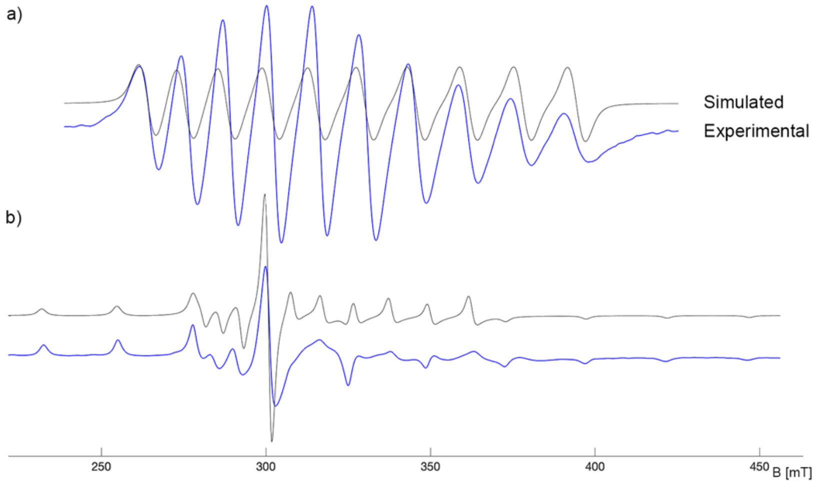

Figure S39. X-Band EPR spectra of [Tc(NS)Cl_3($CNAr^{Tripp2}$)$_2$] (**9**) in CH_2Cl_2 a) at room-temperature and b) at 77 K.

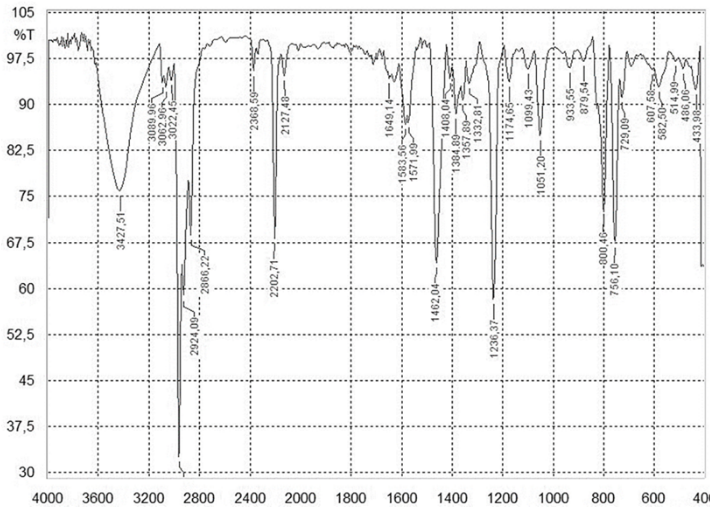

Figure S40. IR spectrum (KBr) of [Tc(NS)Cl_3($CNAr^{Dipp2}$)$_2$] (**10**).

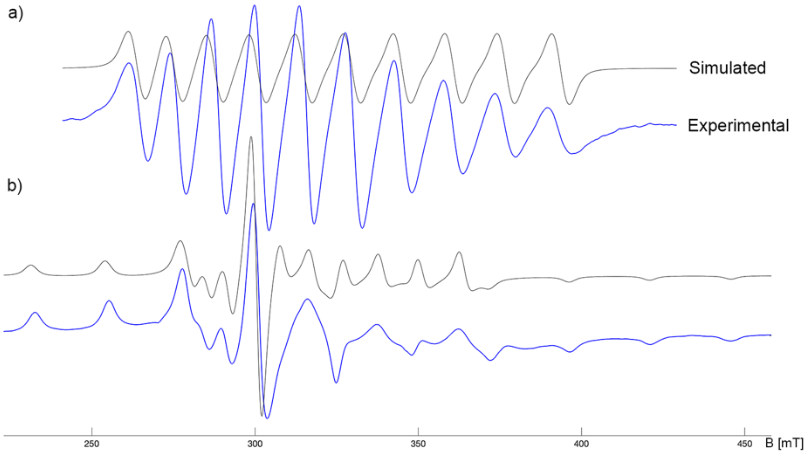

Figure S41. X-Band EPR spectra of [Tc(NS)Cl_3($CNAr^{Dipp2}$)$_2$] (**10**) in CH_2Cl_2 a) at room-temperature and b) at 77 K.

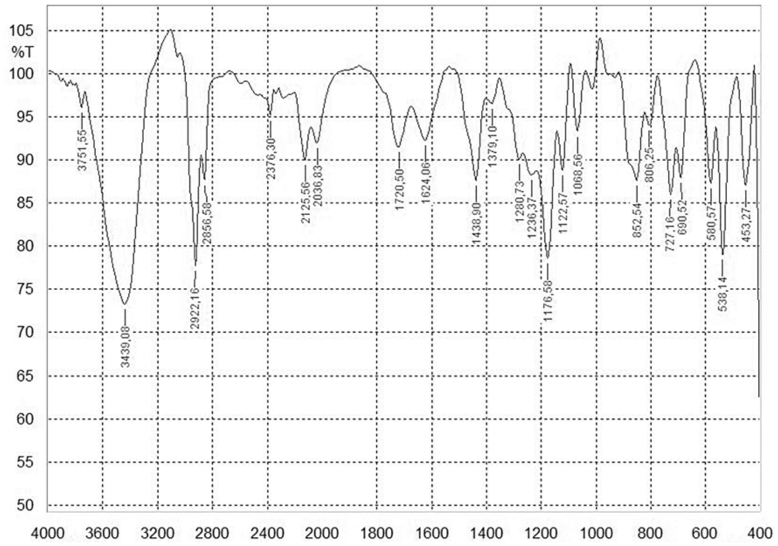

Figure S42. IR spectrum (KBr) of [Tc(NS)Cl_2($CNAr^{Mes2}$)$_3$]Cl (**11**).

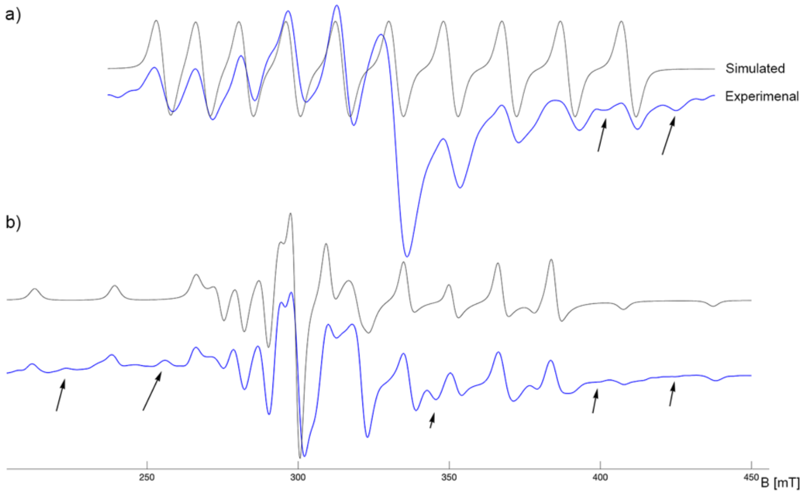

Figure S43. X-Band EPR spectra of [Tc(NS)Cl_2($CNAr^{Mes2}$)$_3$]Cl (**11**) in CH_2Cl_2 a) at room-temperature and b) at 77 K. Arrows indicate the signals of a minor amount of a second Tc(II) complex.

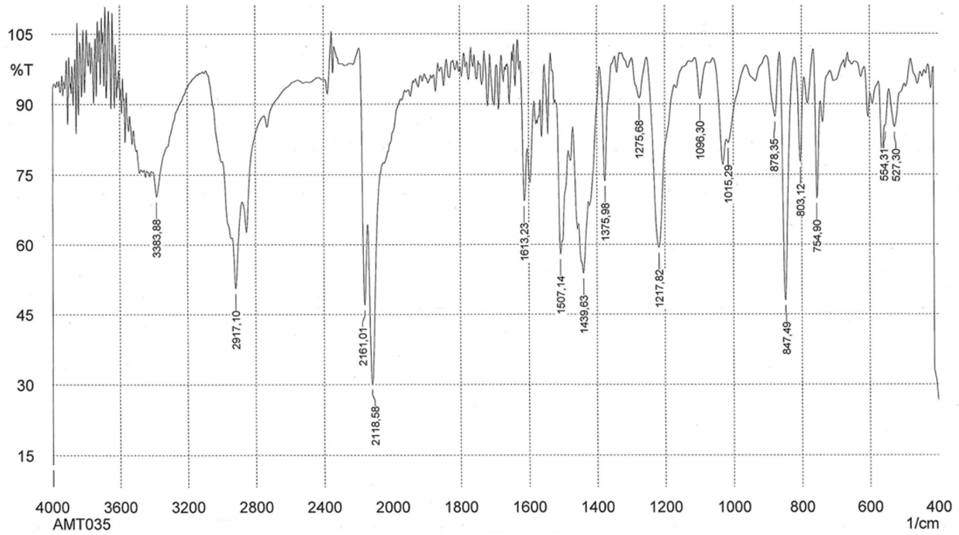

Figure S44. IR spectrum (KBr) of [{Tc(NS)Cl(CNAr^{Mes2})$_2$}$_2$Cl$_2$] (**12**).

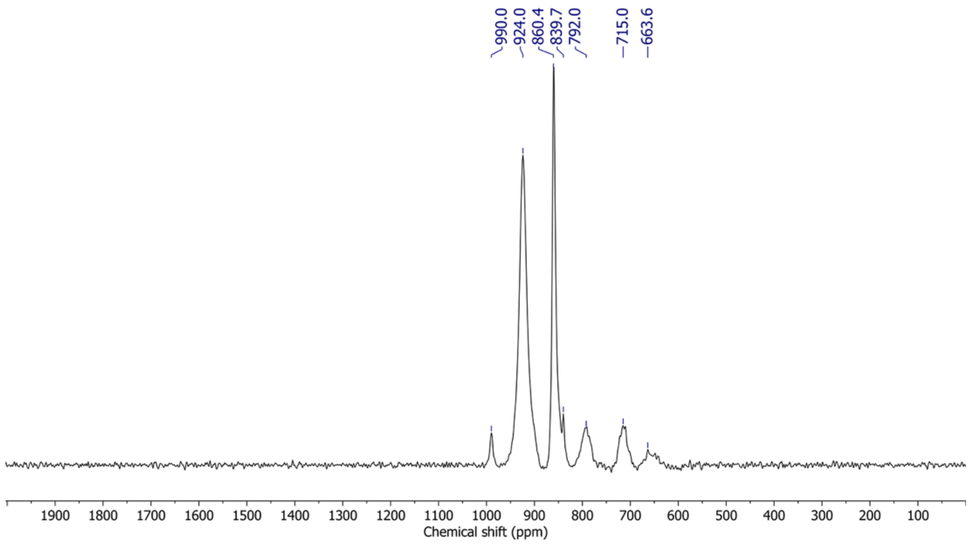

Figure S45. ^{99}Tc NMR spectrum of a reaction mixture between [TcNCl$_2$(CNAr^{Mes2})$_3$] and S_2Cl_2 in CH_2Cl_2, which finally allows the isolation of [{Tc(NS)Cl(CNAr^{Mes2})$_2$}$_2$Cl$_2$] (**12**). Note the intermediate appearance of seven Tc(I) complex species.
